# Mesoporous peptide frameworks engineered from crystallizable collagen-mimetic peptide amphiphiles

**DOI:** 10.1038/s41467-026-73068-2

**Published:** 2026-05-15

**Authors:** Anthony R. Perez, Jianfang Liu, S M Mobin Sikder, Anjan Maity, Adekunle Adewole, Jacob Oakden, Gang Ren, Bercem Dutagaci, Andrea D. Merg

**Affiliations:** 1 Department of Chemistry and Biochemistry, University of California, Merced, Merced, CA USA; 2 The Molecular Foundry, Lawrence Berkeley National Laboratory, Berkeley, CA USA; 3 Department of Molecular and Cell Biology, University of California, Merced, Merced, CA USA; 4 Health Sciences Research Institute, University of California, Merced, Merced, CA USA

**Keywords:** Self-assembly, Molecular self-assembly, Peptides

## Abstract

The rational design of porous frameworks with tunable pore dimensions and chemical functionalities is a critical step toward their implementation in diverse applications. While traditional porous materials are typically constructed from abiotic components, there is increasing interest in employing biologically derived building blocks (*e.g*., peptides and proteins) that offer unmatched structural and functional diversity. Here, we report the construction of crystalline mesoporous frameworks that are self-assembled from amphiphilic collagen-mimetic peptides. Comprehensive structural characterization via microscopy, spectroscopy, and computational techniques provides insights into the assembly packing model, in which hexagonally packed channels are interconnected by antiparallel-aligned collagen triple helices via hydrophobic and electrostatic interactions. Lastly, we demonstrate the functional potential of aCMP frameworks through the encapsulation of various molecular guests, including doxorubicin, an anti-cancer drug. Overall, this work establishes a class of mesoporous frameworks, derived from synthetically engineerable peptide conjugates, marking a significant step forward in broadening the architectural scope and application potential of peptide-based materials.

## Abstract (web summary)

Porous frameworks assembled from biologically derived building blocks have diverse potential applications. Here, the authors report the development of crystalline mesoporous frameworks fabricated from the self-assembly of amphiphilic collagen-mimetic peptides.

## Introduction

Crystalline porous frameworks represent an important class of materials that underpin a broad range of applications^1^. These frameworks, characterized by interconnected networks of porous channels, bestow a vast internal surface area optimal for the encapsulation and separation of various guest species. Over the past several decades, abiotic building blocks (*e.g*., metal ions and organic linkers) have been successfully employed to construct a wide array of extended porous frameworks, as evidenced by the well-established fields of metal-organic frameworks (MOFs) and covalent organic frameworks (COFs)^2–4^. The ability to systematically modulate the physical and chemical features of MOFs and COFs has led to their broad utility in catalysis, sensing, separations, and storage^3–5^.

Despite significant achievements in the development of MOFs and COFs, the employment of biotic building blocks (*e.g*., proteins and peptides) for the design and construction of functional materials is garnering significant traction^6–10^. Biomolecules display structural and functional complexity that, so far, exceeds what is currently achievable with small, synthetic molecules. Furthermore, bio-derived materials are well-suited for biomedical and environmental applications as they are inherently biocompatible, water-stable, capable of assembling under mild conditions, and readily interface with biological systems. These attributes contrast with many MOFs and COFs, which often require additional modifications to enhance biocompatibility^11^, are assembled under elevated temperatures or in organic solvents, and/or rely on intermolecular bonds that are susceptible to degradation in aqueous environments^12–16^.

Recently, peptide-based frameworks have emerged as a bioinspired class of porous materials. Peptides are ideal building blocks because they combine proteinogenic properties (*e.g*, folding, assembly, and binding) with the synthetic plasticity that is accessible via solid-phase peptide synthesis (SPPS) and the growing bioconjugation synthetic toolkit^17,18^. As a result, peptides have immense potential to create chemically rich protein-mimetic architectures with diverse properties and functions that extend beyond the proteome.

The conformational flexibility of peptides, and their limited tertiary structure, however, discourages their self-assembly into extended, porous architectures. To overcome this limitation, reported peptide frameworks have primarily been constructed from short peptides (*e.g*., di- and tripeptides) or have used oligoprolines and/or noncanonical amino acids to rigidify the building blocks^19–33^. While these strategies have yielded impressive porous architectures, including mesoporous frameworks^19,21,34^, and even the emergence of predictive design rules^21^, the relatively narrow peptide design space available for fabricating porous frameworks limits the full potential of this materials class. Expanding the peptide building block library represents a key element in broadening the architectural diversity of peptide-based frameworks, and ultimately, diversifying their potential applications. In our approach, disclosed here, we utilize lipidated collagen-mimetic peptides (CMPs) that fold into crystallizable collagen triple helices. The hierarchical collagen triple helix fold affords the structural rigidity for creating crystalline, porous frameworks. Furthermore, drawing from decades of research on CMPs that demonstrate their chemical and structural diversity^35–41^, CMP-based building blocks have the potential to further propel peptide frameworks from a niche materials class to a predictive and expansive assembly platform^21^, akin to MOFs and COFs. While recent efforts have ventured in this direction of using hierarchical, diverse building blocks (*e.g*., tetrameric coiled coil building blocks)^34,42–45^, ongoing efforts are needed to expand this assembly design space and broaden the application scope of peptide-based materials.

**Fig. 1:**
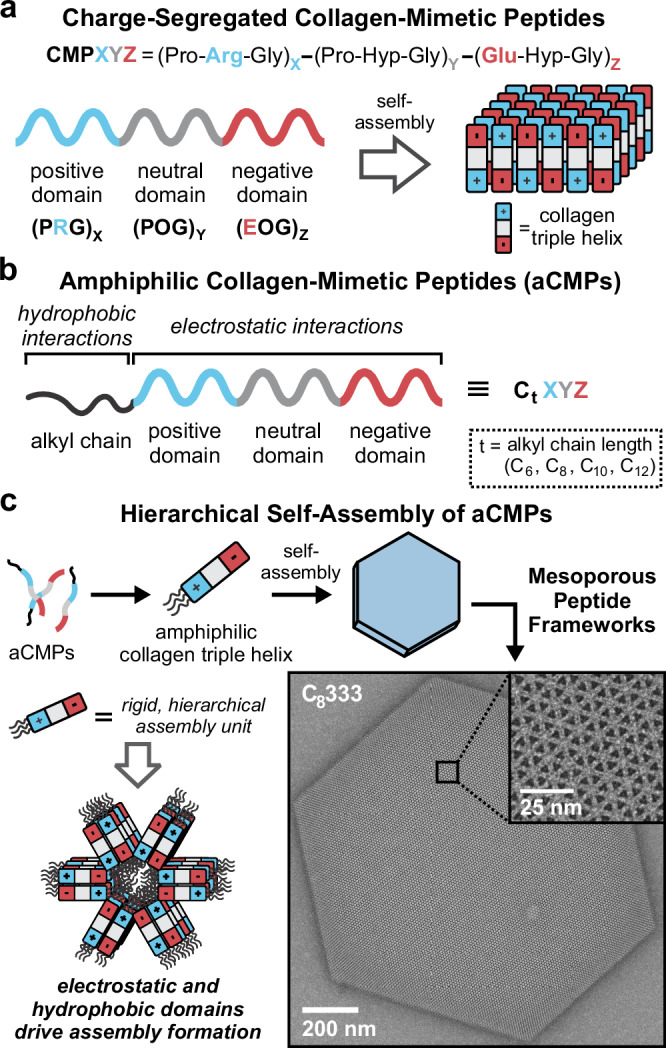
aCMP design and assembly. **a** CMPs comprise a triblock sequence architecture consisting of positive, neutral, and negatively charged domains. These charge-segregated CMPs assemble into a 2D array via antiparallel packing of collagen triple helices. **b** aCMPs comprise an alkyl chain attached to the N-terminus of charge-segregated CMPs. **c** aCMPs self-assemble hierarchically into porous frameworks comprising mesoporous channels via electrostatic and hydrophobic interactions.

Here, we disclose the construction of porous, crystalline frameworks that are assembled from amphiphilic collagen-mimetic peptides (aCMPs; Fig. 1). We demonstrate that aCMPs, which fold into crystallizable collagen triple helices, afford multidirectional, orthogonal intermolecular interactions that give rise to extended, porous frameworks with mesoporous channels. Mesoporous frameworks, defined by pore sizes between 2–50 nm, are of special interest as the size of the pores supports fast molecular diffusion and mass transfer, and the ability to accommodate large functional and biologically relevant guest species, (*e.g*., proteins, enzymes, and nanoparticles) throughout the entire assembly–all while retaining high access to the internal surface area^46^. We envision that this initial study will lay the foundation for employing aCMPs, and other rigid, helical peptide amphiphiles, as versatile, synthetically modular building blocks for the development of peptide-based assemblies.

## Results

### aCMPs assemble into mesoporous crystals

Prior work has shown that CMPs, in which positive, neutral, and negatively charged triads are segregated within the peptide sequence can serve as rigid, helical building subunits for the construction of D-periodic collagen fibers and 2D crystalline nanostructures^47–50^. These “triblock” CMPs are first heated to temperatures above the collagen triple helix melting temperature (T_m_) to ensure a homogeneous monomer population and then cooled to temperatures below the collagen triple helix T_m_, initiating the formation of triple helices that pack antiparallel into a 2D array via Coulombic interactions (Fig. 1a). Intrigued by the crystallizable properties of charge-segregated CMPs, we initially hypothesized that appending a hydrophobic aliphatic chain to their termini might create a frustrated system in which hydrophobic and electrostatic interactions compete for preferred packing arrangements, and that this competition, when finely tuned, could lead to the development of peptide-based architectures that utilize both types of interactions synergistically (Fig. 1b, c).

**Fig. 2:**
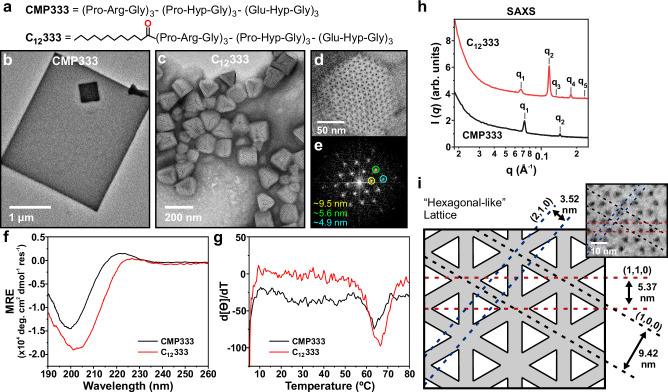
Characterization of CMP333 and C_12_333. **a** Sequences of **CMP333** and **C**_**12**_**333**. **b** Stained TEM image of CMP333 (1 mg/mL) nanosheets in 20 mM MES buffer after 1 week of assembly time. **c** Stained TEM image of **C**_**12**_**333** assemblies (0.5 mg/mL) after 1 week of assembly time. **d** Zoom-in image of pristine **C**_**12**_**333** crystal, which reveals porous triangular-shaped channels and (**e**) corresponding FFT analysis. **f** CD spectra of **CMP333** and **C**_**12**_**333** assemblies (1 mg/mL) after 1 week of assembly time. **g** First derivative of the CD signal at 224 nm as a function of temperature for **CMP333** and **C**_**12**_**333** assemblies (1 mg/mL). **h** SAXS scattering profile of **CMP333** and **C**_**12**_**333** assemblies (4 mg/mL) and corresponding *d*-spacing values associated with all identified peaks. **i** Assembly model of the hexagonal-like lattice associated with **C**_**12**_**333** frameworks (inset: zoomed-in of TEM image in panel **d**).

As a starting point, we chose **CMP333**, which consists of three Pro-Arg-Gly, Pro-Hyp-Gly, and Glu-Hyp-Gly triads, as our nonlipidated CMP (Fig. 2a). **CMP333** was synthesized using SPPS, purified via reverse-phase high-performance liquid chromatography (HPLC), and characterized using matrix-assisted laser desorption/ionization time-of-flight mass spectrometry (MALDI-TOF MS; Fig. S1). **CMP333**, across three different concentrations (0.5, 1, and 2 mg/mL) was dissolved in 20 mM MES (2-(N-morpholino)ethanesulfonic acid) buffer (pH 6.0), heated to 90 °C, and slow-cooled to room temperature. In line with previous reports^47–49^, transmission electron microscopy (TEM) images confirm the assembly of **CMP333**, at all concentrations, into multi-layer, micron-sized nanosheets (Fig. 2b and S2).

Interestingly, under identical assembly conditions, we observe a dramatic shift in assembly morphology for the lipidated derivative in which dodecanoic acid was conjugated to the N-termini of **CMP333** (Fig. 2a, c and S3). This aCMP, termed **C**_**12**_**333**, assembles into monodisperse, crystalline particles with distinguishable facets and an internal architecture consisting of triangular-shaped mesoporous channels (Fig. 2d), which stand in stark contrast to other reported structures assembled from lipidated CMPs without the charge-segregated sequence architecture^51–53^. Fast Fourier transform (FFT) of a pristine crystal, in which the pores are exposed perpendicular to the substrate, reveals hexagonal sets of Bragg spots that correspond to interplane distances of ~9.5, ~5.6, and ~4.9 nm (Fig. 2e). Dynamic light scattering (DLS) of the assembled **C**_**12**_**333** solutions, after 24 h and 1 week of assembly, confirms the monodispersity of **C**_**12**_**333** frameworks with sizes of ~150 nm (Fig. S4 and Table S1).

Circular dichroism (CD) spectroscopy of **CMP333** displays the characteristic profile for collagen triple helices with positive and negative maxima at ~224 and ~200 nm, respectively (Fig. 2f and S5). **C**_**12**_**333** displays a similar profile, however, the CD spectra are red-shifted and the positive maximum is attenuated in comparison to its nonlipidated counterpart (Fig. 2f and S6). Although these features suggest that **C**_**12**_**333** forms slightly perturbed triple helices, presumably due to aggregation of the hydrophobic alkyl chains which can disturb the preferred one-residue stagger that constitutes the canonical collagen triple helix fold^53^, we cannot rule out orientation and scattering effects associated with interrogating large CMP assemblies. Thermal denaturation studies confirm their cooperative unfolding with thermal transitions at ~62 °C for **CMP333** and between 66 and 74 °C for **C**_**12**_**333**, depending on aCMP concentration (Figs. 2g, S5, 6, and Table S2). The increased T_m_ of **C**_**12**_**333** suggests that, although lipidation may slightly distort the triple helical structure, the stability of the resulting distorted structures is enhanced^54,55^. In addition to CD, we synthesized and assembled a scrambled derivative, **C**_**12**_**333**_**Scr**_ (Fig. S7a–c), which preserves the charge separation, but lacks the Gly repeat at every third position – a strict requirement for forming collagen triple helices. Under identical assembly conditions, **C**_**12**_**333**_**Scr**_ does not assemble into crystalline porous frameworks (Fig. S7d). This result provides additional support that collagen triple helices undergird the formation of aCMP frameworks.

Small- and wide-angle X-ray scattering (SAXS/WAXS) was employed to characterize the internal structure of **CMP333** and **C**_**12**_**333** assemblies (4 mg/mL). The scattering profile of **CMP333** reveals four Bragg peaks at *q* values of 0.072, 0.145, 0.424, and 0.604, which correspond to *d*-spacings of 8.69, 4.34, 1.48, and 1.04 nm, respectively (Fig. 2h, S8, Table 1, and Table S3). These values, which represent global average lattice parameters of crystals in their native, hydrated state, differ slightly from the values obtained from FFT analysis of the crystal depicted in Fig. 2e. The minor discrepancy in *d*-spacings likely stems from multiple sources including projection effects, uranyl acetate staining, and drying effects. The first peak, corresponding to a *d*-spacing of 8.69 nm, is close to the contour length of **CMP333** (~8.3 nm; Figure S9) and is attributed to repeat stacking distances associated with multiple CMP layers within CMP nanosheets^47^. The second peak, which corresponds to a *d*-spacing that is half the distance of the first peak (4.34 nm), corresponds to repeat distances between the (002) planes^47^. The latter peaks found in the WAXS profile (Fig. S8b), with *d*-spacings of 1.48 and 1.04 nm, correspond to repeat distances between the (100) and (110) planes, respectively, and indicate that the triple helices are packed in a slightly distorted tetragonal lattice (1.04 x √2 ≈ 1.48; Fig. S10). These *d*-spacings are consistent with previously reported multilayer CMP nanosheets, in which collagen triple helices pack into tetragonal/distorted tetragonal lattices via antiparallel packing of upright collagen triple helices (Fig. 1a)^47–49^.

**Table 1:** Calculated *d*-spacings (nm) of Bragg peaks obtained from SAXS (peak labels shown in Figs. 2h, 4a, and S8b)

	*d*-spacing (nm)				
Peptide	q_1_	q_2_	q_3_	q_4_	q_5_
**CMP333**	8.69	4.34	1.48	1.04	-
**C**_**12**_**333**	9.42	5.37	4.81	3.52	2.29
**C**_**10**_**333**	9.17	5.29	3.45	-	-
**C**_**8**_**333**	8.14	4.70	4.08	3.08	-

In contrast to **CMP333**, we observe several Bragg peaks in the SAXS profile for **C**_**12**_**333** assemblies that correspond to *d*-spacings of 9.42, 5.37, 4.81*, 3.52, and 2.69 nm, respectively (Fig. 2h, S8c, d, Table 1, and Table S3; * denotes that, although a faint peak can be observed, the peak for q_3_ is close to noise level). These Bragg reflections are closely related in that they exhibit a peak position ratio of 1:$\sqrt{3}$:2:$\sqrt{7}$:$\sqrt{12}$ which confirms the underlying “hexagonal-like” lattice structure. The “hexagonal-like” nomenclature acknowledges the slight deviation from a perfect hexagonal lattice. The three most intense peaks, correlating to *d*-spacings of 9.42, 5.37, and 3.52 nm (q_1_, q_2_, and q_4_, respectively) correspond to the (100), (110), and (210) planes (Fig. 2i). These *d*-spacings (q_1_ and q_2_) closely mirror the interplane distances obtained from the FFT analysis (Fig. 2e). In addition, we carried out temperature-dependent SAXS measurements to probe the assembly formation. **C**_**12**_**333** was heated to 90 °C and scattering profiles were collected after each 10 °C drop in solution temperature (Fig. S11). Interestingly, Bragg peaks were first observed at 70 °C, which is just below the melting transition temperature of **C**_**12**_**333** at similar aCMP concentrations (Fig. S6d, e). These results imply that crystal formation coincides with formation of collagen triple helices, which is consistent with prior studies that employed nonlipidated CMPs^49^.

### Effect of varying alkyl chain length

We hypothesized that shortening the alkyl chain length would reduce the hydrophobic driving force and may lead to the formation of nanosheets via electrostatic interactions, akin to **CMP333**. Using our established protocols, we synthesized and purified three additional aCMP derivatives in which decanoic acid, octanoic acid, and hexanoic acid were attached to the N-terminus of **CMP333** to yield **C**_**10**_**333,** **C**_**8**_**333**, and **C**_**6**_**333**, respectively (Fig. S12). All aCMPs (0.5, 1, and 2 mg/mL) were dissolved in 20 mM MES buffer (pH 6.0), heated to 90 °C and cooled to room temperature.

**Fig. 3:**
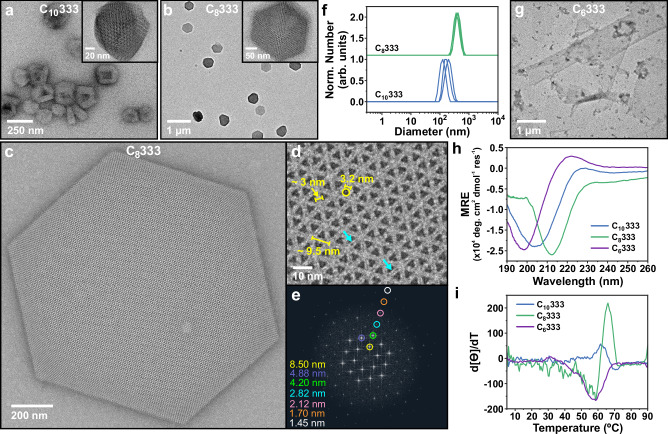
Characterization of C_10_333, C_8_333, and C_6_333. Stained TEM image of (**a**) **C**_**10**_**333** (1 mg/mL) and (**b**) **C**_**8**_**333** (1 mg/mL) after 1 week of assembly. Note: inset images show assemblies formed at 0.5 mg/mL. **c** Stained TEM image of a thin, planar crystal assembled from **C**_**8**_**333** (1 mg/mL). These crystals represent a minor assembly product. **d** Zoomed-in image of the thin, planar crystal reveals highly ordered arrangement of hexagonal mesoporous channels. Various distance measurements, including pore size and thickness, are shown. Blue arrows highlight the side-by-side striations observed within pore walls. **e** FFT analysis of the thin, planar crystal shown in (**c**) reveals several Bragg spots. Corresponding *d*-spacings are shown. **f** DLS size distributions of **C**_**10**_**333** and **C**_**8**_**333** assemblies (1 mg/mL) after 1 week of assembly. **g** Stained TEM image of **C**_**6**_**333** (2 mg/mL) after 1 week of assembly, revealing the formation of ill-defined aggregates and nanosheets. **h** CD spectra of **C**_**10**_**333,** **C**_**8**_**333**, and **C**_**6**_**333** assemblies (1 mg/mL) after 1 week of assembly. **i** First derivative of the CD signal at 224 nm as a function of temperature for **C**_**10**_**333,** **C**_**8**_**333**, and **C**_**6**_**333** assemblies (1 mg/mL) after 1 week of assembly time.

Analogous to **C**_**12**_**333,** **C**_**10**_**333** and **C**_**8**_**333** assemble into porous crystalline frameworks that comprise triangular-shaped mesoporous channels (Fig. 3a–d and S13, 14). For **C**_**8**_**333** crystals we observe two distinct populations with hexagonal morphologies. Although most **C**_**8**_**333** frameworks are multi-faceted with 6 distinct faces and “disc-like” (Fig. 3b and S14), a small population of large (> 1 μm), thin crystals is also observed (Fig. 3c and S15). For the multi-faceted crystals, only a small area affords a top-down view of the mesoporous channels (Fig. 3b, inset and S14), whereas the large, planar crystals are different in that they appear to be thin, flat, and the hexagonally arranged pores are observed throughout the crystal face (Fig. 3c, d and S15). Detailed analysis of the lattice, within the thin, planar crystals, reveals pore dimensions of 3.2 ± 0.2 nm and wall thicknesses of 2.9 ± 0.3 nm (Fig. 3d and S16). The latter is consistent with the width of two CMP triple helices, and close inspection reveals two visible striations within many of the walls, suggesting that the walls comprise CMP triple helices that are packed side-by-side (Fig. 3d). FFT analysis of the planar framework confirms the high degree of crystallinity with Bragg spots that correspond to interplane distances as low as ~14 Å (Fig. 3e). To test the stability of **C**_**8**_**333** crystals in other solvents, pre-assembled **C**_**8**_**333** frameworks, originally in MES buffer (pH 6.0), were exchanged in pure water and phosphate buffered saline (PBS; pH 7.4). While **C**_**8**_**333** assemblies were stable in pure water, we observed degradation of **C**_**8**_**333** assemblies in PBS buffer, likely due to shielding of the inter-triple helix electrostatic interactions that maintain the structural integrity of the assembled architecture (Fig. S17). Future efforts involve improving the stability of aCMP frameworks at physiological ionic strength (an important consideration for their biomedical applications) via post-assembly modification.

DLS analysis reveals that the average particle size increases as a function of alkyl chain length (173 ± 2 nm and 380 ± 2 nm for 1 mg/mL solutions of **C**_**10**_**333** and **C**_**8**_**333** frameworks, respectively; Fig. 3f and S18, 19, and Table S1). We attribute the increase in particle size to the fewer number of nucleation events that occur for aCMPs with shorter alkyl tail lengths due to the decreased hydrophobic driving force. This result mirrors prior reports which demonstrate that faster triple helix formation, via increasing the Pro-Hyp-Gly block, leads to greater number of nucleation events and thus, smaller size distributions^48^.

At all concentrations studied (0.5-2 mg/mL), **C**_**6**_**333** predominantly forms ill-defined aggregates, however, at higher concentrations (2 mg/mL) sheet-like assemblies are observed (Fig. 3g and S20). The emergence of nanosheets, akin to those observed from the assembly of non-lipidated, charge-segregated CMPs^47–49^, including **CMP333**, agrees with our hypothesis that shortening the hydrophobic alkyl chains allows electrostatic interactions to dictate the final assembly morphology and drive the packing of aCMP triple helices into 2D nanostructures. These results imply that **C**_**6**_**333** is close to the “tipping point” where electrostatic interactions begin to dominate the self-assembly process. Future studies aim to explore the ability to create dynamic architectures that can toggle between both assembly products. Lastly, in an attempt to decouple the lipid-driven and CMP-driven interactions, octanoic acid was mixed with non-lipidated **CMP333** (1:1 molar ratio; ~ 1 mg/mL **CMP333**) and the mixture was heated and cooled using the same assembly protocol described above. TEM images confirm the absence of porous frameworks, indicating that the amphiphilic nature of aCMPs, in which lipid and CMP domains are covalently linked, is necessary for directing the assembly of aCMPs into porous frameworks (Fig. S21). Interestingly, the decoupling of aCMPs yields spherical particles with clearly defined concentric layers (Fig. S21).

CD spectra of **C**_**10**_**333** and **C**_**8**_**333** display similar profiles to **C**_**12**_**333**, but with greater attenuation of the positive signal at ~224 nm and further red-shifting of the CD curve (Fig. 3h and S22, 23). The greater distortion of the collagen triple helix CD signature is likely due to the larger particle sizes of **C**_**10**_**333** and **C**_**8**_**333** assemblies (Table S1), which augment scattering-related artifacts. In contrast, CD spectra of **C**_**6**_**333** closely align with the CD results of **CMP333**, indicating the presence of unperturbed collagen triple helices (Fig. 3h and S24). Thermal denaturation curves, which monitor the CD intensity at 224 nm, reveal an increase in triple helical character for **C**_**10**_**333** and **C**_**8**_**333** assemblies at elevated temperatures immediately prior to unfolding (Fig. 3i and S22, 23). These results, which have been observed with previously studied lipidated CMPs^53^, demonstrate the thermally induced restructuring of the collagen triple helix fold, presumably due to more favorable interchain alignment as a result of the melting of alkyl chains within the hydrophobic cores.

**Fig. 4:**
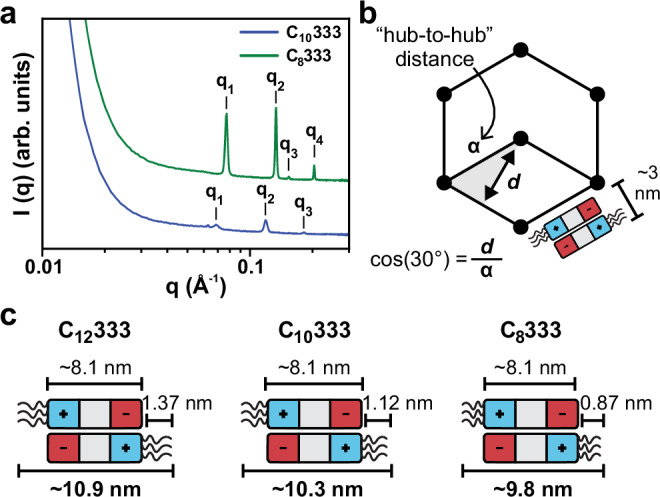
Structural characterization of aCMP frameworks. **a** SAXS scattering profile of **C**_**10**_**333** and **C**_**8**_**333** and corresponding *d*-spacing values for each of the labeled peaks. **b** α values, which refer to the distance between hubs, were calculated for **C**_**12**_**333** (10.9 nm), **C**_**10**_**333** (10.6 nm), and **C**_**8**_**333** (9.4 nm) frameworks, based on SAXS data (*d* = q_1_ values). **c** Contour lengths of a pair of aCMP triple helices that are packed antiparallel.

SAXS/WAXS analysis of **C**_**10**_**333** and **C**_**8**_**333** frameworks (4 mg/mL) confirm their internal hexagonal lattice architecture (Fig. 4a, S25, Table 1, and Table S3). In comparison to **C**_**12**_**333** frameworks, Bragg peaks are shifted to larger *q* values, *i.e*., smaller *d*-spacings, which indicate that the lattice parameters of these frameworks are slightly contracted (Fig. 4a, Table 1, and Table S3). Using *d*-spacings that correspond to q_1_, we calculated hub-to-hub distances (α), *i.e*., distances between nodes, of approximately 10.6 and 9.4 nm for **C**_**10**_**333** and **C**_**8**_**333**, respectively, which are shorter than the α value calculated for **C**_**12**_**333** (10.9 nm; Fig. 4b). We initially reasoned that the varying alkyl chain lengths of aCMPs account for these differences as these values are on par with the contour lengths of two antiparallel-packed aCMPs, assuming a rise/residue of ~0.3 nm (0.3 nm × 27 res. = 8.1 nm), which is slightly longer than the rise/per residue for collagen triple helices (0.286 nm/res.; Fig. 4c). However, molecular modeling and simulation experiments (see MD data below) establish that the alkyl chains are too short to bridge the hub diameter due to steric constraints associated with the triple helix fold. We speculate that the lattice contraction, as a function of tail length, may be attributed to subtle packing differences of aCMP triple helices. More tightly packed triple helices may promote smaller hubs, which give rise to smaller lattice parameters, however, additional experiments are needed to validate this hypothesis.

SAXS/WAXS analysis of **C**_**6**_**333** nanosheets confirms that their underlying structure is consistent with **CMP333** and previously reported CMP nanosheets that are also assembled from non-lipidated, charge-segregated CMPs (Fig. S25e, f and Table S3)^47–49^. Bragg peaks corresponding to *d*-spacings of 1.02 and 1.44 nm, which closely matches Bragg peaks obtained for **CMP333**, confirm the underlying tetragonal lattice (1.02 × $\sqrt{2}$ = 1.44) that comprises their assembly. We note that the SAXS/WAXS profile reveals no peak associated with the length of the aCMP (peak “q_1_” in Fig. 2h), which we attribute to their single-layer construction.

### Probing the lipid environment within aCMP frameworks

To probe the lipid environment within the hydrophobic “hubs,” fluorescence experiments using Prodan, a solvatochromic membrane probe^56,57^, were carried out. Prodan has two emission bands: one centered at ~530 nm and one ranging from 420 nm to 480 nm, depending on the degree of packing within the lipophilic environment. The first band is associated with the emission of Prodan in an aqueous environment, whereas the position of the second band is used to determine the characteristics of the lipid membrane. A blueshift of the second peak to 420 nm indicates the presence of a gel-like phase within the frameworks, in which the lipid tails are tightly packed (*i.e*., more crystalline-like). On the other hand, peaks positioned closer to 480 nm are associated with the presence of  a liquid crystalline phase in which the lipid tails are more loosely packed (*i.e*., more liquid-like). To better quantify the degree of packing, Parasassi *et al*. developed an experimental framework for characterizing lipid membranes from liquid crystal to gel phases using a three-wavelength general polarization parameter (3wGP)^56^. A higher 3wGP value is associated with a gel-like phase consisting of highly ordered lipid chains.

**Fig. 5:**
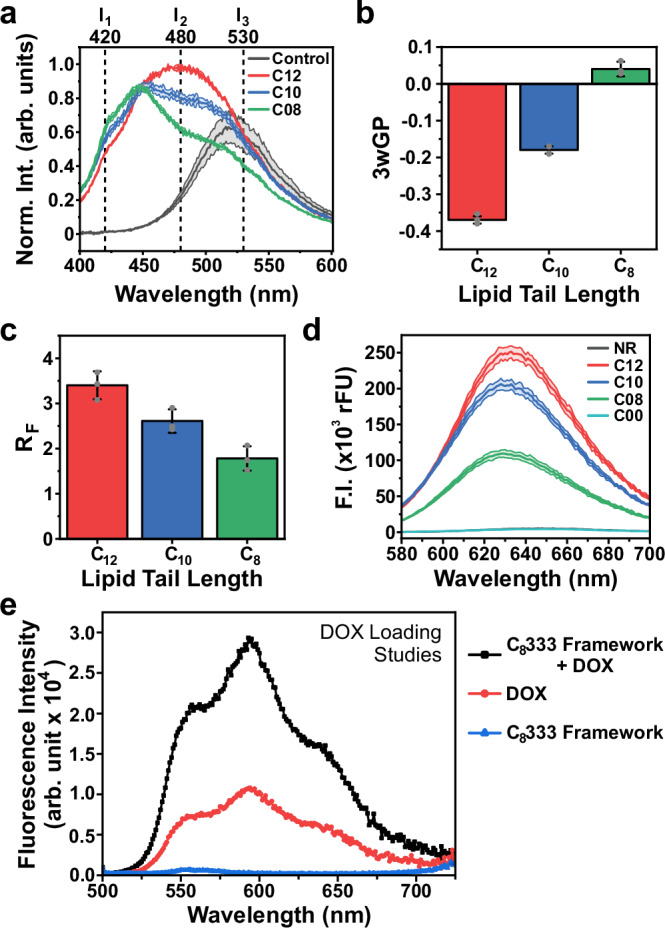
Characterization of lipid domain. **a** Fluorescence emission spectra of solutions comprising Prodan mixed with **C**_**12**_**333** (“C12”), **C**_**10**_**333** (“C10”), and **C**_**8**_**333** (“C08”) assemblies. **b** 3wGP values for **C**_**12**_**333,** **C**_**10**_**333**, and **C**_**8**_**333** assemblies (std. deviation, SD, shown). **c** Partition coefficients, R_f_, for **C**_**12**_**333,** **C**_**10**_**333**, and **C**_**8**_**333** assemblies (SD shown). **d** Fluorescence emission spectra for **C**_**12**_**333,** **C**_**10**_**333,** **C**_**8**_**333**, and **CMP333** (labeled “C00”) assemblies in the presence of Nile Red. Control experiment (“labeled NR”) shows fluorescence emission spectra of Nile Red in 20 mM MES buffer (pH 6.0). **e** Fluorescence emission spectra of **C**_**8**_**333** frameworks with and without DOX, and only DOX in 20 mM MES buffer (pH 6.0).

Prodan was added to solutions containing **C**_**12**_**333,** **C**_**10**_**333**, and **C**_**8**_**333** frameworks in 20 mM MES buffer (pH 6.0). Each solution was excited at 350 nm and the emission spectra were recorded from 400 to 600 nm (Fig. 5a). We observe a shift in the fluorescence emission wavelength from 480 to 420 nm as a function of decreasing lipid tail length (Fig. 5a), which implies that the packing of the lipid chains within hydrophobic environments of the hubs become more gel-like as the lipid tail length decreases. These results are further reflected in the 3wGP values in which a large negative value was calculated for **C**_**12**_**333**, and increasing values were obtained for **C**_**10**_**333** and **C**_**8**_**333** (Fig. 5b). The Prodan emission spectra also allows one to determine partition coefficients (R_f_), which describes the relative partitioning of Prodan between the hydrophobic and aqueous environments. Consistent with our results, a clear trend is observed in R_f_ values where **C**_**12**_**333** > **C**_**10**_**333** > **C**_**8**_**333**. This confirms that the more liquid-like C_12_ environment within **C**_**12**_**333** frameworks allows more Prodan to partition into the hydrophobic core than the more densely packed, crystalline-like environment of **C**_**8**_**333**^56^.

In addition to Prodan, Nile Red was also employed to probe the uptake of hydrophobic molecules within aCMP frameworks. Nile Red, which has virtually no emission in aqueous environments, emits strongly when located within lipophilic environments^58^. Fresh 1 mg/mL samples of aCMP frameworks were prepared, allowed to mature for 1 week, and then diluted 2× with 6 μM Nile Red in buffer (20 mM MES; pH 6). The samples were excited at 540 nm and their emission spectra were recorded from 580 to 700 nm. Emission spectra reveal an increase in fluorescence emission for aCMPs frameworks assembled from aCMPs with longer alky chains (**C**_**12**_**333** > **C**_**10**_**333** > **C**_**8**_**333**; Fig. 5d), which corroborates the results obtained from the Prodan experiments. We note that no fluorescence was observed when Nile Red was added to **CMP333** assemblies or in the absence of peptide (Fig. 5d).

The successful uptake of hydrophobic molecules within hydrophobic domains of aCMP frameworks prompted us to examine the potential of aCMP frameworks for encapsulating therapeutic cargo. **C**_**8**_**333** frameworks (10 μM aCMP concentration) were incubated in the presence of doxorubicin (DOX), an anti-cancer drug at various DOX:aCMP molar ratios (Fig. 5e and S26). At low DOX:aCMP molar ratios (1:10), we observed a 174% increase in fluorescence signal at 593 nm compared to the emission of a solution containing the same DOX concentration without assembly (Fig. 5e and S26g). The increase in fluorescence is attributed to the partitioning of DOX into the lipophilic environments within aCMP frameworks, which shields DOX from collisional quenching with water, as observed in previous reports^59–61^. We note that as DOX loading increases, we observe a relative decrease in fluorescence compared to the DOX-only control, which is likely due to self-quenching as more DOX is loaded within confined hydrophobic domains (Fig. S26). TEM images confirm that the aCMP frameworks remain stable after incubation with DOX (Figure S27). Altogether, these fluorescence studies demonstrate that aCMPs comprise lipophilic domains that are capable of encapsulating hydrophobic small molecules, including biologically relevant cargo (DOX). Moreover, these results highlight the increased crystallinity of the hydrophobic environment for porous frameworks that are assembled from aCMPs with shorter alkyl chain lengths (*e.g*., **C**_**8**_**333**).

### Cryo-TEM analysis of C_8_333 frameworks

**Fig. 6:**
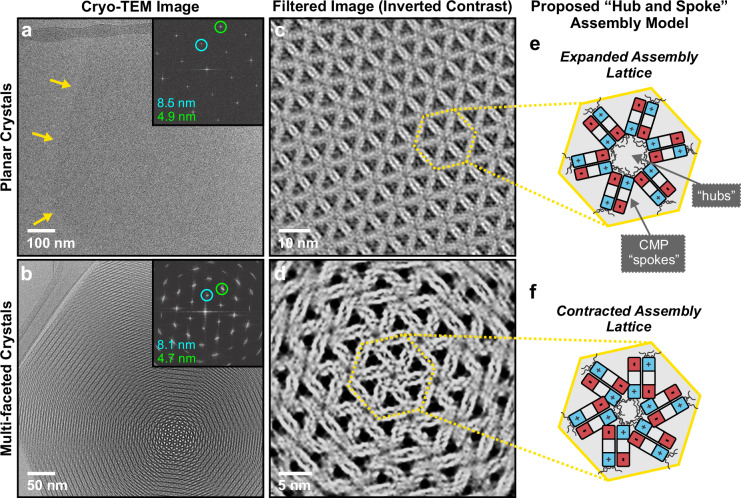
Cryo-TEM analysis of C_8_333 frameworks. High-resolution cryo-TEM image and corresponding FFT analysis (insets) of (**a**) thin, planar **C**_**8**_**333** framework (arrows indicate crystal edges) and (**b**) multi-faceted **C**_**8**_**333** framework. Inverse FFT analysis after Wiener and low-pass filter were applied to the FFT for the (**c**) thin, planar **C**_**8**_**333** framework and (**d**) multi-faceted **C**_**8**_**333** framework. Proposed “hub-and-spoke” assembly model that highlights the discrepancy in packing of aCMPs, which leads to varying degrees of contraction within the lattice parameters for the (**e**) planar **C**_**8**_**333** framework and the (**f**) multi-faceted **C**_**8**_**333** framework. CMP domains, having collagen triple helical character, serve as bridges that connect hexagonally arranged “hubs” into an ordered hexagonal lattice.

Cryogenic transmission electron microscopy (cryo-TEM) was employed to provide greater insight into the underlying structure of aCMP frameworks. We focused our efforts on **C**_**8**_**333** assemblies, as these frameworks give rise to highly ordered crystals with two distinct populations. Both assembly architectures (thin, planar crystals and multi-faceted crystals) were captured via cryo-TEM (Fig. 6a, b). FFT analysis of the planar crystalline framework reveals a high degree of internal order (Fig. S28). The first two sets of Bragg spots, which correspond to *d*-spacings of ~8.5 and ~4.9 nm (Fig. 6a, inset), match the *d*-spacings obtained from FFT analysis of the stained **C**_**8**_**333** framework collected via TEM (Fig. 3e).

In contrast, FFT analysis of the multi-faceted crystal, in which six distinct crystal faces are observable, displays Bragg spots that correspond to *d*-spacings of ~8.1 and ~4.7 nm (Fig. 6b, inset, S28). These interplane distances align with *d*-spacings obtained from SAXS (Fig. 4a) and indicate that the internal lattice dimensions of these crystals, which constitute the major morphological product, are contracted compared to their planar crystalline counterparts. Moreover, while SAXS measurements are azimuthally averaged, the smearing of Bragg peaks observed for the multi-faceted crystal indicates that the orientation of the hexagonal lattice is rotated to some degree along the *z*-axis (Fig. 6b, inset). TEM images of several **C**_**8**_**333** crystals, including some that were assembled under slower cooling rates (see “Methods” for details), provide evidence that these crystals assemble into elongated hexagonal bipyramids that are slightly gyrated (Fig. S29).

Inverse FFT analysis, in which the original FFT was processed using Wiener and low-pass filters (2 ~ 3 Å) affords greater resolution of the assembly lattice (Fig. 6c, d)^62^. For the thin, planar crystal, the filtered FFT analysis confirms the presence of two parallel striations that connect the hexagonally arranged hubs (Fig. 6c), which corroborates the features observed from the stained **C**_**8**_**333** framework (Fig. 3d). Inspection of the multi-faceted crystal, however, reveals slight discrepancies. The linear features that bridge the hexagonally arranged hubs are slightly offset, creating a twisted lattice packed framework, which allows for closer packing of the hubs (Fig. 6d). We attribute this twisted packing to the global twisting that is observed for these crystalline assemblies (Fig. S29). Furthermore, for both **C**_**8**_**333** crystal types, a decrease in contrast can be observed within the center of the hubs (Fig. 6c, d), which suggests that the hubs are solvent-accessible (see MD data below).

### Proposed assembly model

We propose a “hub-and-spoke” assembly model that accounts for the structural information gathered on aCMP frameworks (Fig. 6e, f). In this proposed model, which serves as our current working hypothesis, CMP domains serve as “spokes” that radiate in six directions from solvent-accessible “hubs,” where lipid domains associate via the hydrophobic effect. We propose that the spokes comprise two antiparallel-packed triple helices (TEM and cryo-EM micrographs reveal two parallel striations; Fig. 3d and Fig. 6c). For **C**_**8**_**333** frameworks, there are two slightly different variations of the hub-and-spoke assembly model. We propose that for thin, planar **C**_**8**_**333** crystals, which exhibit an expanded lattice as evidenced by cryo-TEM (Fig. 6a, c), the hubs are less compacted and can more easily accommodate CMP domains without sterically induced twisting. This allows some **C**_**8**_**333** crystals to adopt a thin, hexagonal prism morphology (Fig. 3c and 6a). On the other hand, for the multi-faceted crystals, we propose that significant contraction of the assembly lattice prevents the in-plane arrangement of CMP domains. Consequently, this creates a twisted assembly unit that propagates to form an elongated hexagonal bipyramidal crystal habit that is slightly rotated perpendicular to the pyramidal face (Fig. 6f and S29). Unlike the expanded lattice, the perturbation within the assembly unit for the multi-faceted crystals hinders their lateral growth.

### MD simulations of proposed assembly packing model

To test the plausibility of the proposed assembly packing model, we employed all-atom molecular dynamics (MD) simulations. Simulations were carried out at 300 K using OpenMM software package^63^ and CHARMM-c36m force field^64^. Initial simulations attempted to shed light on the assembly of aCMPs from individual **C**_**12**_**333** monomers and prearranged **C**_**12**_**333** triple helices. However, for both sets of simulations, in which 12, 24, or 36 **C**_**12**_**333** copies were simulated, no organized structures were observed after 500 ns of simulation (Fig. S30). Contact maps, which reveal interchain interactions between residues within aCMPs, display an increased number of contacts along the diagonal axis (from bottom left to top right) for the simulation conducted on prearranged **C**_**12**_**333** triple helices, with the greatest number of contacts occurring for residues within the central triads (Fig. S31). These contacts along the diagonal axis, which serve as a proxy for collagen triple helix formation, indicate that the initial triple helix structure is maintained throughout the simulation. No triple helices are observed after 500 ns for simulations carried out on free **C**_**12**_**333** monomers (Figs. S30, 31).

**Fig. 7:**
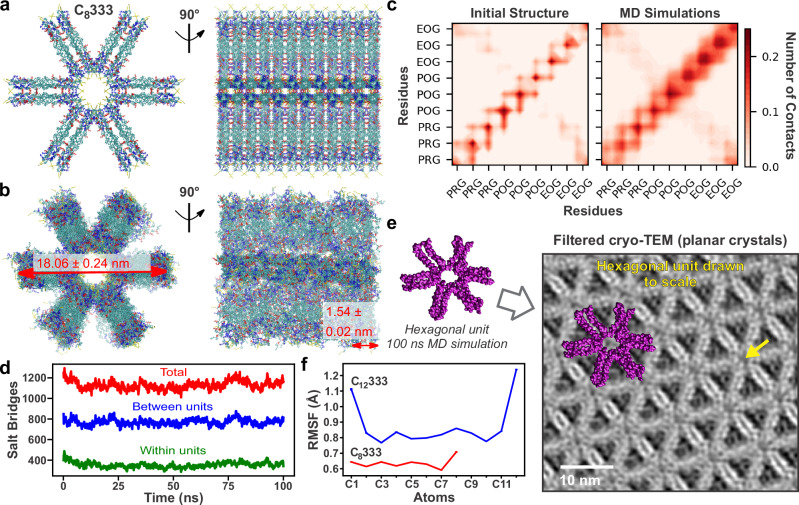
MD Simulation of Proposed Hub-and-Spoke Assembly Model. **a** Initial MD simulation trajectory of hub-and-spoke assembly packing model for **C**_**8**_**333**. The assembly system comprises 12 hub-and-spoke models (hexagonal units) that are constructed from 12 **C**_**8**_**333** triple helices. **b** Final MD simulation trajectories of the proposed assembly packing model after 100 ns of simulation. **c** Contact maps for individual residues within aCMPs. Contacts along the diagonal axis highlight intra- and inter-triple helical interactions within collagen triple helices and between antiparallel-packed triple helices, respectively. **d** Number of salt bridge contacts within a single hexagonal unit (351.00 ± 22.74), between hexagonal units (769.20 ± 28.56), and for all units within the assembly (1126.73 ± 39.56), throughout the duration of the simulation (SD values listed). **e** Overlay of hexagonal, hub-and-spoke unit after 100 ns MD simulation onto filtered cryo-TEM image from Fig. 6c. The hexagonal unit is drawn to scale with the cryo-TEM micrograph. The yellow arrow highlights decreased contrast within the core of the hubs. **f** RMSF values of acyl chain carbon atoms for **C**_**8**_**333** and **C**_**12**_**333** assemblies.

Following these initial simulations, we explored the use of MD simulations to test the stability and dynamics of the proposed hub-and-spoke assembly model (Fig. 6e). MD simulations were first carried out on **C**_**8**_**333** assemblies. Twelve copies of the hub-and-spoke hexagonal units were arranged along the x-axis with 16.5 Å interval spacings (Fig. 7a). Following minimization and preparation steps (see “Methods” section), a production run of 100 ns was carried out. Inspection of the final trajectory of the MD simulation confirms that the hub-and-spoke assembly model remains intact (Fig. 7b). Root mean square deviation (RMSD) analysis, in which the C_α_ of each hexagonal unit is superimposed with their starting position, reveals structural deviations between the starting and final trajectories, and displays good convergence after 100 ns for each hexagonal unit (Fig. S32). Analysis of the contact maps, both before and after the simulation runs, highlights the collagen triple helix fold that is maintained throughout the simulation, as determined by the significant number of contacts along the diagonal axis (Fig. 7c). Greater number of contacts are observed for the EOG triads at the C-terminus compared to the PRG triads at the N-terminus. These results suggest that lipidation may slightly perturb the collagen triple helix fold, at least for the N-terminal third of aCMP triple helices. Dihedral angles of approximately −60° and 150° for φ and ψ, respectively, further confirm the presence of collagen triple helices, as these values closely match the expected dihedral angles associated with the collagen triple helix fold (Fig. S33). Additionally, contact maps unveil inter-triple helix interactions, which are indicated by the significant number of contacts along the opposite diagonal axis (*i.e*., from top left to bottom right). These interactions arise from attractive forces (*i.e*., salt bridges) between PRG and EOG triads that are facilitated by the antiparallel packing of aCMP triple helices (Fig. 7c). MD analysis of the trajectories reveals the extensive number of salt bridges between Arg and Glu residues within the proposed assembly model, showing excellent convergence after 100 ns of simulation (Fig. 7d). Over the last 75 ns of simulation, on average, a total of 1126.73 ± 39.56 salt bridges is present within the proposed assembly model. This average can be divided between the number of salt bridges between and within hexagonal units (769.20 ± 28.56 and 351.00 ± 22.74 salt bridges, respectively). Back calculation from the total average number of salt bridges yields, on average, ~7.83 salt bridges per triple helix, which represents roughly half of the total number of Arg and Glu residues within a single triple helix (9 Glu + 9 Arg = 18 potential salt bridge contacts). As expected, from the proposed assembly model, for every one intra-unit salt bridge, each triple helix has approximately two inter-unit salt bridges (Fig. 7d).

MD analysis, of the last 75 ns, reveals an average end-to-end distance (not including lipid tails) of 18.06 (std. error = 0.24 nm) and interunit stacking distance of 1.54 (std. error = 0.02 nm; Fig. 7b). The former is consistent with the SAXS and cryo-TEM data. As shown in Fig. 7e, there is excellent agreement when overlaying a single simulated proposed assembly unit onto the filtered, high-resolution cryo-TEM image of the **C**_**8**_**333** framework from Fig. 6c. Furthermore, the solvent-accessible channels within hubs, as determined from MD simulations, are congruent with the decreased contrast that are observed within the interior of the hubs (yellow arrow in Fig. 7e).

To compare differences between **C**_**8**_**333** and **C**_**12**_**333**, we carried out analogous MD simulations for **C**_**12**_**333** proposed assembly models. Like the **C**_**8**_**333** experiment, MD simulations confirm the structural integrity of the hub-and-spoke packing model, and that a majority of the hexagonal units show convergence after 100 ns of simulation (Fig. S34 and S35). MD analysis of contact maps, RMSD plots, dihedral angles, and number of salt bridges for **C**_**12**_**333** assemblies are similar to **C**_**8**_**333**, albeit with some slight discrepancies (Fig. S34–36). Inspection of the post-simulation contact map implies that the internal structures of **C**_**12**_**333** triple helices are less ordered compared to their **C**_**8**_**333** counterparts (Fig. S34c). Furthermore, a plot of the root mean square fluctuation (RMSF) of the carbon atoms within the lipid chain, which provides information on their average mobility, illustrates that the **C**_**12**_**333** lipid tail is significantly more mobile than that of **C**_**8**_**333** (Fig. 7f). These results corroborate the fluorescent studies (see above), which disclose the more liquid-like lipophilic domains of **C**_**12**_**333** frameworks compared to the more ordered, crystalline-like lipophilic domains of **C**_**8**_**333** frameworks (Fig. 5a, b). The greater internal order of both the hydrophobic and CMP domains of **C**_**8**_**333** compared to **C**_**12**_**333** may contribute to the more contracted lattice of the former, as the greater degree of order affords the triple helical spokes to pack more closely together. However, as mentioned above, more experiments are needed to validate this claim. While the average end-to-end distance of the **C**_**12**_**333** packing model is slightly larger than the **C**_**8**_**333** packing model (18.14 nm and 18.06 nm for **C**_**12**_**333** and **C**_**8**_**333** assemblies, respectively; Fig. 7b and **S34b**), these values are well within standard errors of one another and therefore MD simulations, at the moment, cannot recapitulate the change in lattice parameters as a function of aCMP tail length.

## Discussion

In the past, peptide-based materials have primarily been relegated to lower dimensional architectures with relatively limited architectural diversity. These restrictions limit the utility of peptide-based materials for a large subset of desirable applications. This work, among others, represents a shift towards the development of higher dimensional peptide-based materials through the use of hierarchical peptide building block designs. We reveal that, unlike prior reports of peptide amphiphiles^65^, aCMPs assemble into extended, mesoporous peptide frameworks due, in part, to the crystallizable propensity of collagen triple helices that are driven by interhelical electrostatic interactions^47–49,66–68^. Furthermore, we demonstrate that these peptide frameworks possess accessible domains/channels that are able to encapsulate and bind guest molecules, including doxorubicin, a therapeutic anti-cancer drug.

Building on our proposed working assembly model, current efforts are underway to establish assembly design rules for systematically manipulating pore dimensions and pore chemistries within aCMP frameworks. Given the ability to modulate the aCMP sequence design by adjusting the alkyl chain length and CMP domain, we envision that aCMP frameworks may serve as a robust, highly tunable, assembly platform for the fabrication of mesoporous materials for a broad range of applications in sensing, separations, and encapsulation. While this work represents an important first step in the development of aCMP frameworks, additional efforts demonstrating the ability to systematically and rationally tune the physical and chemical features of aCMP frameworks are needed to evaluate the full utility of this materials assembly platform.

## Methods

### General methods

All chemical reagents were purchased from Sigma Aldrich Chemical Co. (St. Louis, MO) or Thermo Fisher Scientific Inc. (Waltham, MA) unless otherwise stated. Fmoc amino acids, Oxyma Pure, and resins were acquired from CEM (Matthews, NC). Matrix-assisted laser desorption ionization time-of-flight (MALDI-TOF) mass spectrometry data were collected using a Bruker Microflex LRF mass spectrometer (positive reflector mode) and using α-cyano-4-hydroxycinnamic acid (CHCA) as the ionization matrix (1:1 sample to matrix ratio). Lyophilized peptides were quantified gravimetrically, reconstituted in water or water-acetonitrile mixture, aliquoted, and lyophilized.

### Peptide synthesis and purification

Peptides were prepared using a CEM Liberty Blue microwave-assisted automated peptide synthesizer. Standard Fmoc SPPS was employed with iterative deprotection and coupling cycles based on 4-methylpiperidine (4MP) in dimethylformamide (DMF; 20% v/v) and DIC/Oxyma Pure-mediated activation, respectively. C_12_333, C_10_333, C_8_333, and C_6_333 were prepared on-resin (using a CEM pre-loaded Gly-Wang resin) by double-coupling dodecanoic acid, decanoic acid, octanoic acid, and hexanoic acid (0.2 M in DMF), respectively, to the N-termini of CMP333. Crude peptides were cleaved from resin for 3 hrs at room temperature with a cleavage mixture composed of 92.5% trifluoroacetic acid (TFA), 2.5% triisopropylsilane (TIPS), 2.5% 3,6-dioxa-1,8-octanedithiol (DODT), and 2.5% H_2_O. The peptides were precipitated with cold (5 °C) diethyl ether and centrifuged at 4500 rpm for 15 min at 5 °C. After centrifugation, the supernatants were discarded, and the pellets were dried under vacuum overnight. All peptides were purified via reverse-phase HPLC using an Agilent 1260 Infinity II prep-scale HPLC instrument, equipped with a Kinetex XB-C_18_ column (250 × 30 mm, 5 μm, 100 Å). Peptides were eluted with a linear gradient of acetonitrile-water with 0.1% TFA. Target fractions containing the desired peptide were collected, rotovaped, and lyophilized. The lyophilized peptides were resuspended in HPLC-grade water and dialyzed using a 2.5KDa MWCO cassette against pure water overnight. The next day the dialyzed peptide solutions were lyophilized. After lyophilization, the peptides were quantified gravimetrically, resuspended in HPLC-grade water, aliquoted, lyophilized, and stored at −30 °C. A yield of 11.1% (31.5 mg) was obtained for **C**_**12**_**333** at 0.1 mmol scale. For the synthesis of **C**_**10**_**333,** **C**_**8**_**333**, and **C**_**6**_**333**, the resin was roughly divided equally (0.025 mmol scale) and estimated yields of 16% (10.9 mg), 19% (12.9 mg), and 13% (9.05 mg) were obtained for **C**_**10**_**333,** **C**_**8**_**333**, and **C**_**6**_**333**, respectively.

### aCMP assembly preparation

Lyophilized peptides were dissolved in 20 mM MES buffer (pH 6.0). The solutions were vortexed and sonicated, transferred to 0.2 mL domed-capped microcentrifuge tubes, and placed in a Biorad T100 thermocycler. The solutions were heated to 90 °C for 5 min, then cooled (0.5 °C/2.5 min) to 20 °C. Once the samples were cooled to 20 °C, the solutions were transferred to a 1.5 mL centrifuge tube, sealed with parafilm, and left on the benchtop at room temperature. Extra-slow cooling experiments were conducted using a cooling rate of 0.5°/30 min.

### Circular dichroism (CD) spectroscopy

CD measurements were collected on a Jasco J-1500 spectropolarimeter using a 0.02 cm path length for spectra and a 0.1 cm path length for thermal denaturation studies. Three accumulations were recorded and averaged from 260 nm to 190 nm at a scanning rate of 100 nm/min with a bandwidth of 2 nm. Thermal denaturation experiments were performed by heating from 5 °C to 80 °C or 90 °C at a rate of 40 °C/hr. The intensity of the signal at 224 nm was recorded as a function of temperature, and the melting temperature (T_m_) was determined by plotting the first derivative. Mean residue ellipticity (MRE) was plotted as a function of wavelength or temperature by converting the raw ellipticity (mdeg) to MRE using equation (1):

1 $${MRE}=\frac{100\times \theta }{{cnl}}$$

where *θ* is the ellipticity (mdeg), *c* is the peptide concentration (mM), *n* is the number of residues, and *l* is the pathlength (cm).

### Transmission electron microscopy (TEM)

TEM images were collected with a Talos F200C G2 transmission electron microscope at an accelerating voltage of 200 kV. TEM specimens were prepared by mixing 2.5 μL of aCMP solutions with 2.5 μL of a 1% uranyl acetate solution directly on a 200-mesh carbon-coated copper grid. The mixed solutions were allowed to stand for 30 sec. After 30 sec, the excess liquid was wicked away with a piece of filter paper and the grids were allowed air dry for at least 1 hr. Images were analyzed using ImageJ analysis software.

### Dynamic light scattering (DLS)

DLS data were collected on a Malvern Zetasizer Pro. Size measurements were conducted in triplicate with 40 µL of samples, using disposable cuvettes, after 120 seconds of equilibration at 20 °C.

### Small- and wide-angle x-ray scattering (SAXS/WAXS)

SAXS/WAXS measurements were collected on a custom-built high-brilliance laboratory beamline for small and wide-angle X-ray scattering (SAXS/WAXS) at the BioPACIFIC Materials Innovation Platform at UCSB. The instrument is constructed using a high-brightness liquid Ga (9.24KeV) X-ray source (D2 + 70 kV from Excillum), a low background scatterless slit beam collimation system developed in-house, and a 4-megapixel hybrid photon counting area detector (Eiger2 R 4 M from Dectris) housed inside a 3 meter-long vacuum vessel. All samples were run free-standing, in the transmission geometry, and were exposed to X-ray radiation for 30 minutes. The resulting two-dimensional (2D) scattering images were then azimuthally averaged and converted to 1D scattering profiles using custom in-house written C code. SAXS measurements were measured on aCMP assemblies (4 mg/mL) after one week of assembly.

### Fluorescence spectroscopy

Fluorescence emission spectra were collected on a ClarioStar BMG plate reader using a 96-well plate. aCMP assemblies were prepared in 20 mM MES buffer (pH 6.0) at 1 mg/mL and allowed to incubate for 1 week. 250 µL of the aCMP assembly solutions were mixed with 250 µL of Nile Red solution (6 µM in methanol/MES buffer) to give final concentrations of 0.5 mg/mL and 3 µM for aCMP assemblies and Nile Red, respectively. A control solution of 3 µM Nile Red in MES buffer (pH 6.0) was also prepared. Each solution was transferred (150 µL) to three individual wells, excited at 540 nm, and the emission spectra were collected from 580 to 700 nm. For the Prodan experiments, 250 μL of aCMP assembly solutions were combined with 250 μL of 1 μM Prodan solution (in 20 mM MES, pH 6.0), and distributed to three wells with 150 μL of sample. Calculation of the 3-wavelength general polarization (3wGP) and partition coefficients (R_f_) was determined using the methods outlined by Parasassi et al*.*^56^. and the following equations:

2 $$3{wGP}=\frac{{R}_{12}-1}{{R}_{12}+1}$$

3 $${R}_{12}=\frac{{I}_{1}\times {K}_{32}}{{I}_{2}\times {K}_{32}-{I}_{3}+{I}_{3}\times {R}_{31}}$$

4 $${K}_{32}=\frac{{I}_{3w}}{{I}_{2w}}\,$$

5 $${R}_{31}=\frac{{I}_{3M}}{{I}_{1M}}\,$$

6 $${R}_{f}=\frac{{I}_{2}\times {K}_{32}-{I}_{3}}{{I}_{3}-{I}_{2}{R}_{32}}$$

where I_1_, I_2_, and I_3_ are the intensities at 420, 480 and 530 nm which corresponds to the Prodan emission in the gel phase, liquid crystalline phase and aqueous phase, respectively. Intensities (I) with a *w* subscript refer to spectra of Prodan in buffer (20 mM MES; pH 6.0), and intensities (I) with a subscript *M* refer to the sample spectra where the Prodan in buffer spectra was subtracted.

For DOX (doxorubicin hydrochloride) encapsulation experiments within **C**_**8**_**333** frameworks, all samples were equilibrated for 3 h. After equilibration, the solutions were excited at 483 nm and the emission spectra were recorded over a range of 500 to 725 nm at 30 °C. **C**_**8**_**333** concentrations were maintained at 10 μM while the DOX concentrations were adjusted from 1 to 20 μM to achieve solutions comprising DOX:aCMP molar ratios of 0.1, 0.2, 0.4, 0.6, 0.8, 1.0, and 2.0. The final volume in each well was 100 μL. In parallel, a control study was conducted using DOX in 20 mM MES buffer (pH 6.0) at 1, 2, 4, 6, 8, 10, and 20 μM in the absence of **C**_**8**_**333** assemblies.

### Cryogenic transmission electron microscopy (Cryo-TEM)

A 4 μL aliquot of sample solution was placed on a glow-discharged ultra-thin lacey carbon film 200 mesh gold grid (LC200-Au-UL, Electron Microscopy Sciences, Hatfield, PA, USA). The grid was plunge-frozen in liquid ethane at ~ 90% humidity and 8 °C using a Leica EM GP rapid-plunging device (Leica, Buffalo Grove, IL, USA) after blotting for 3.5 sec with filter paper. The flash-frozen grids were transferred into liquid nitrogen for cryo-EM imaging. The grid was imaged on a Titan Krios G3i TEM (Thermo Fisher Scientific) equipped with a Bio Quantum energy filter (Gatan Inc., Pleasanton, CA, USA) and operated at 300 keV. Micrographs were acquired using a Gatan K3 direct electron detector in correlated double sampling (CDS) mode^69^ and super-resolution mode, controlled by SerialEM^70^. The micrographs were acquired at a nominal magnification of 81 k× (1.05 Å/pixel) or 42 k× (2.10 Å/pixel). The total dose was ~50 e−/Å^2^. Motion correction of the multi-frame images was conducted by MotionCor2^71^. The Contrast Transfer Function (CTF) was determined, and phase flipped using the GCTF software package^72^. Then, the micrographs were analyzed by fast Fourier Transform (FFT) and processed by Wiener filter^62^ and low-pass filter (2 ~ 3 Å) in Gatan Digital Micrograph software.

### Molecular dynamics (MD) simulation

#### System preparation

We prepared the initial structures of **C**_**8**_**333** and **C**_**12**_**333** using CHARMM c36^73^ IC tables. The IC table of the lipidated proline residue was prepared by combining the IC table of proline for the amino acid coordinates and the IC table of glycine myristate, after converting it to laurate, for the lipid acyl chain. The triple helix was prepared first using CCBuilder 2.0^74^ for a peptide with proline at the N-terminal, and then N-terminal prolines in the triple helix were replaced with proline laurates and proline caprylates for **C**_**12**_**333** and **C**_**8**_**333** peptides, respectively. We prepared two sets of concentrated systems of **C**_**12**_**333**, in which the peptides are in the form of a monomer (referred to as “single peptide”) and in the form of a triple helix (referred to as “triple helix”) at each corresponding set. For each set, we prepared three systems with 12, 24, and 36 copies of peptides in a 150 Å cubic box, which correspond to peptide concentrations of 6, 12, and 18 mM. The initial structures of the protein mixtures were prepared by randomly distributing the monomers or triple helices. For this, we added the first peptide by translating its center of mass to a random position within a box with an edge size of 100 Å. Then, we added additional peptides by randomly positioning their center of mass and maintaining a distance of less than 5 Å between any carbon atoms of any two peptides. For the systems with triple helices, we added random rotations as well to fit the center of masses of trimers within a 90 Å cubic box without having any clashes within any trimers. The systems were solvated in a 150 Å cubic box and neutralized by adding one Na^+^ ion per peptide.

In addition, we prepared multi-hexagonal systems formed by triple helices of **C**_**8**_**333** and **C**_**12**_**333**. To assemble the initial model, first, we generated a hexagonal assembly unit by rotating and translating the anti-parallel triple-helix pairs in the y-z plane. Then, we performed energy minimization of the generated assembly unit using 50 steps of the steepest descent (SD) algorithm and 1000 steps of the adopted basis Newton-Raphson (ABNR) algorithm. Energy minimization was performed in an implicit water environment using a generalized Born implicit water model, the analytical model called generalized Born molecular volume II (GBMV II) in the CHARMM simulation package^75^. Then, we translated the units along the *x*-axis to allow 16.5 Å spacing between the center of masses of neighboring assembly units of a total of 12 units. We performed energy minimization on the final assembly system using 50 steps of the SD and 100 steps of ABNR at the implicit water environment using GBMV II. The minimized systems were then solvated in 196.4 Å and 198.9 Å cubic boxes for **C**_**8**_**333** and **C**_**12**_**333** models, respectively. Peptides were neutralized by capping the C-terminus with -NH-CH3 (CT3 patch in CHARMM C36 topology file). All the systems were prepared using the Python programming language, CHARMM software^76^ and the MMTSB package^77^.

#### MD simulations

Single peptide and triple helix concentrated systems were energy minimized for 5000 steps and equilibrated for 1.6 ns. Equilibration was performed by increasing the temperature from 100 to 300 K at 50 K increments at every 0.12 ns for the first 0.6 ns using a 1 fs time step. Then, the time step was increased to 2 fs for 0.5 ns and to 4 fs for 0.5 ns. Constraints with force constants of 400 and 40 kJ/mol/nm^2^ for the heavy atoms of the backbones and side chains of the proteins were applied for the entire equilibration. After equilibration, we performed 500 ns of MD simulation for each system. Periodic boundary conditions were applied. The Particle Mesh algorithm was used to calculate long-range electrostatic interactions^78^. Lennard-Jones interactions were switched between 10 and 12 Å. Bonds with H atoms were constrained using the SHAKE algorithm. We used CHARMM c36m^64^ parameters for proteins. For water molecules, we used the CHARMM modified TIP3P water model^79^. We used a 4 fs time step for the production simulation. To prevent any instabilities that a 4 fs time step may cause, we repartitioned atomic masses to increase the masses of H atoms to 3 a.m.u. as suggested earlier^80^.

The multi-hexagonal assembly systems were energy minimized for 5000 steps and equilibrated for 6.2 ns. The temperature was increased from 100 to 300 K during the first 1.2 ns of the equilibration with a time step of 1 fs. For the last 5 ns of the equilibration, a time step of 2 fs was used. Constraints with force constants of 400 and 40 kJ/mol/nm^2^ for the backbones and side chains of the peptides were applied for the first 2.7 ns and then slowly reduced to 10 kJ/mol/nm^2^ for both backbone and side chain for the remaining 3.5 ns of the equilibration. After the equilibration, the production run was performed for 100 ns using the same simulation parameters used for the concentrated systems, except for the time step, which was 2 fs for the assembly system. All the simulations were performed using the OpenMM software^63^ on GPU machines using Python scripts generated by the CHARMM-GUI server^81,82^.

For single peptide and triple helix simulations, we analyzed the contact maps for inter-peptide contacts. Contact maps were generated by calculating the minimum heavy atom distances between each residue for a pair of segments and assigning it to a contact if the distance is within 5 Å. Then the contacts were normalized with the number of pairs and the number of frames. For each frame, we calculated only intermolecular contacts and added only the pairs that are within contact.

For the multi-hexagonal assembly system, we analyzed root mean square deviations (RMSDs), contact maps, dihedral angles, numbers of salt bridges, end-to-end and stacking distances, and root mean square fluctuations (RMSFs). RMSD values of C_α_ atoms were calculated for each hexagonal unit separately after superimposing the C_α_ positions in the trajectory to the initial structure. We calculated the contact map by first calculating the contact maps for each hexagonal unit separately and then averaging over the number of units. Dihedral angles were calculated for all the amino acids except the N- and C-termini. The standard errors were calculated for 12 hexagonal units. The number of salt bridges was calculated between the oxygen and nitrogen atoms of the side chains of glutamic acid and arginine, respectively. We used 4 Å as a cutoff and counted any oxygen-nitrogen pair within the cutoff as a salt bridge. End-to-end distances were calculated from the C_α_ atom for the residues at the edges (either N- or C-termini) for each pair of anti-parallel triple helices that have a 180° angle for each hexagonal unit. Unit stacking distances were calculated as distances of center of masses of C_α_ atoms for each neighboring hexagonal unit. RMSF values were calculated for carbon atoms of the lipid acyl chains for each peptide separately and then averaged over the peptides. For a single peptide, we first superimposed the carbon atoms of the lipid chain from each frame onto the initial structure, then we calculated the average structure of that peptide. We then superimposed the structures from each frame onto the calculated average structure. After that, we calculated the RMSF values of the lipid carbon atoms over the trajectory. Finally, we calculated the average RMSF values over the lipid chains of the total assembly model. All analyses were performed using the Python programming language and Scikit-learn^83^, NumPy^84^ and MDAnalysis^85^ libraries.

## Supplementary information

Supplementary Information

Transparent Peer Review file

## Data Availability

The data supporting the findings of this study are available in the Article and the Supplementary Information. Data is available from the corresponding author upon request.

## Code availability

All scripts for generating the initial structures and performing the analysis are provided in the GitHub repository (https://github.com/bercemd/aCMP).
