# Supplementary material for: Mesoporous peptide frameworks engineered from crystallizable collagen-mimetic peptide amphiphiles
Source: Nat Commun. 2026 May 15;17:6919. doi: 10.1038/s41467-026-73068-2 (PMC13388958; doi:10.1038/s41467-026-73068-2)
Supplement: Supplementary file 1 — Supplementary Information [file 41467_2026_73068_MOESM1_ESM.pdf]

Supplementary Information

Mesoporous peptide frameworks engineered from crystallizable collagen-mimetic peptide amphiphiles

Anthony R. Perez,[†] Jianfang Liu,[‡] S M Mobin Sikder,[†] Anjan Maity,[†] Adekunle Adewole,[†] Jacob Oakden,[⸸] Gang Ren,[‡] Bercem Dutagaci,[⸸,†] and Andrea D. Merg[†*]

[†]Department of Chemistry and Biochemistry, University of California, Merced, Merced, CA 95343

[⸸]Department of Molecular and Cell Biology, University of California, Merced, Merced, CA 95343

[†]Health Sciences Research Institute, University of California, Merced, Merced, CA 95343

[‡]The Molecular Foundry, Lawrence Berkeley National Laboratory, Berkeley, CA 94720

Supporting Figures

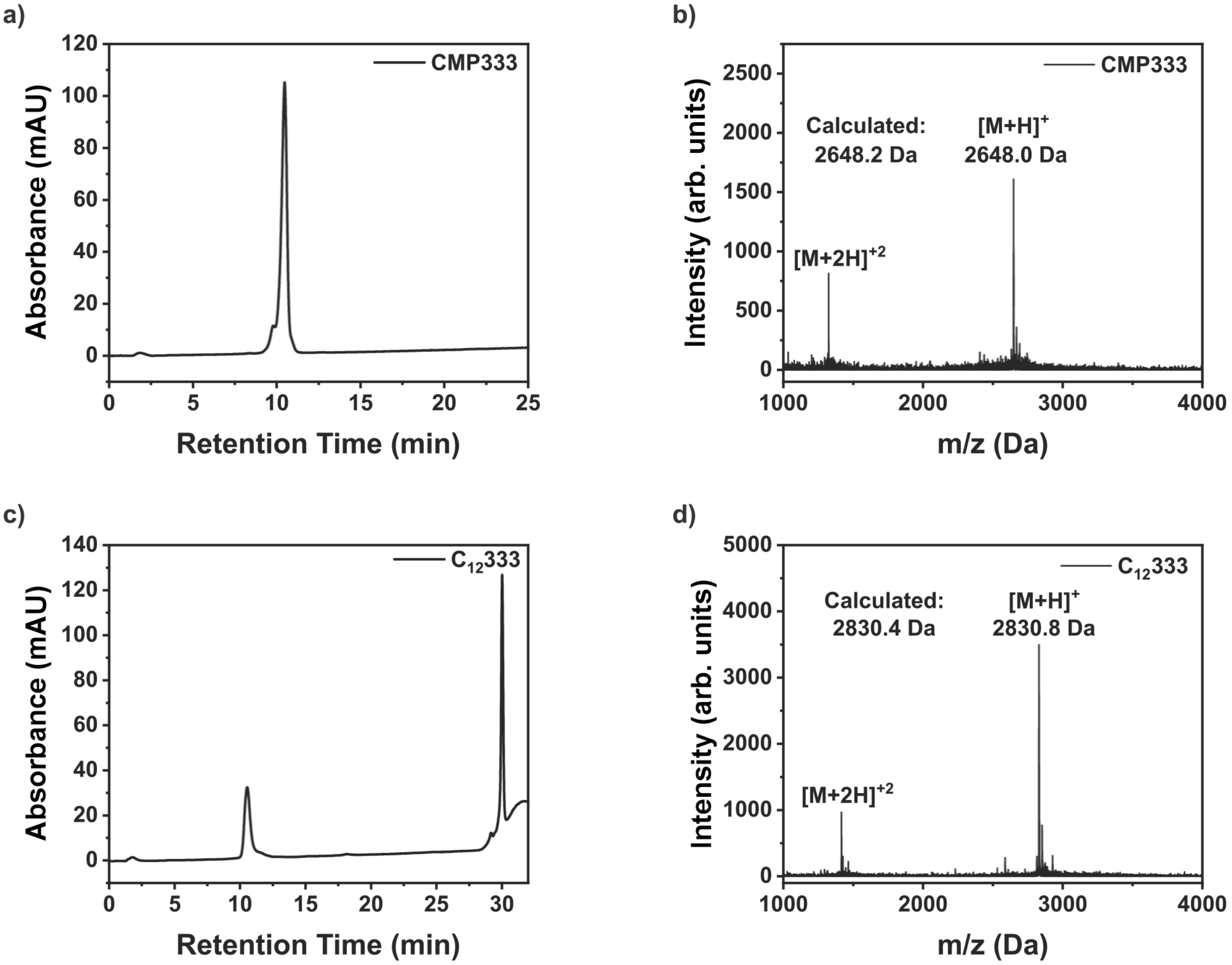

Figure S1. Analytical HPLC and MALDI-TOF MS spectra for (**a**,**b**) **CMP333** and (**c**, **d**) **C$_{12}$333**. Note: both peaks for **C$_{12}$333** displayed the target mass.

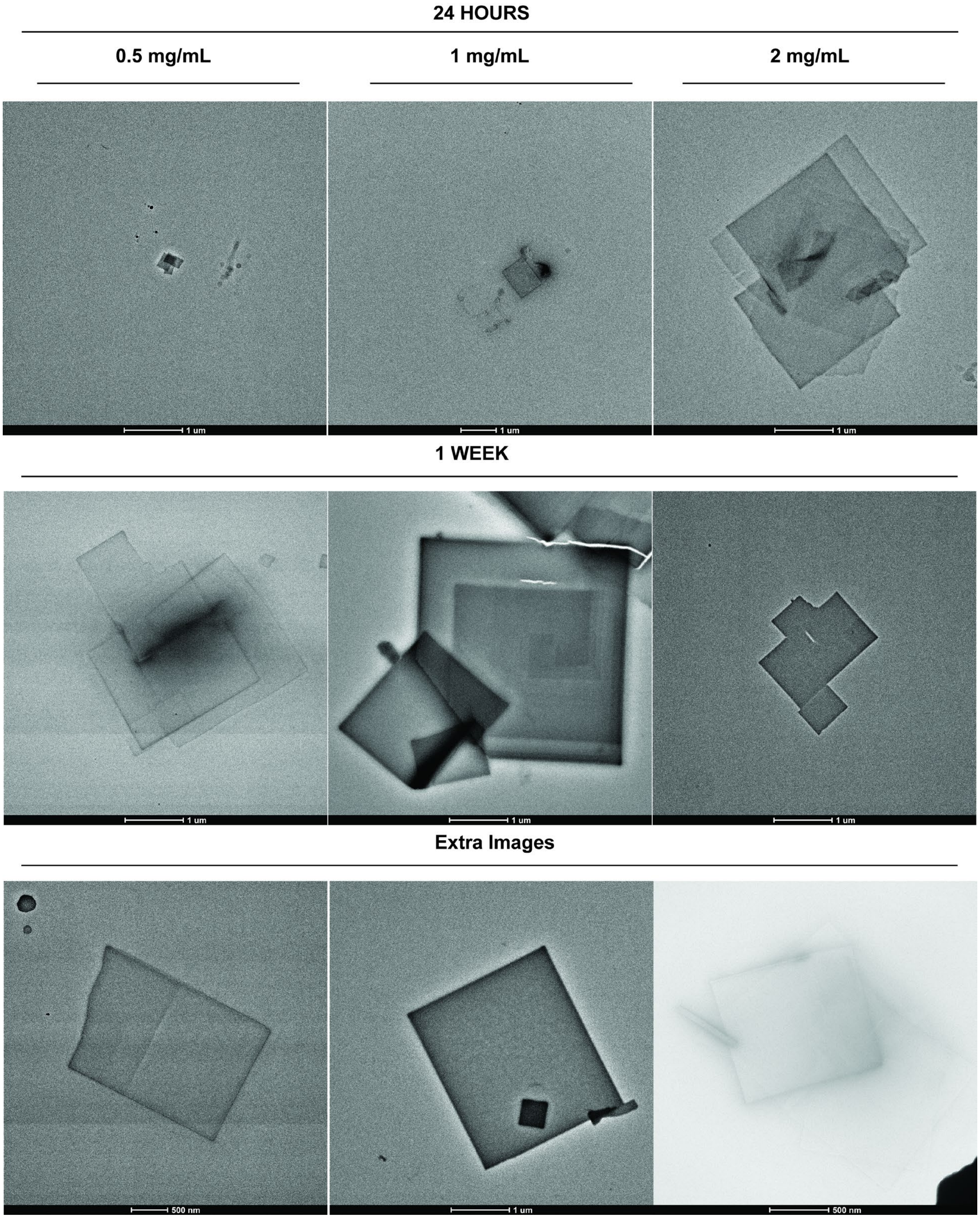

Figure S2. Stained TEM micrographs of **CMP333** at various CMP concentrations and assembly time points.

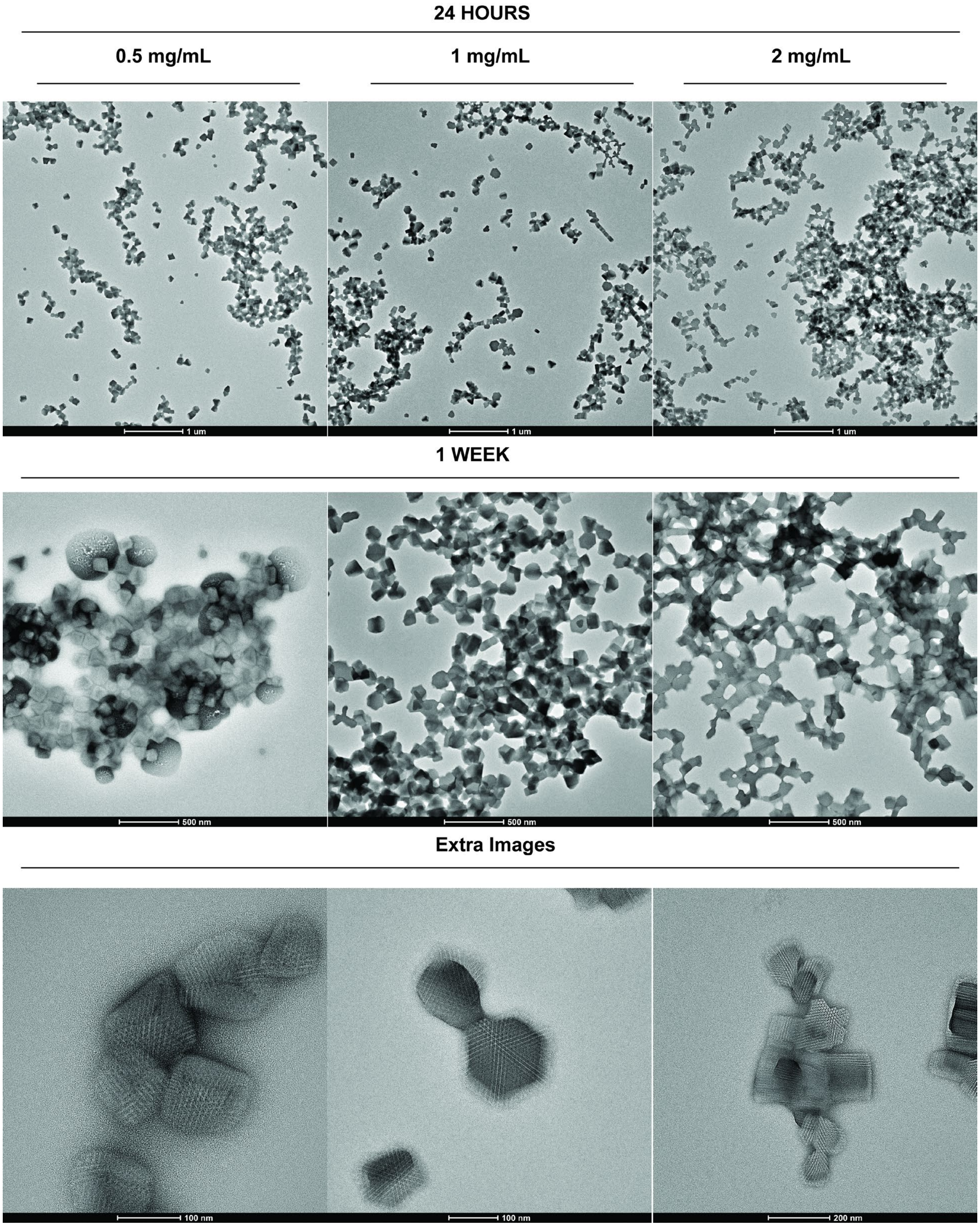

Figure S3. Stained TEM micrographs of **C$_{12}$333** at various aCMP concentrations and assembly time points.

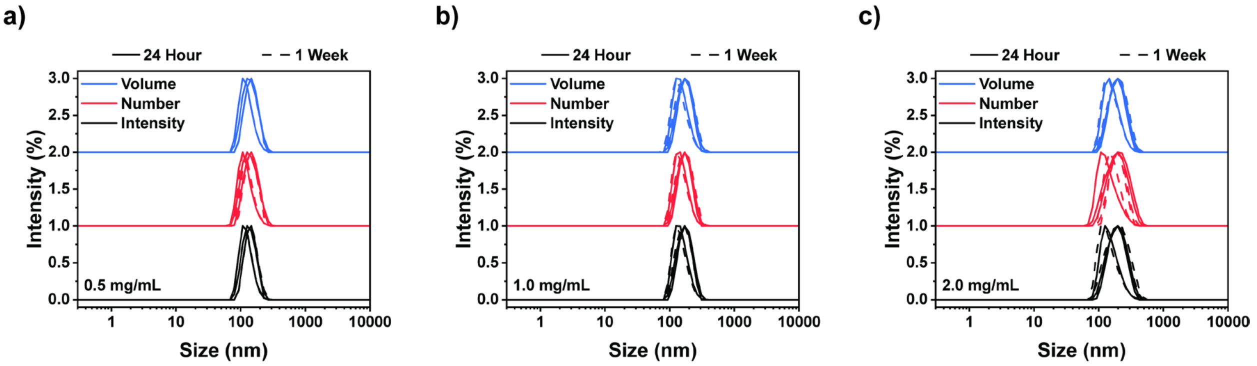

Figure S4. DLS profiles for **C12333** at (**a**) 0.5 mg/mL, (**b**) 1 mg/mL, and (**c**) 2 mg/mL. See **Table S1** for mean data values for each experiment.

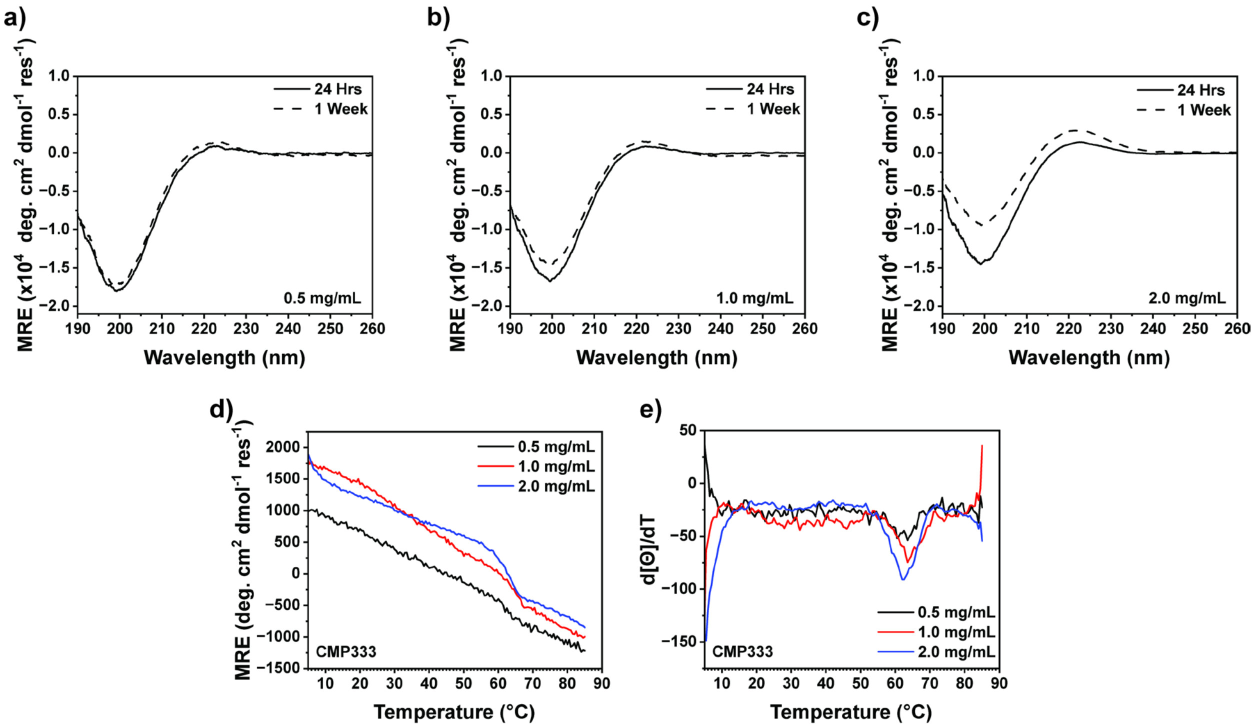

Figure S5. CD spectra of **CMP333** at (**a**) 0.5 mg/mL, (**b**) 1 mg/mL, and (**c**) 2 mg/mL. (**d**) CD thermal denaturation plots of **CMP333**. (**e**) First derivative of the CD signal at 224 nm as a function of temperature for **CMP333** at various concentrations.

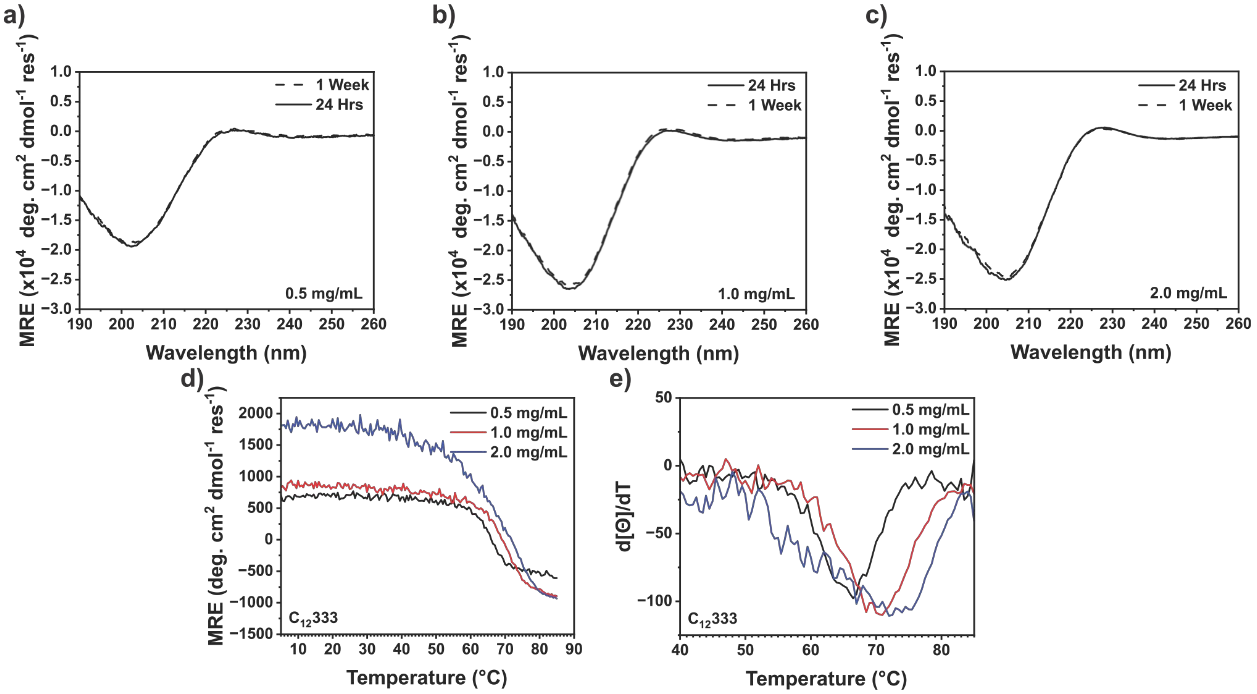

Figure S6. CD spectra of **C12333** at (**a**) 0.5 mg/mL, (**b**) 1 mg/mL, and (**c**) 2 mg/mL. (**d**) CD thermal denaturation plots of **C12333**. (**e**) First derivative of the CD signal at 224 nm as a function of temperature for **C12333** at various concentrations.

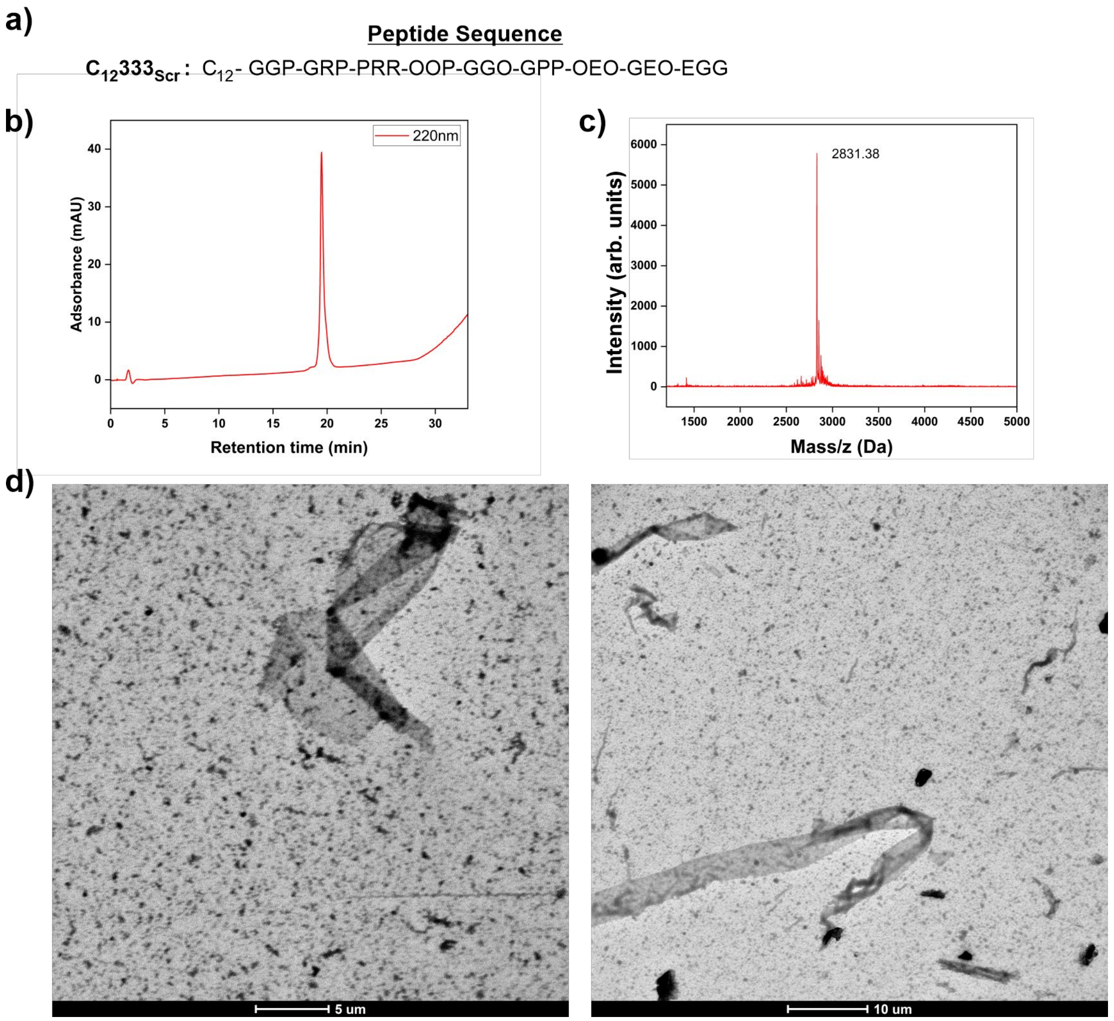

Figure S7. (**a**) Sequence of $\mathbf{C_{12}333_{Scr}}$. (**b**) Analytical HPLC and (**c**) MALDI-TOF MS spectra for $\mathbf{C_{12}333_{Scr}}$ (Theo MW: 2831.1 Da). (**d**) Stained TEM images of $\mathbf{C_{12}333_{Scr}}$ (1 mg/mL) in 20 mM MES buffer (pH 6.0) after 7 days.

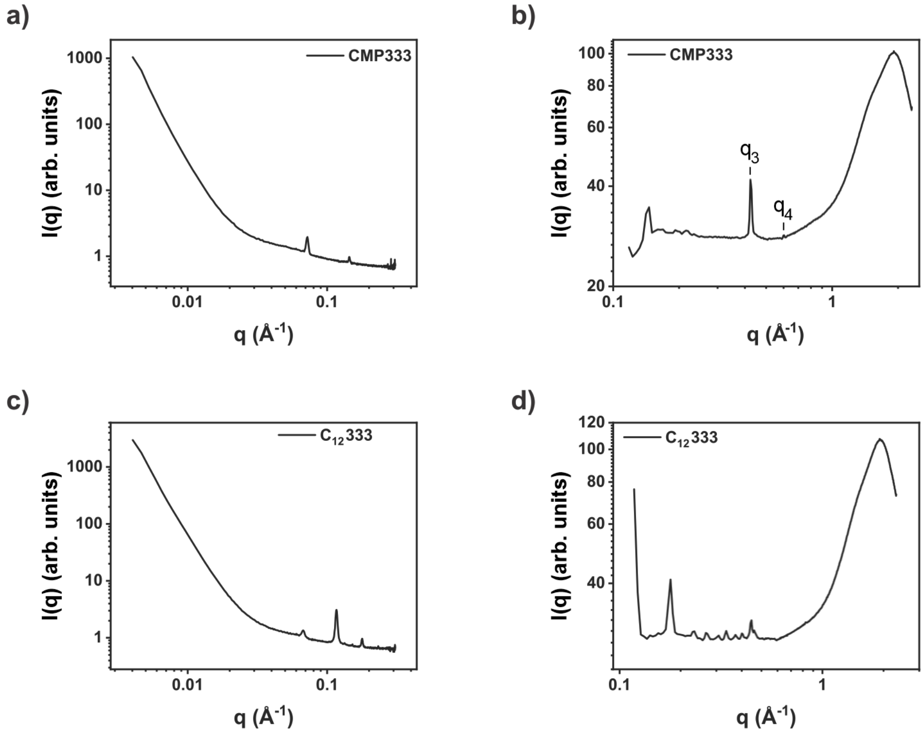

Figure S8. SAXS/WAXS scattering profiles for (**a**,**b**) **CMP333** and (**c**,**d**)and **C$_{12}$333**. See **Table S3** for tabulated q-values and corresponding *d*-spacings.

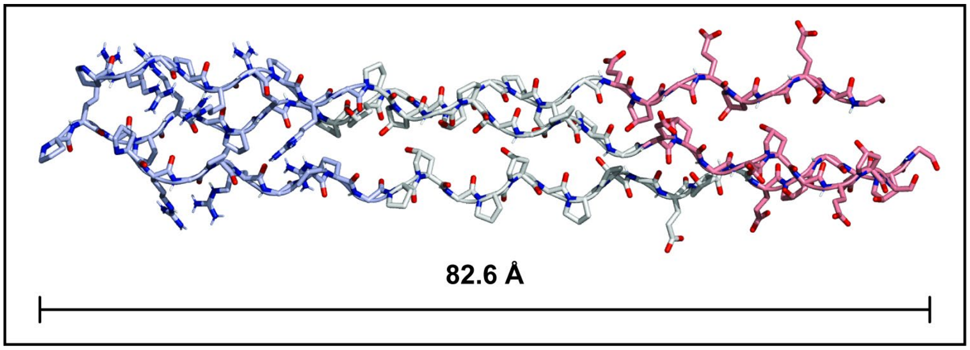

Figure S9. Contour length of **CMP333** obtained from Pymol and built in CCbuilder.[1]

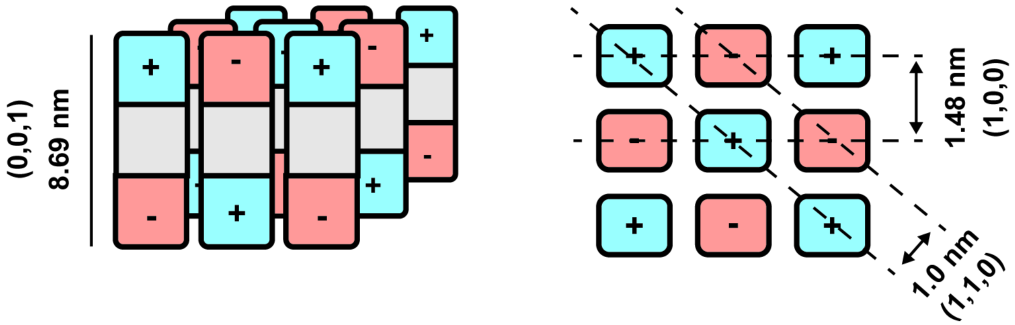

Figure S10. Inter-planar spacings for **CMP333** 2D tetragonal lattice.

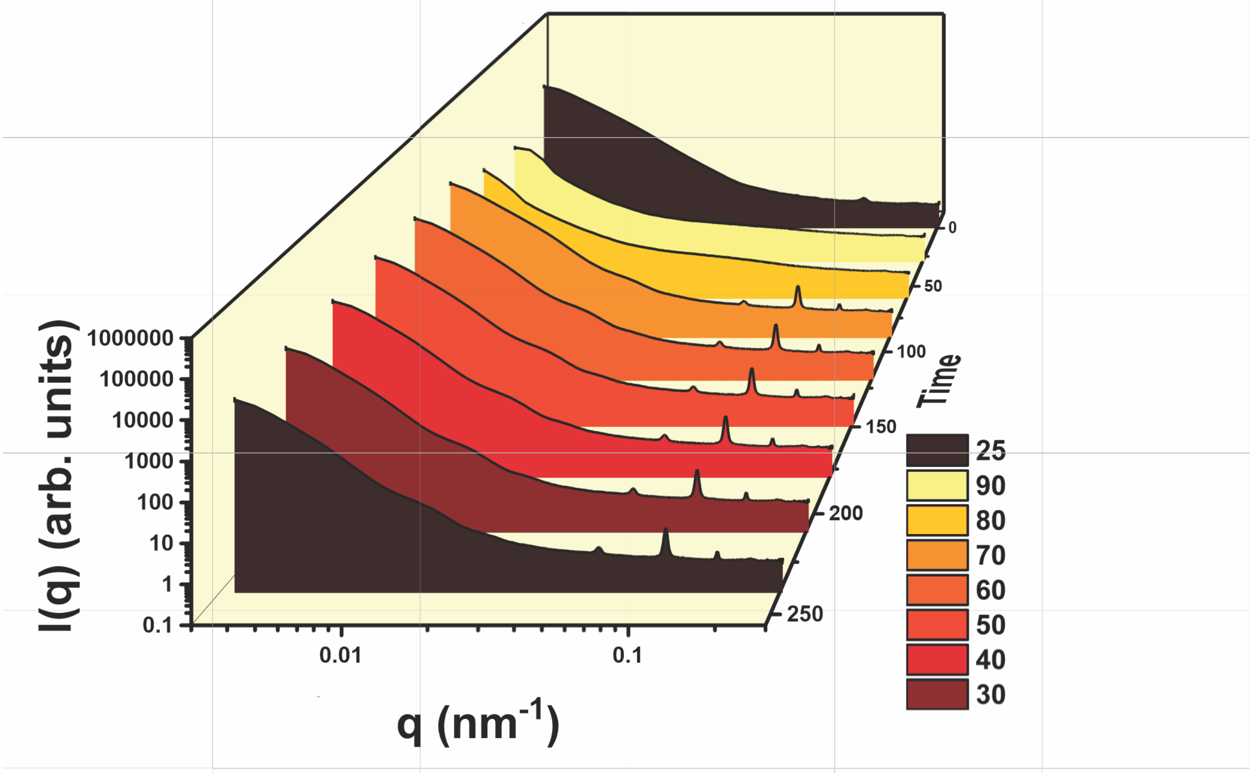

Figure S11. SAXS scattering profiles of **C_{12}333** (4 mg/mL) as a function of temperature.

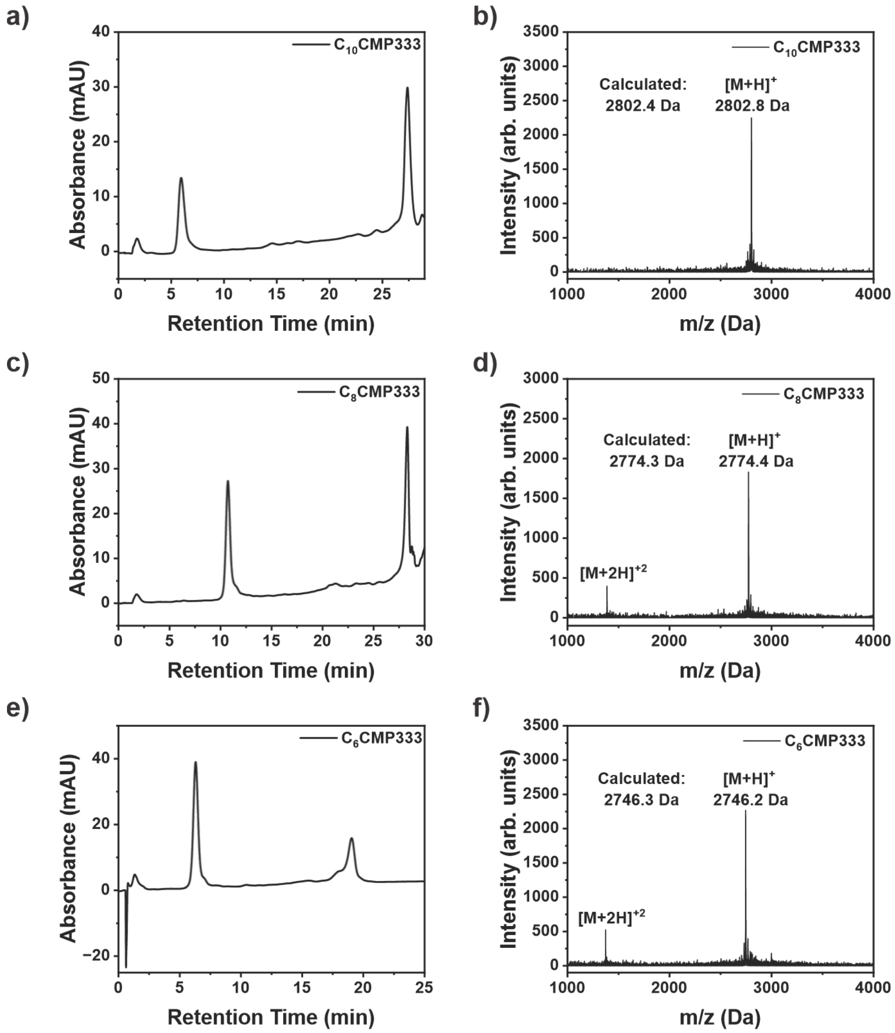

Figure S12. Analytical HPLC and MALDI-TOF MS for (**a**,**b**) **C_{10}333**, (**c**,**d**) **$C_8$333**, (**e**,**f**) **$C_6$333**. Note: both peaks display masses for the target product.

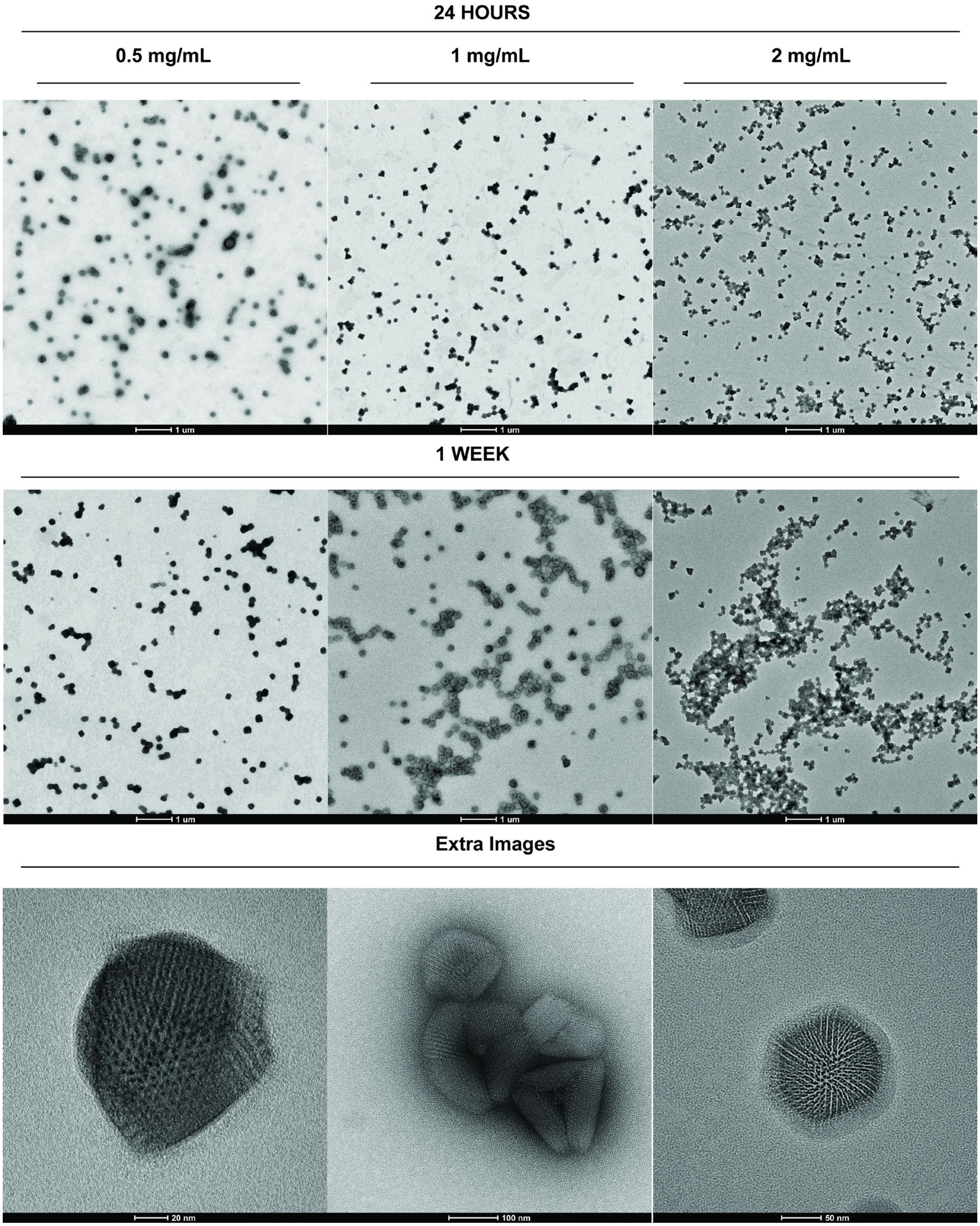

Figure S13. Stained TEM micrographs of **C_{10}333** at various aCMP concentrations and assembly time points.

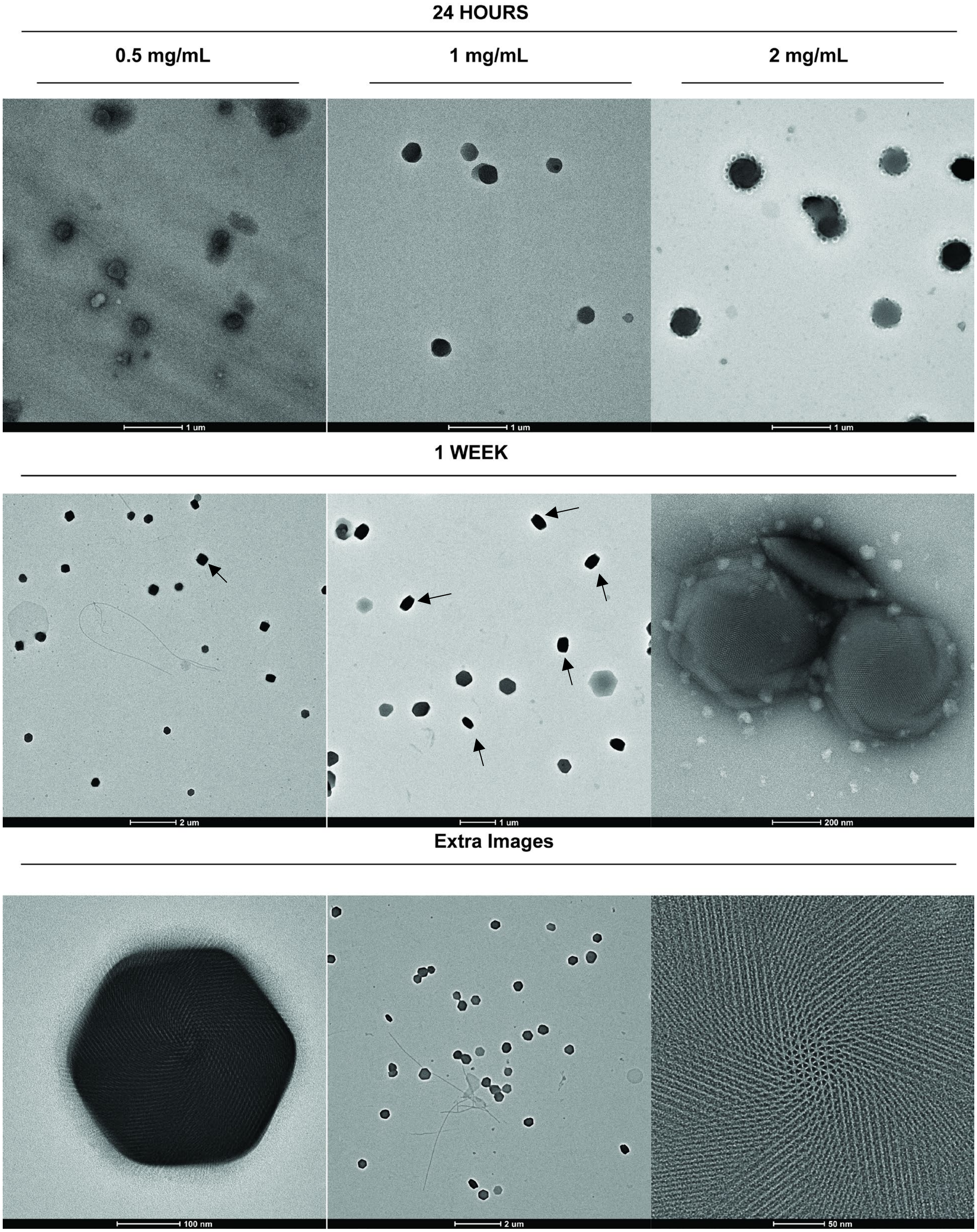

Figure S14. Stained TEM micrographs of **C$_8$333** at various aCMP concentrations and assembly time points. Arrows point to crystals laying on their side highlighting their thin, disc-like feature.

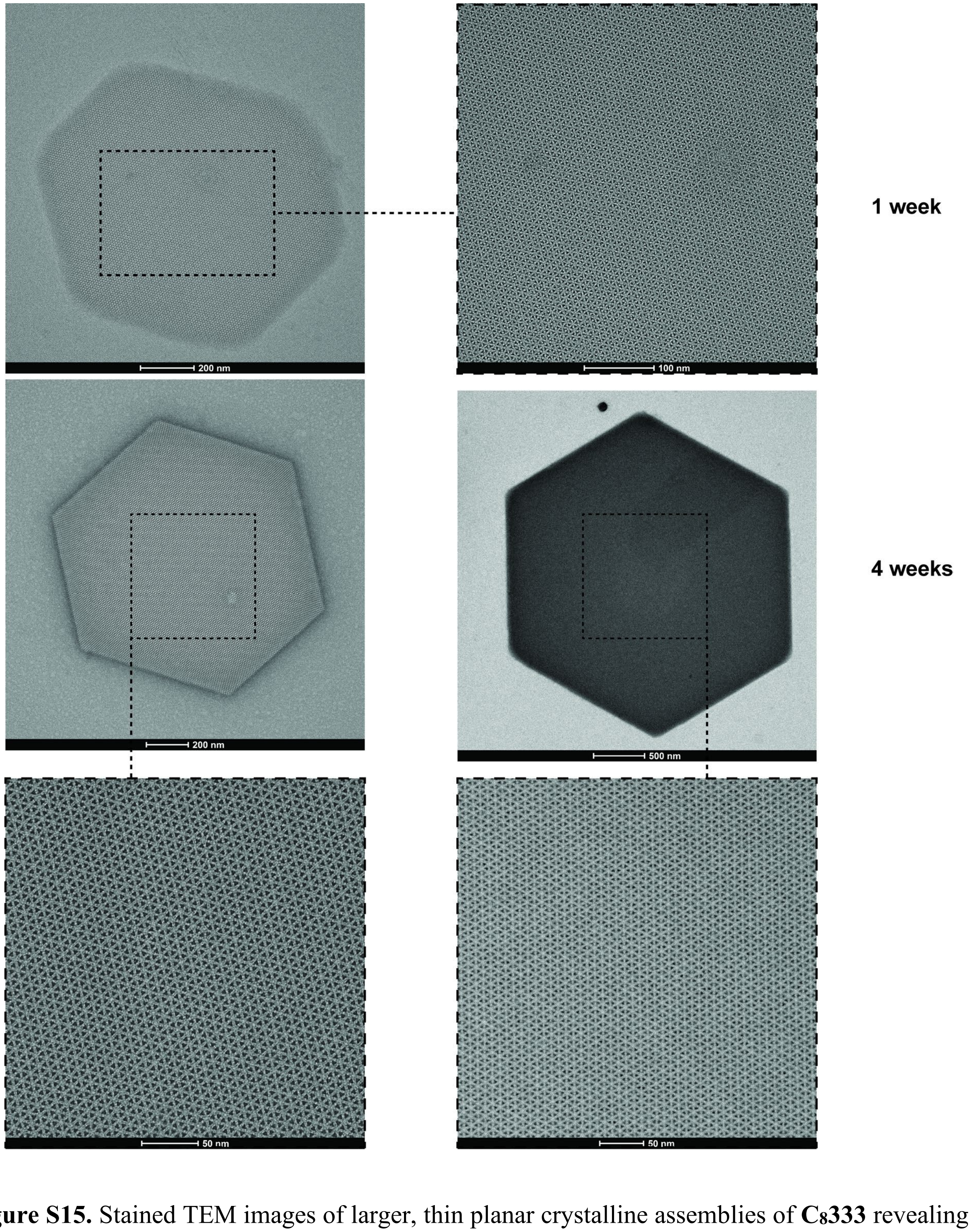

Figure S15. Stained TEM images of larger, thin planar crystalline assemblies of **$C_8$333** revealing the highly ordered internal architecture.

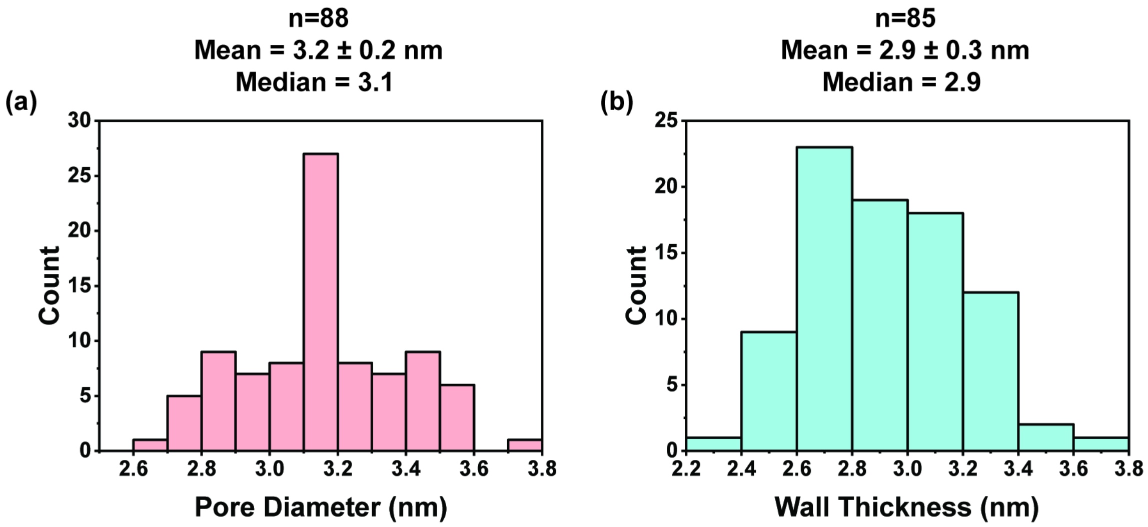

Figure S16. (**a**) Pore diameters and (**b**) wall thicknesses obtained from the large, thin, planar crystal shown in **Figure 3c**. Measurements were collected using ImageJ.

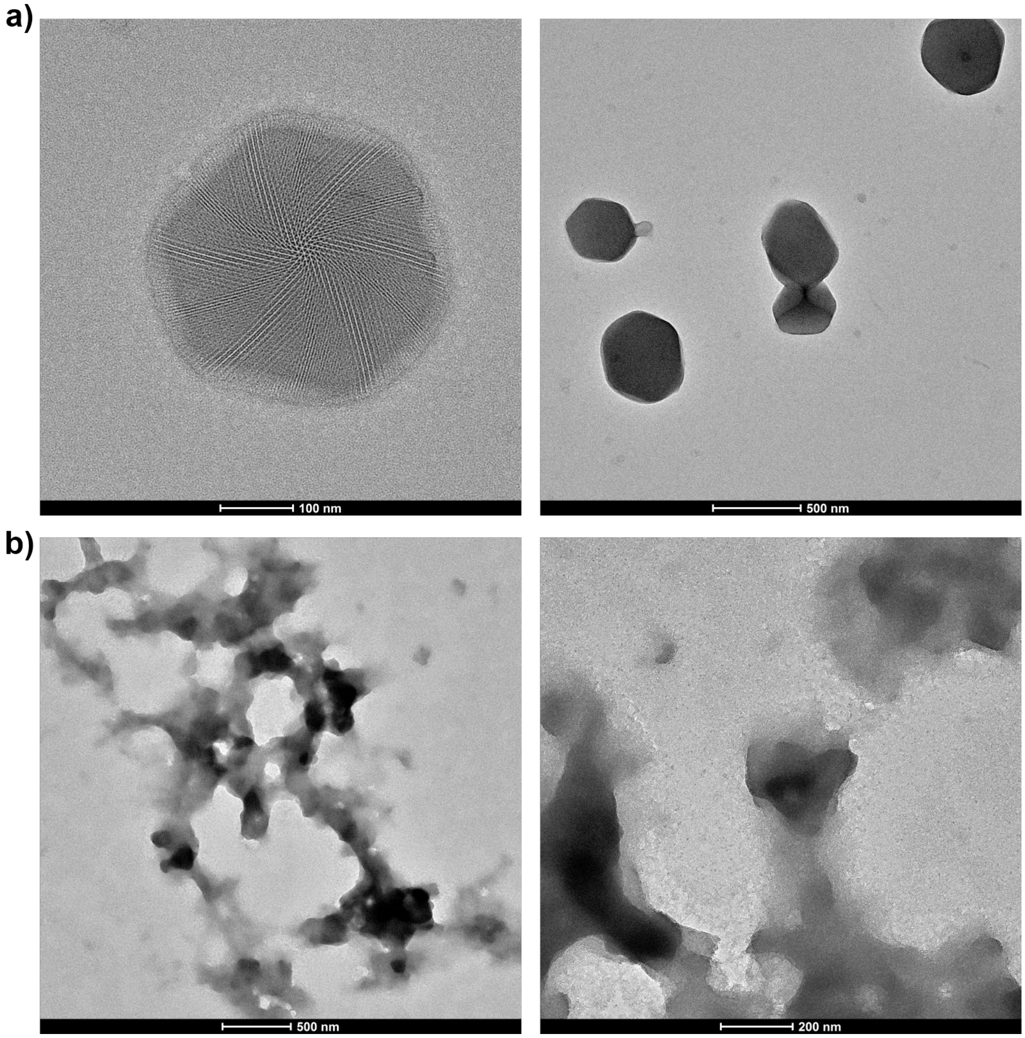

Figure S17. Stained TEM images of $\mathbf{C_8333}$ frameworks (2 mg/mL) after 10 days post-exchange in (**a**) HPLC-grade water and (**b**) PBS buffer (pH 7.4).

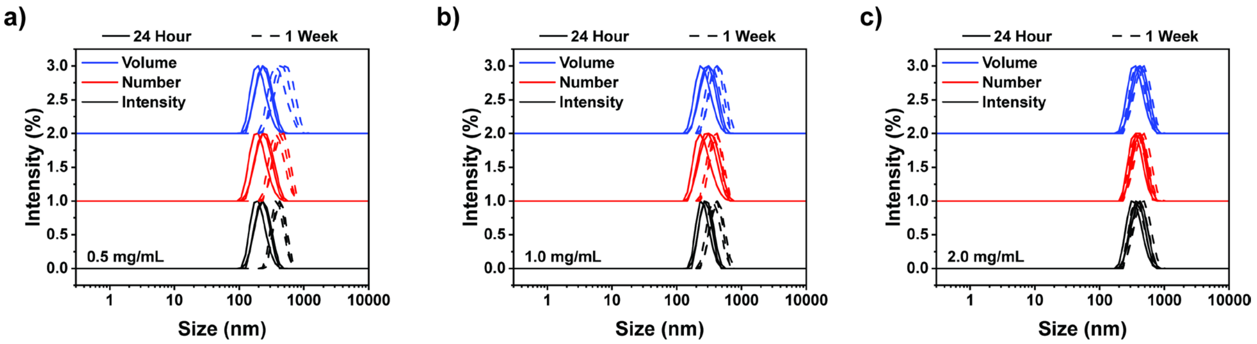

Figure S18. DLS profiles for **C10333** at (**a**) 0.5 mg/mL, (**b**) 1 mg/mL, and (**c**) 2 mg/mL. See **Table S1** for mean data values for each experiment.

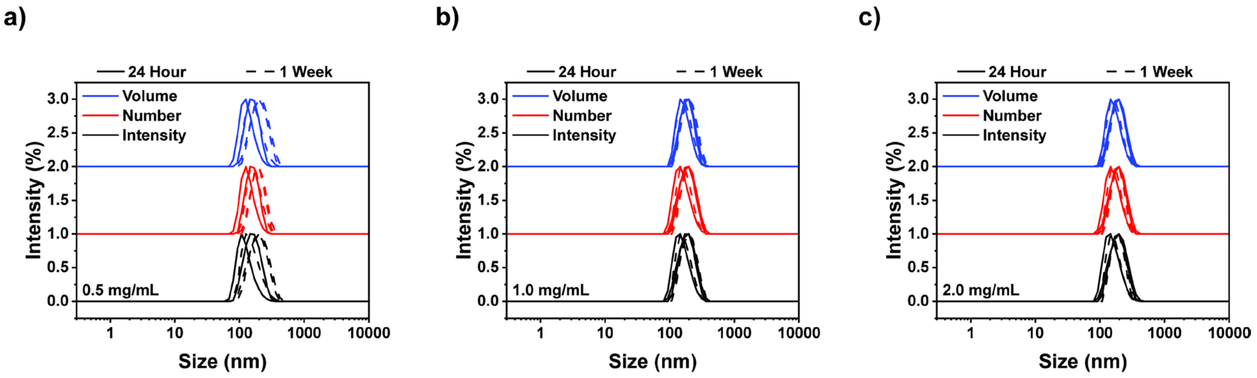

Figure S19. DLS profiles for **C8333** at (**a**) 0.5 mg/mL, (**b**) 1 mg/mL, and (**c**) 2 mg/mL. (**d**) See **Table S1** for mean data values for each experiment.

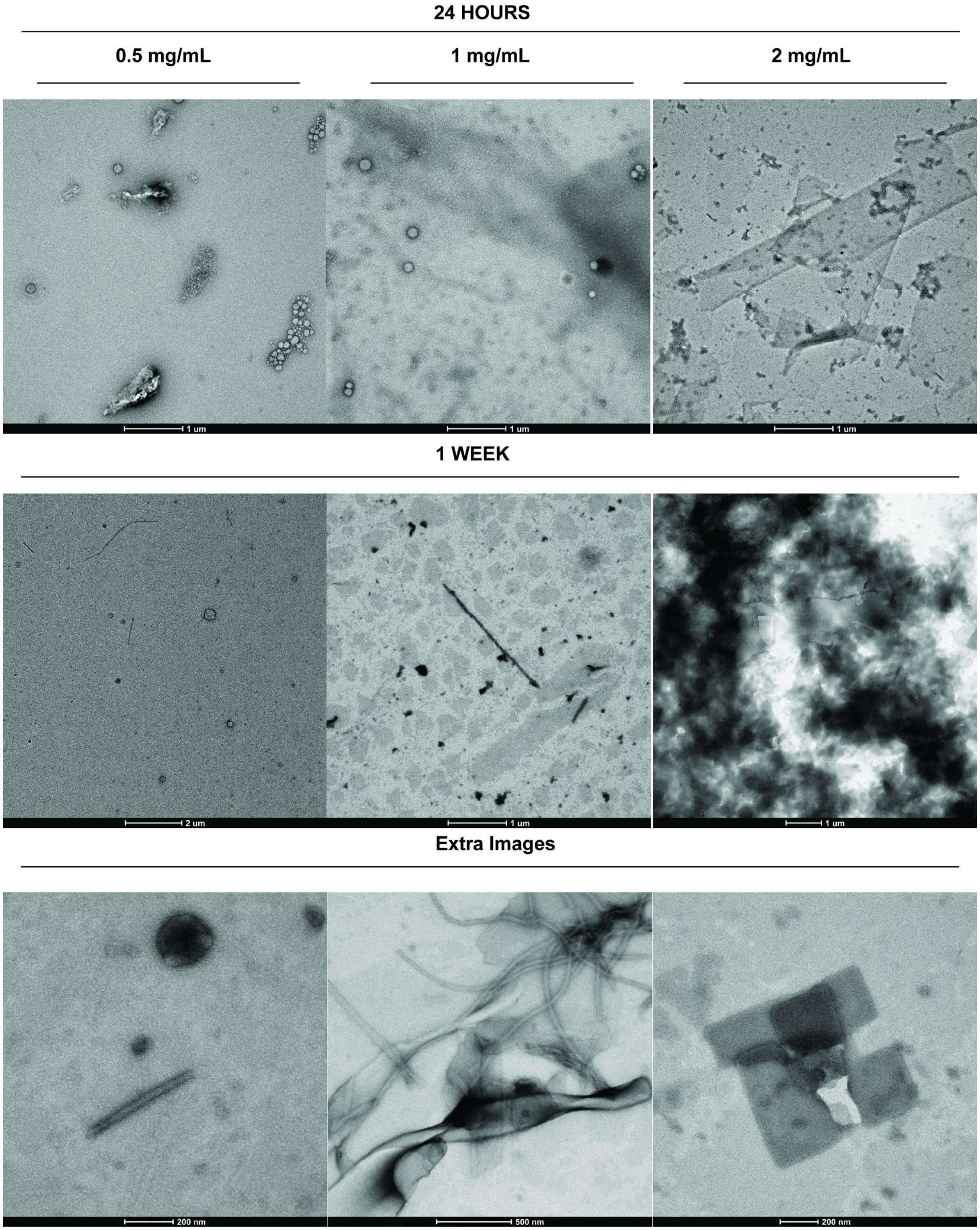

Figure S20. Stained TEM micrographs of **$C_6$333** at various aCMP concentrations and assembly time points.

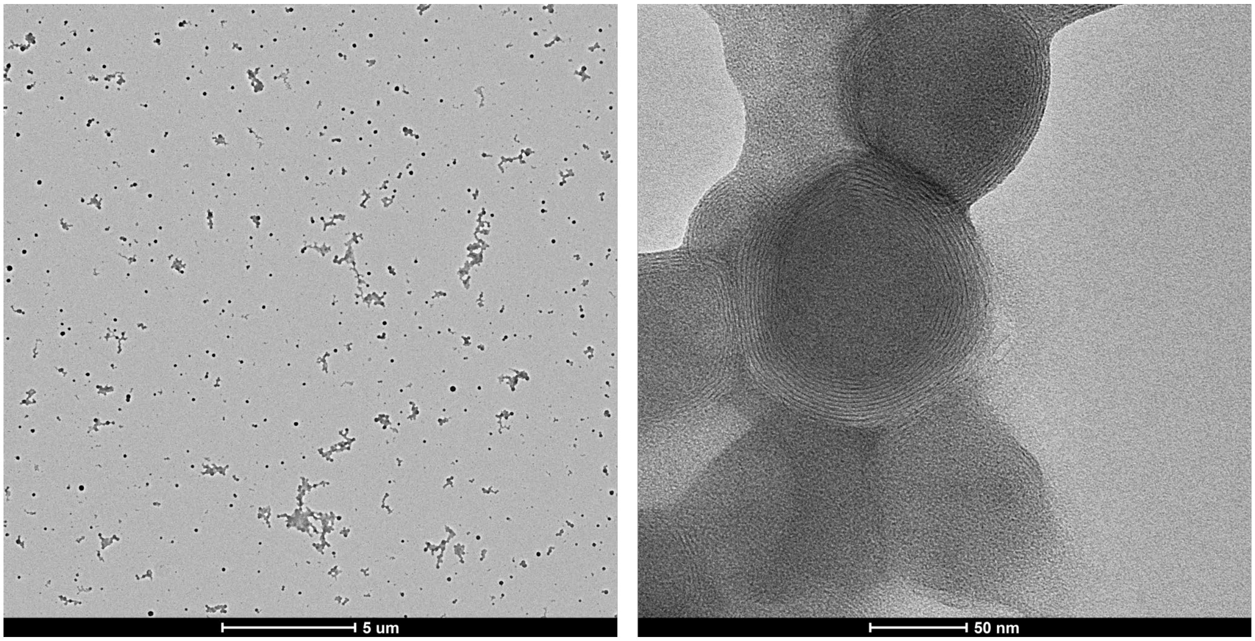

Figure S21. Stained TEM images of assembly of octanoic acid with **CMP333** (1:1 molar ratio) in 20 mM MES buffer (pH 6.0) after 2 weeks of assembly time. CMP333 concentration = ~1 mg/mL.

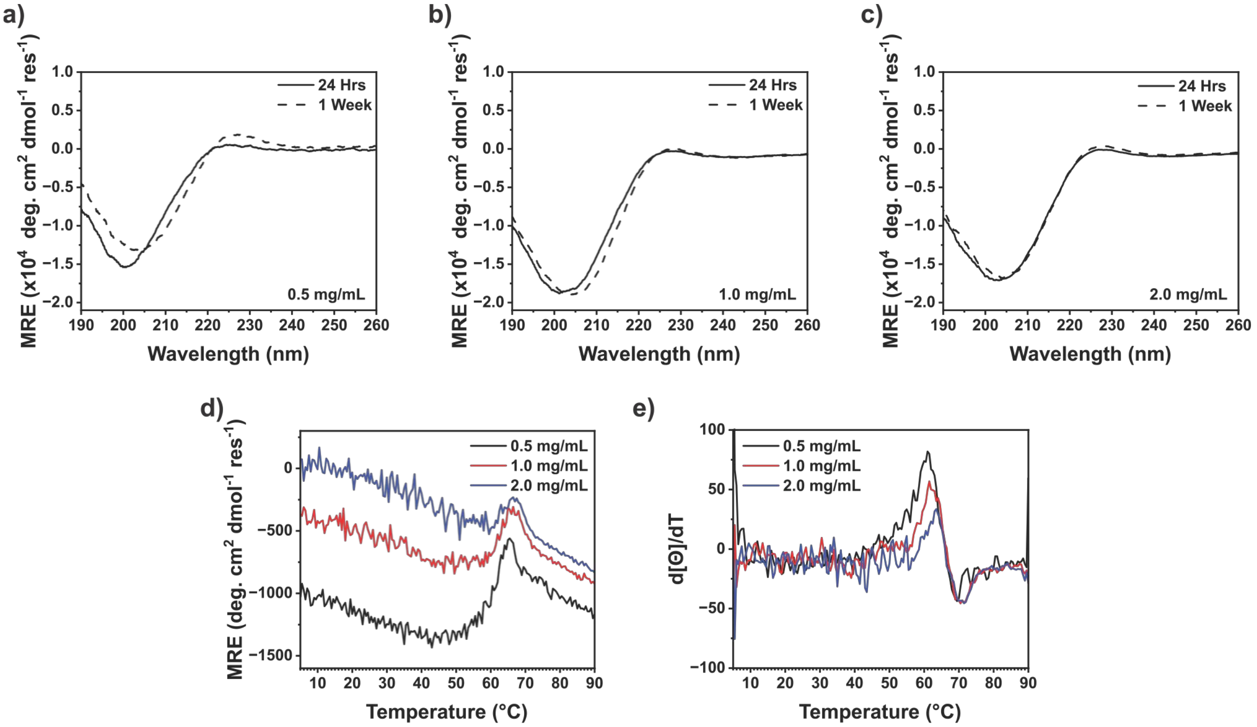

Figure S22. CD spectra of **C10333** at (**a**) 0.5 mg/mL, (**b**) 1 mg/mL, and (**c**) 2 mg/mL. (**d**) CD thermal denaturation plots of **C10333**. (**e**) First derivative of the CD signal at 224 nm as a function of temperature for **C10333** at various concentrations.

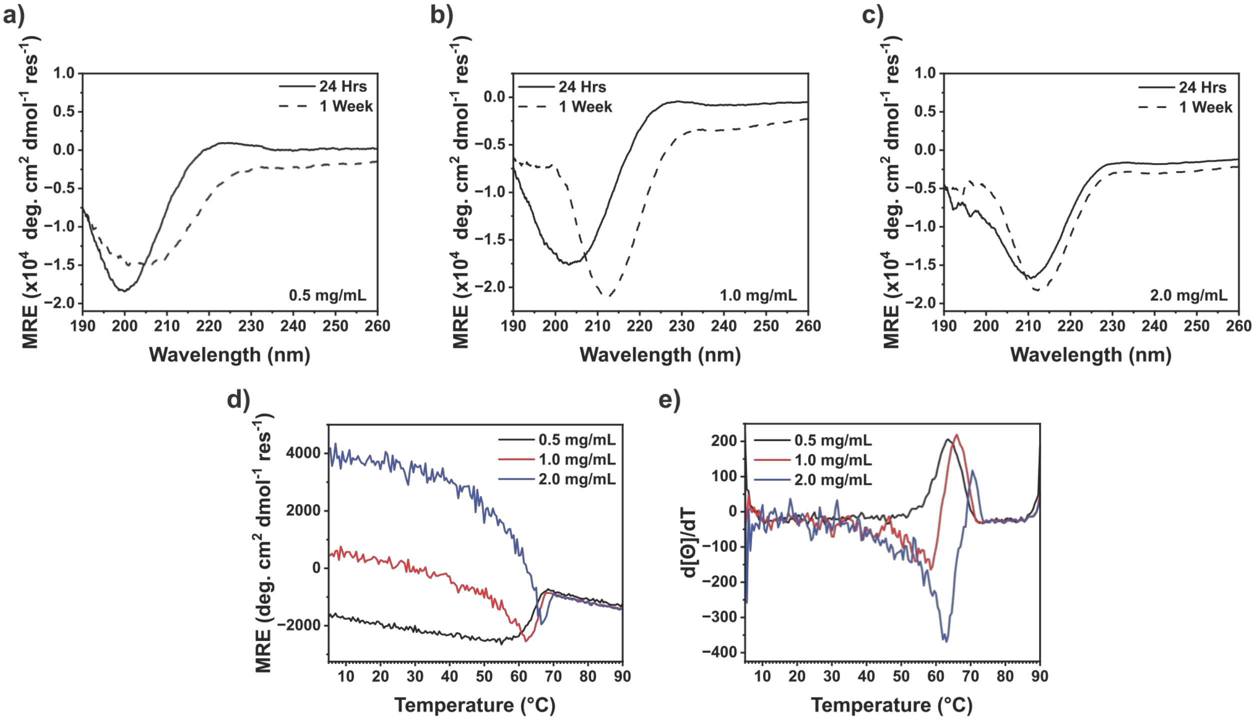

Figure S23. CD spectra of **C8333** at (**a**) 0.5 mg/mL, (**b**) 1 mg/mL, and (**c**) 2 mg/mL. (**d**) CD thermal denaturation plots of **C8333**. (**e**) First derivative of the CD signal at 224 nm as a function of temperature for **C8333** at various concentrations.

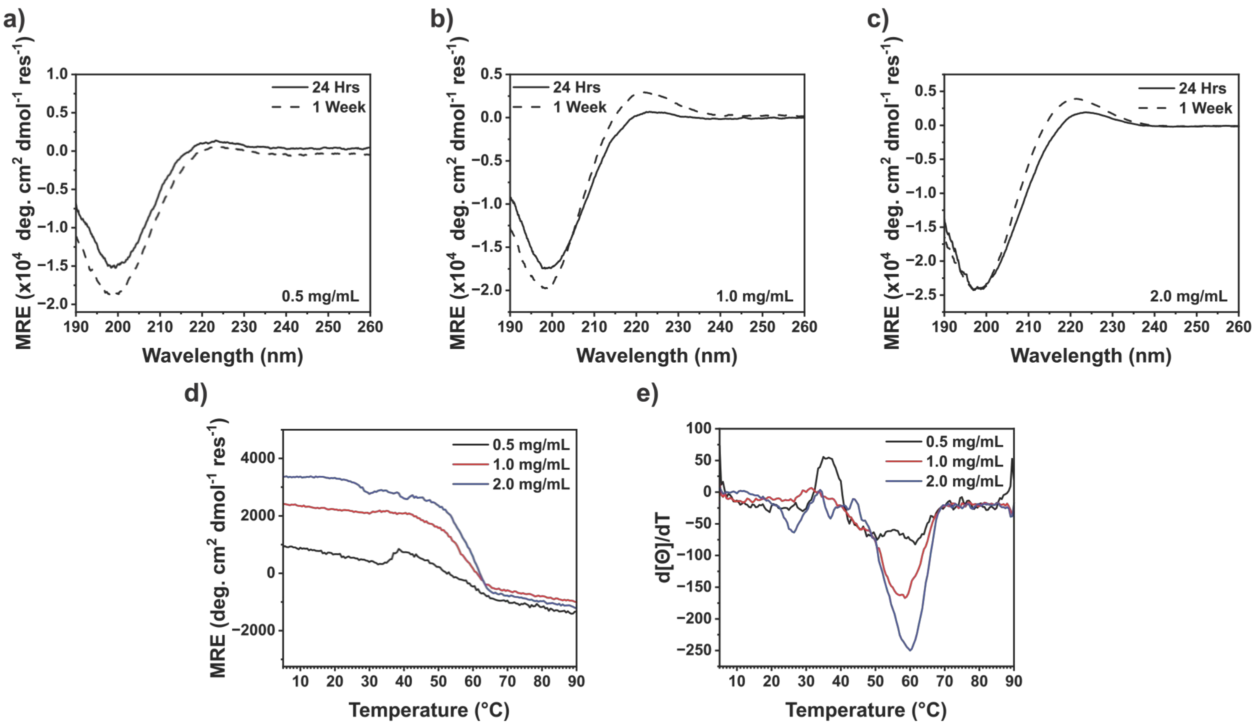

Figure S24. CD spectra of **$C_6$333** at (**a**) 0.5 mg/mL, (**b**) 1 mg/mL, and (**c**) 2 mg/mL. (**d**) CD thermal denaturation plots of **$C_6$333**. (**e**) First derivative of the CD signal at 224 nm as a function of temperature for **$C_6$333** at various concentrations.

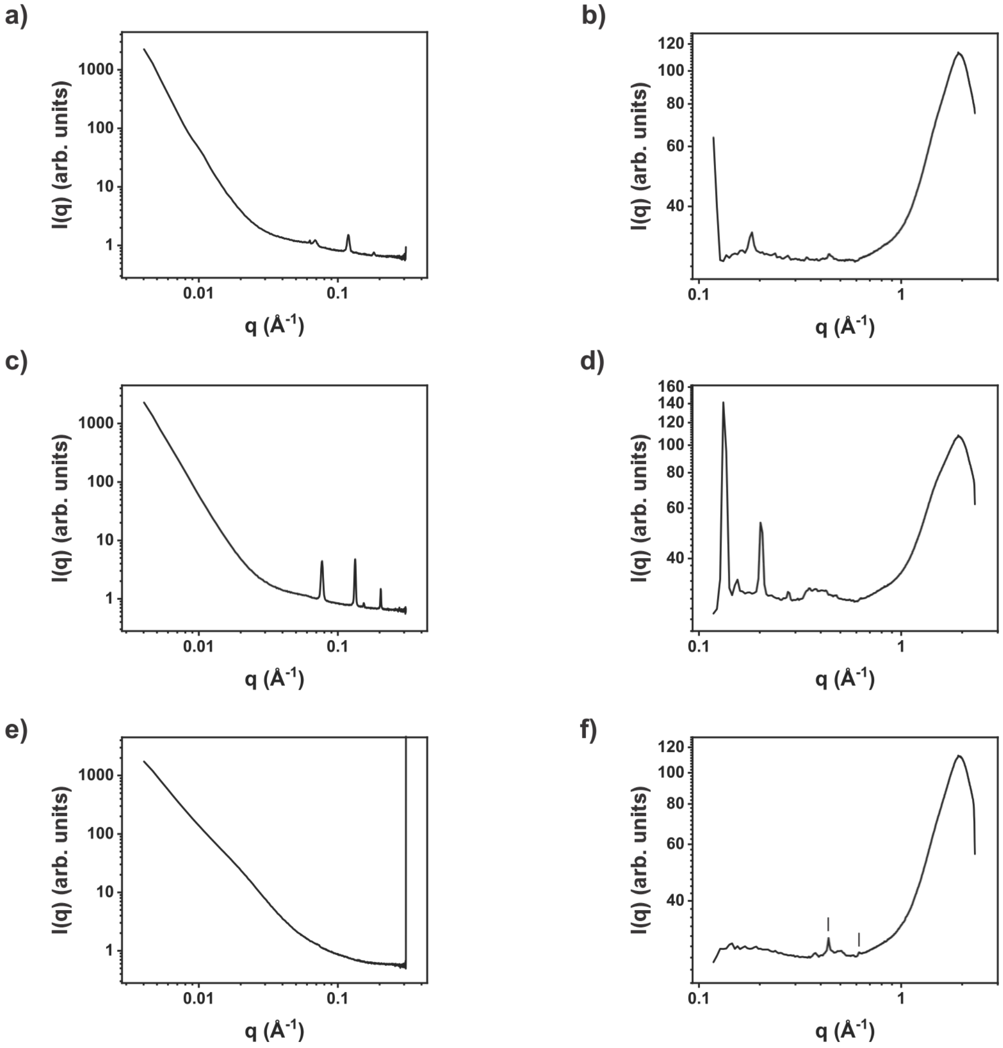

Figure S25. SAXS/WAXS scattering profiles for (**a**,**b**) **C_{10}333**, (**c**,**d**) **$C_8$333**, (**e**,**f**) **$C_6$333**. Tabulated q-values and *d*-spacings are shown in **Table S3**.

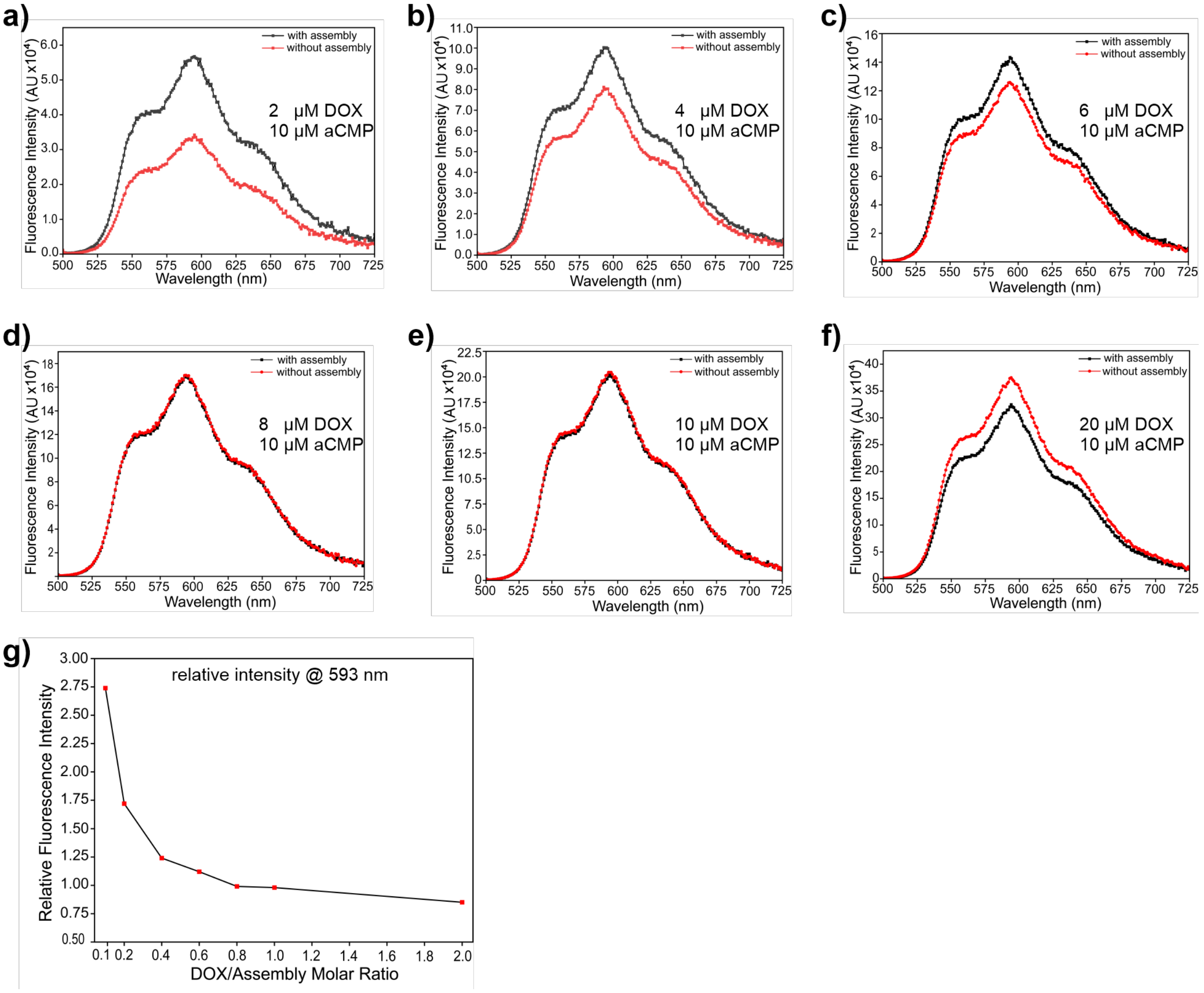

Figure S26. Fluorescence emission spectra of DOX with, and without, the presence of aCMP frameworks at the following molar ratios (DOX:aCMP): (**a**) 0.2, (**b**) 0.4, (**c**) 0.6, (**d**) 0.8, (**e**) 1.0, (**f**) 2.0. All fluorescence spectra were collected after three hours of DOX incubation in 10 µM aCMP frameworks. (g) Relative emission intensity at 593 nm of DOX in the presence of aCMP assemblies and DOX alone.

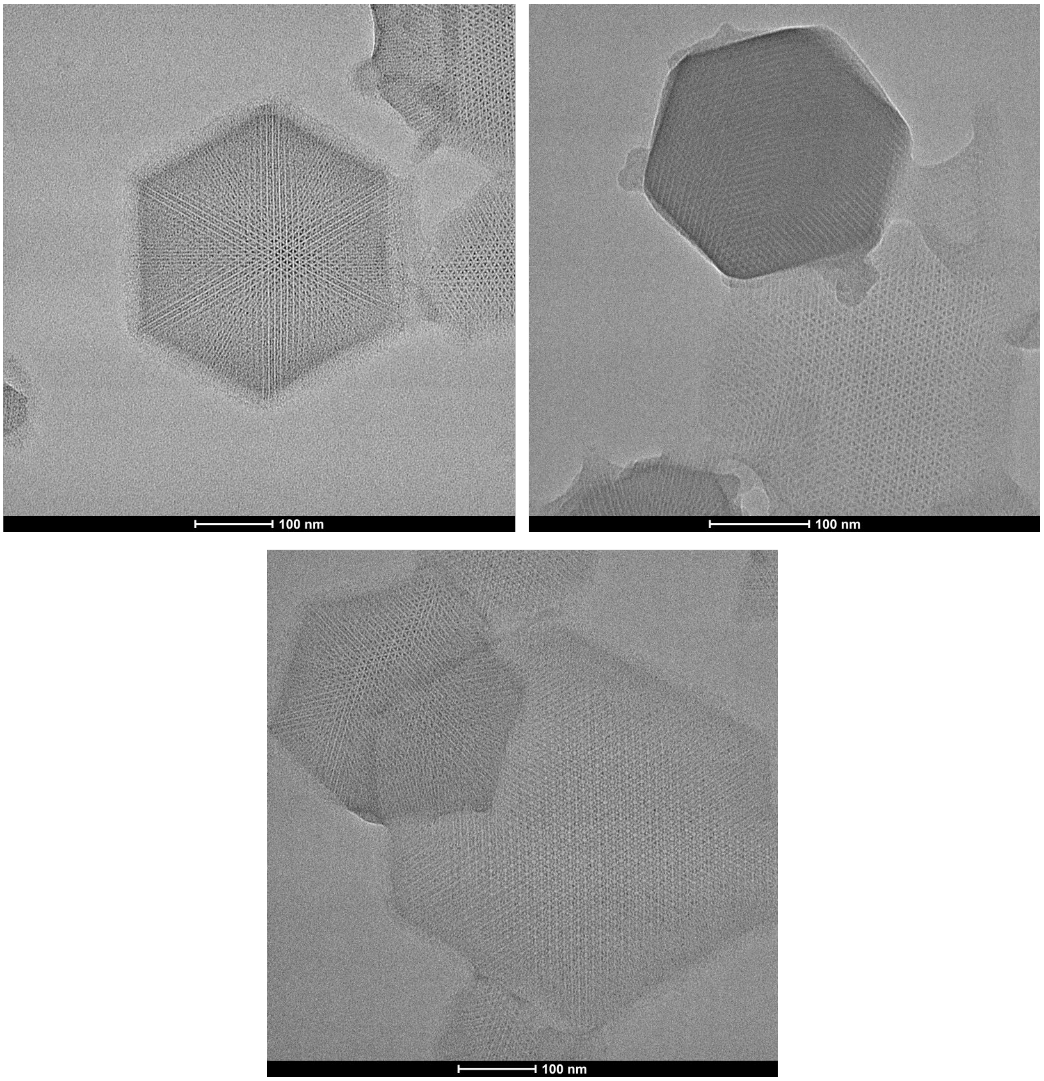

Figure S27. Stained TEM image of DOX (500 μM) + **C₈333** frameworks (10 μM) after 1 day of incubation in 20 mM MES buffer (pH 6.0).

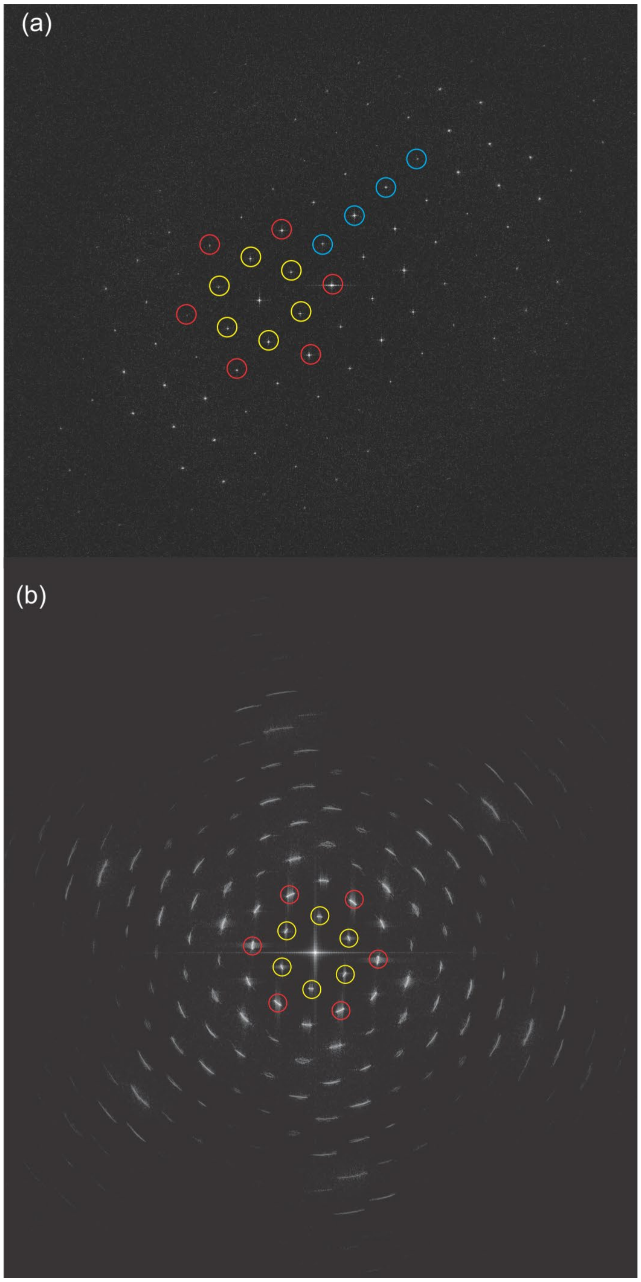

D (Å)	D (Å)	D (Å)
1. 84.82	7. 49.19	13. 42.47
2. 84.96	8. 48.95	14. 28.51
3. 84.21	9. 48.60	15. 21.34
4. 84.18	10. 49.19	16. 17.01
5. 84.27	11. 48.73	
6. 84.47	12. 48.60	

D (Å)	D (Å)
1. 81.39	7. 46.95
2. 81.07	8. 46.73
3. 81.30	9. 46.82
4. 81.30	10. 46.65
5. 81.26	11. 46.86
6. 81.45	12. 46.86

Figure S28. FFTs and interplane distances obtained from cryo-EM micrographs of **C8333** for (**a**) the thin, planar crystal and (**b**) multi-faceted crystal that are shown in **Figure 6**.

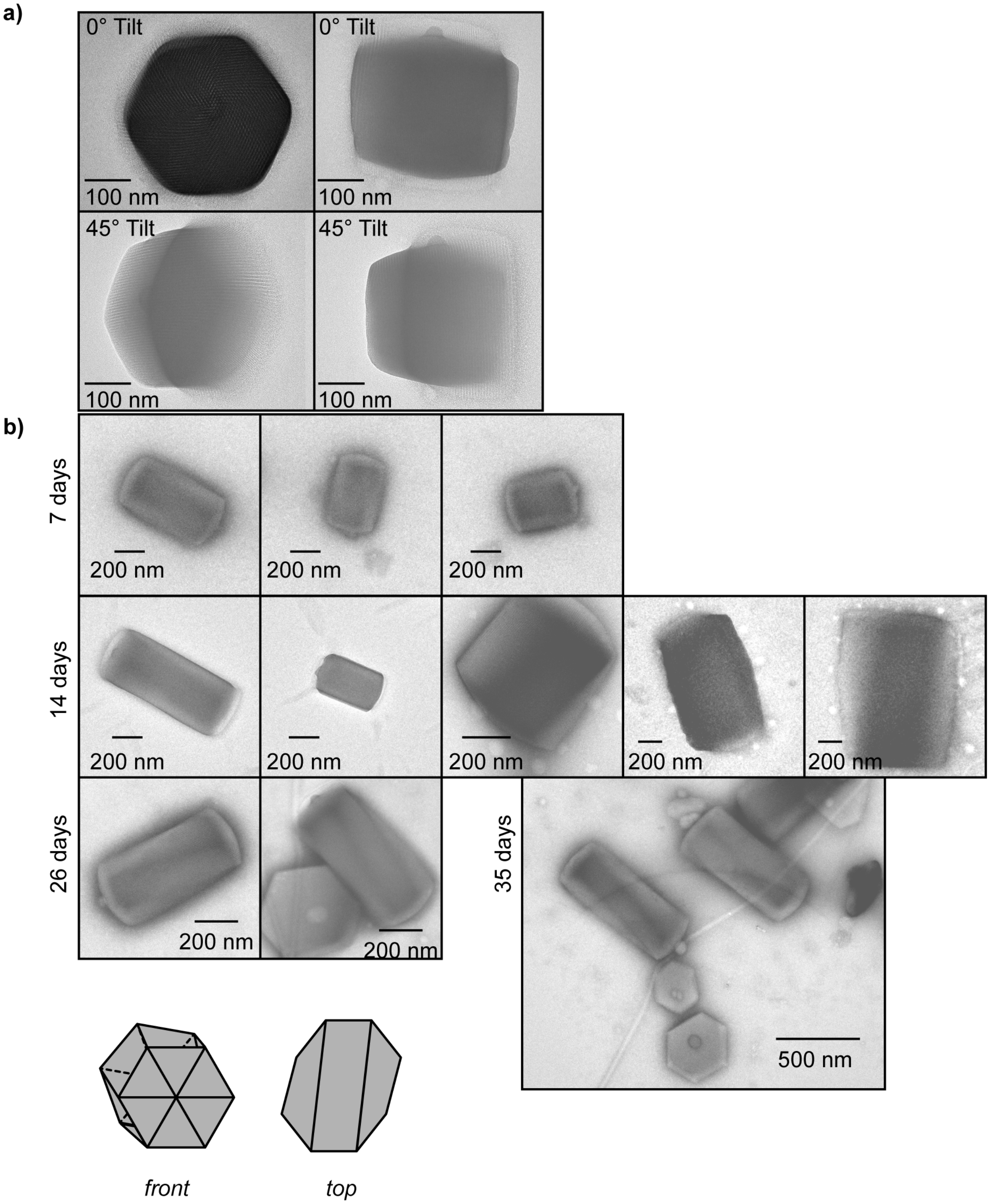

Figure S29. (**a**) Tilted images of **C8333** and (**b**) stained TEM images of **C8333** crystals assembled under extra slow cooling. The twisting of the hexagonal bipyramidal assemblies can be observed.

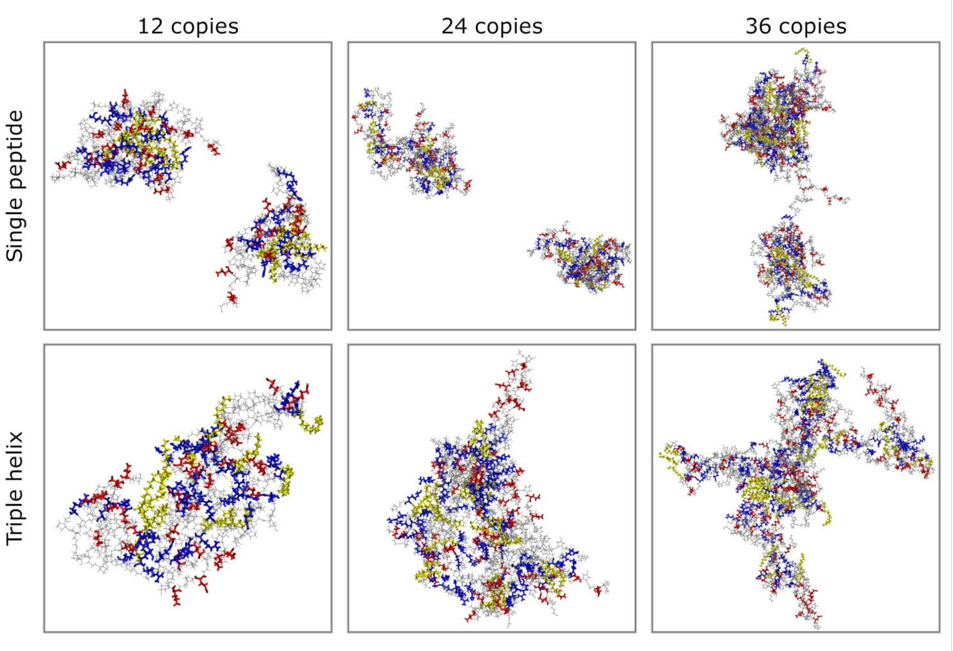

Figure S30. Final simulation trajectories after 500 ns for single peptide starting and for triple helix starting position with either 12, 24, or 26 copies of **C_{12}333**.

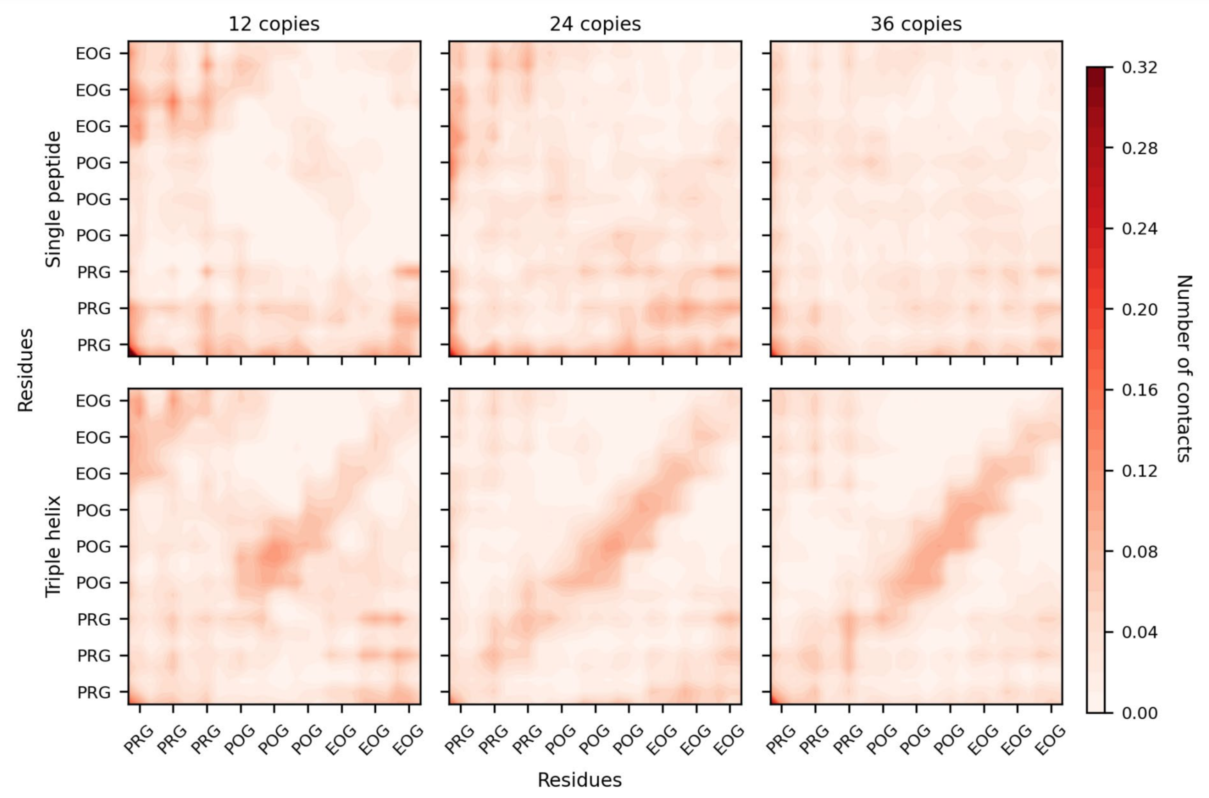

Figure S31. Contact maps showing intermolecular interactions between residues throughout the duration of the simulation (within 5 Å)

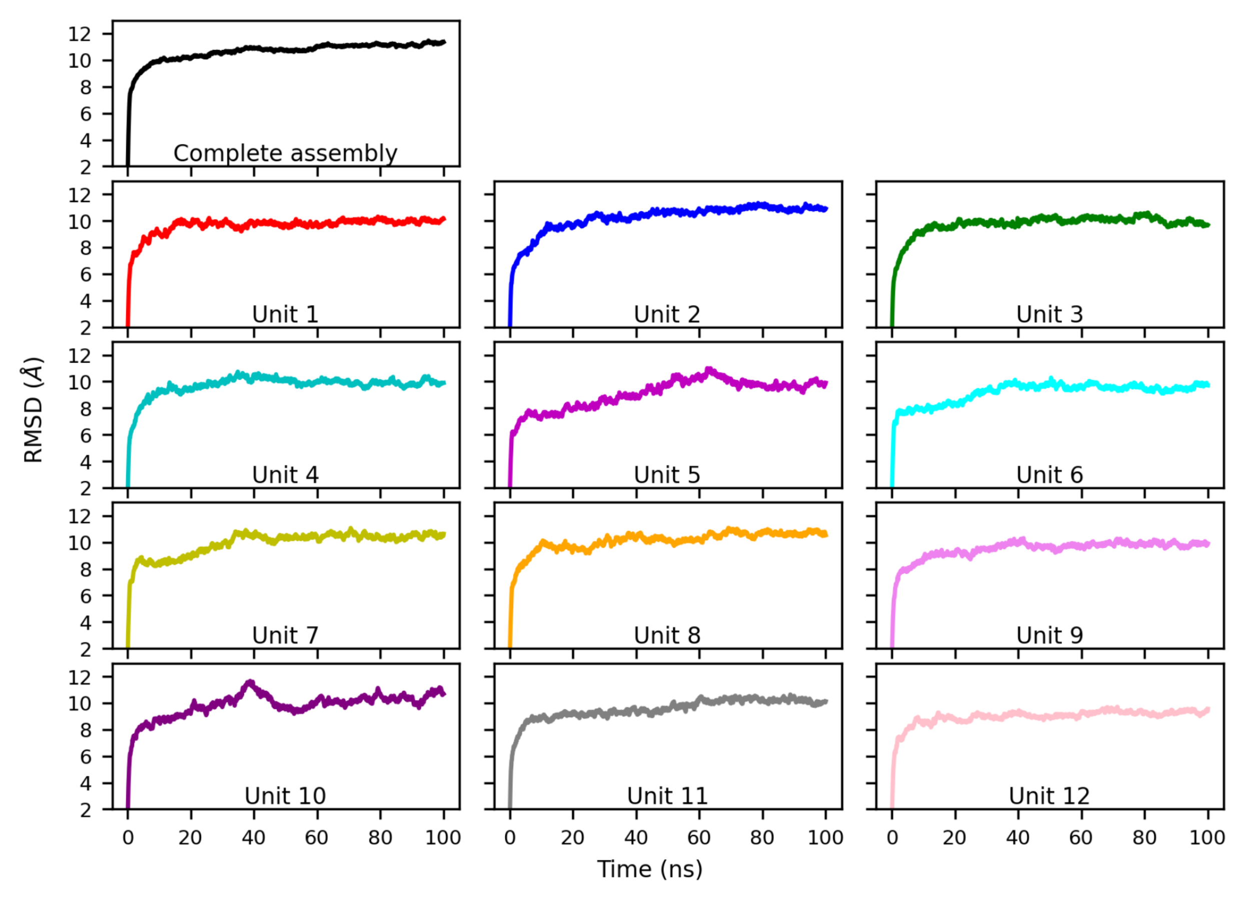

Figure S32. RMSD values of C_α as a function of simulation time for each **C₈333** hexagonal units after superimposing the C_α positions in the trajectory to the initial structure

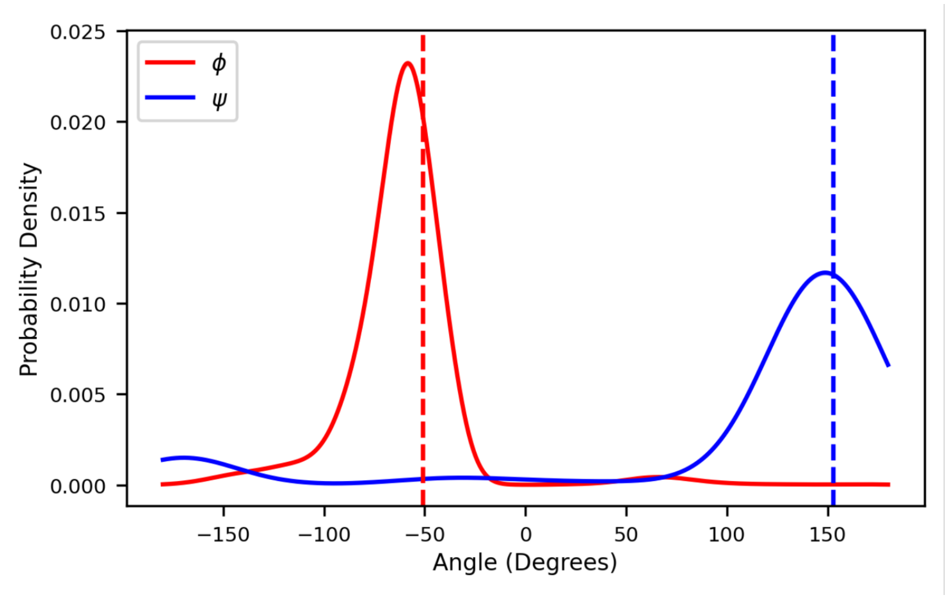

Figure S33. Probability density of simulated **C₈333** assembly packing model. The dash lines show expected φ and ψ values for collagen triple helices.[2]

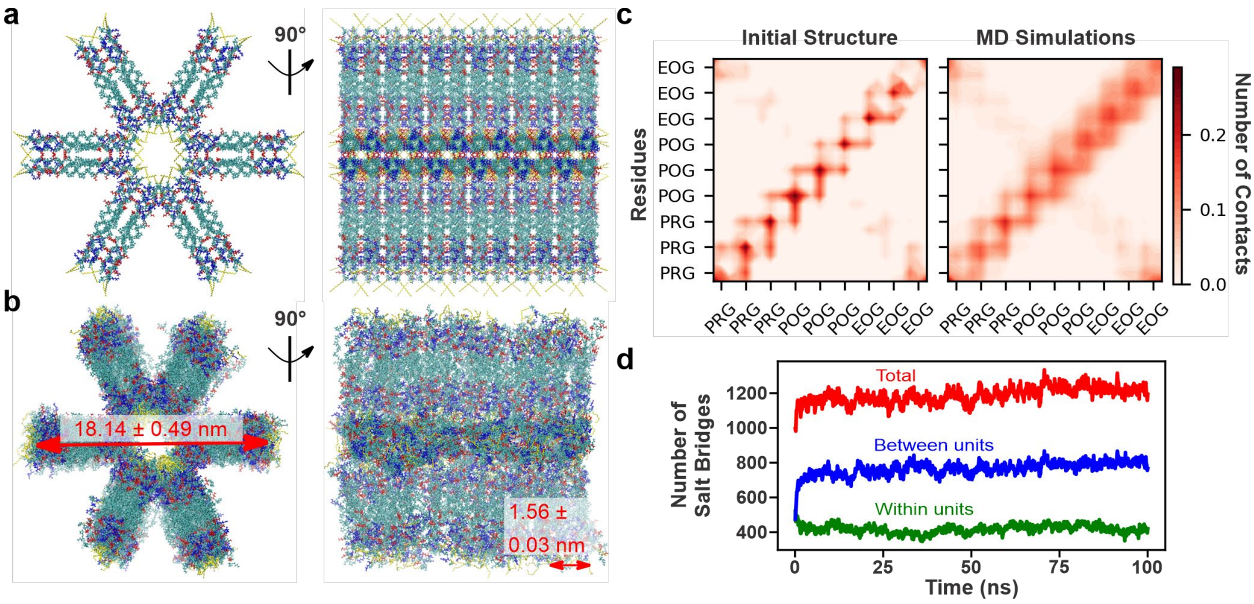

Figure S34. (a) Initial MD simulation trajectory hub-and-spoke assembly packing model for **C**$_{12}$**333**. The assembly systems comprises 12 hub-and-spoke models (hexagonal units) that are constructed from 12 **C**$_{12}$**333** triple helices. (b) Final MD simulation trajectory of the proposed assembly packing model after 100 ns of simulation. (c) Contact maps for individual residues within aCMPs. Contacts along the diagonal axis highlight intra-and inter-triple helical interactions within collagen triple helices and between antiparallel-packed triple helices, respectively. (d) Number of salt bridge contacts within a single hexagonal unit (416.1 ± 23.7), between hexagonal units (778.3 ± 29.9), and for all units within the assembly (1199.0 ± 42.9).

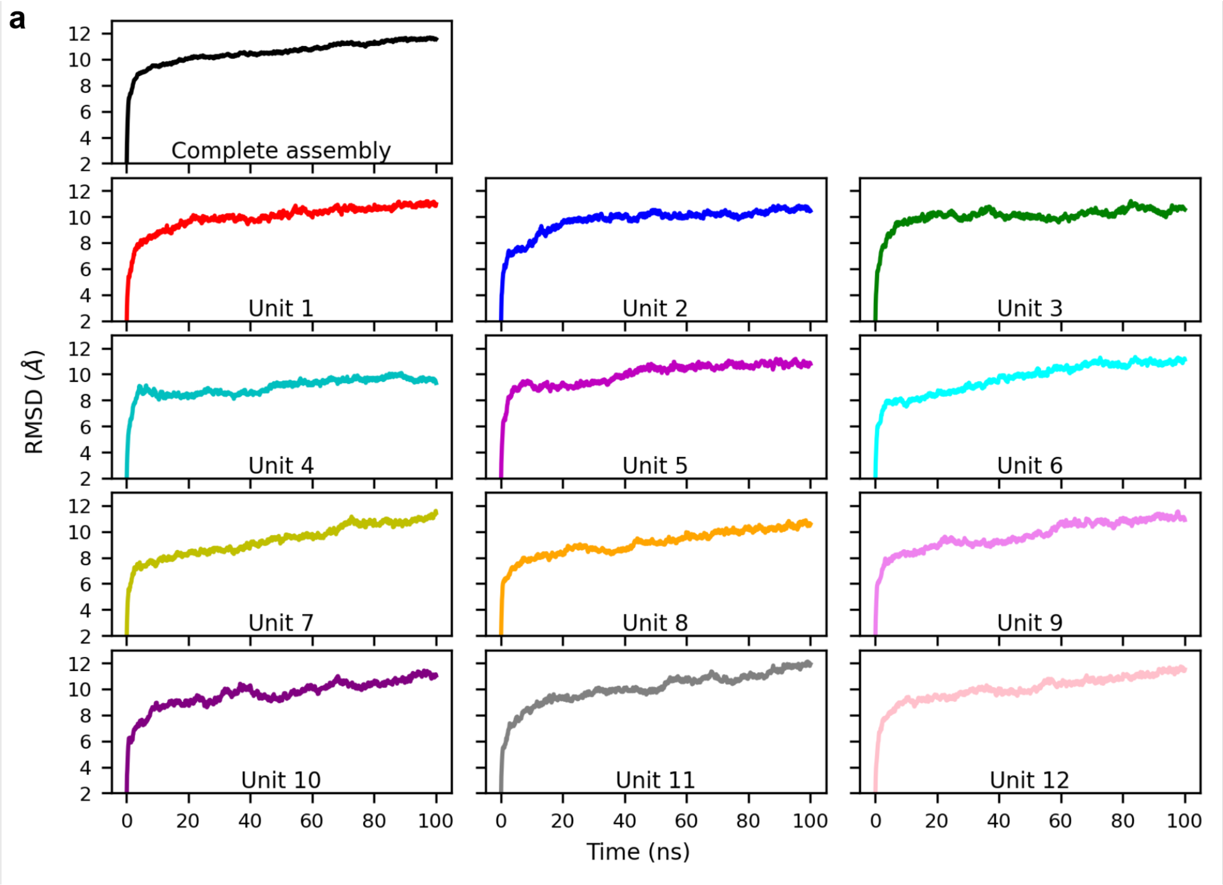

Figure S35. RMSD values of C_α as a function of simulation time for each **C_{12}333** hexagonal units after superimposing the C_α positions in the trajectory to the initial structure.

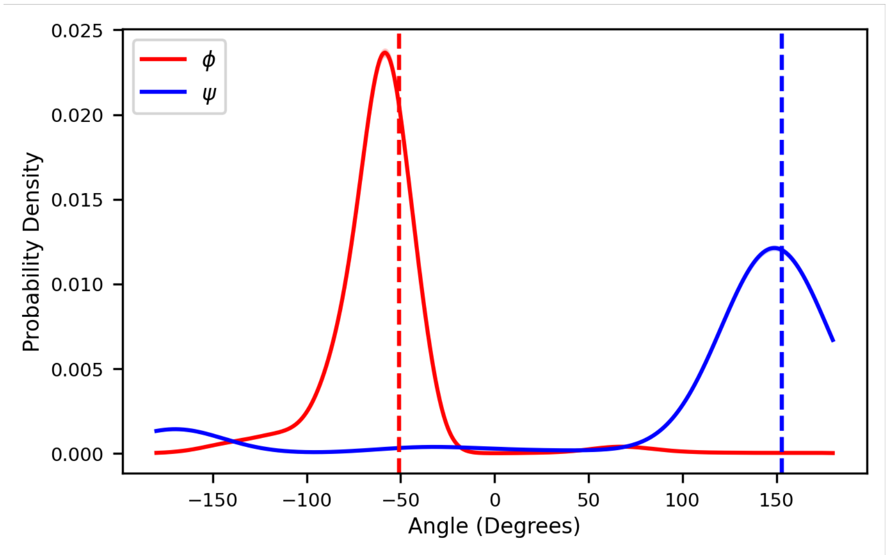

Figure S36. Probability density of simulated **C$_{12}$333** assembly packing model. The dash lines show expected φ and ψ values for collagen triple helices.[2]

Table S1. Tabulated DLS data for peptides described in the main text.

Peptide	Conc (mg/mL)	TimePoint	Intensity	Std Dev	Number	Std Dev	Volume	Std Dev
CMP333	0.5	24 Hours	478.5	31.2	457.4	24.94	500.8	39.43
CMP333	0.5	7 Days	1393	156.9	1316	115.2	1368	122.5
CMP333	1.0	24 Hours	674.3	181.4	670	177.6	686.4	184.6
CMP333	1.0	7 Days	1362	144.7	1308	119.7	1349	121.4
CMP333	2.0	24 Hours	948.9	49.91	926.4	43.52	959	48.58
CMP333	2.0	7 Days	1170	112.1	1134	101.5	1178	110.5
C12CMP333	0.5	24 Hours	147.2	2.975	122	1.95	141.7	2.476
C12CMP333	0.5	7 Days	147.6	1.244	123.7	0.1105	142.6	1.09
C12CMP333	1.0	24 Hours	177.1	1.997	150.6	0.4759	177	2.143
C12CMP333	1.0	7 Days	176.6	3.003	145.9	1.026	175.8	3.223
C12CMP333	2.0	24 Hours	201.6	3.124	152.5	10.51	204.3	2.897
C12CMP333	2.0	7 Days	202.3	3.304	156.8	11.52	205.5	3.184
C10CMP333	0.5	24 Hours	162.2	1.96	131.2	5.833	158.3	0.6852
C10CMP333	0.5	7 Days	202.6	6.014	166.2	8.93	206.3	6.808
C10CMP333	1.0	24 Hours	190.8	0.8464	160.9	5.281	192.8	0.8264
C10CMP333	1.0	7 Days	200.1	0.7669	173	1.791	203.6	0.8508
C10CMP333	2.0	24 Hours	194.8	1.426	163.4	3.736	197.3	1.391
C10CMP333	2.0	7 Days	193.6	2.537	171.2	2.629	196.3	2.846
C08CMP333	0.5	24 Hours	238.6	5.987	205	2.324	247.3	7.442
C08CMP333	0.5	7 Days	440	25.29	401.1	9.166	473.6	44.4
C08CMP333	1.0	24 Hours	299.4	14.6	261.7	2.847	315.3	21.2
C08CMP333	1.0	7 Days	410.5	5.723	380.3	1.542	432.3	10.14
C08CMP333	2.0	24 Hours	402	8.585	365.5	8.911	427	10.99
C08CMP333	2.0	7 Days	439.9	3.571	399.4	4.037	473.2	3.355

Table S2. Table of transition melting temperatures as determined by the first derivative plot of the CD thermal denaturation curves.

	Tm (°C)		
	0.5 mg/mL	1 mg/mL	2 mg/mL
C12	66	69	72
C10	69	70	71
C8	NA	59	63
C6	55	58	60
C2	54	56	57
C0	62	63	63

Table S3. Tabulated *q*-values and *d*-spacings for all CMP and aCMP assemblies reported in the manuscript.

C_{12}333		C_{10}333		$C_8$333		$C_6$333		CMP333	
q (1/Å)	*d* (nm)	q (1/Å)	*d* (nm)	q (1/Å)	*d* (nm)	q (1/Å)	*d* (nm)	q (1/Å)	*d* (nm)
0.0667	9.42	0.0685	9.17	0.0772	8.14	-	-	0.0723	8.69
0.117	5.37	0.119	5.29	0.134	4.70	0.146	4.31	0.145	4.34
0.131	4.81	0.182	3.45	0.154	4.08	0.377	1.67	0.424	1.48
0.178	3.52	0.238	2.64	0.204	3.08	0.437	1.44	0.604	1.04
0.234	2.69	0.275	2.28	0.275	2.28	0.498	1.26		
0.266	2.36	0.340	1.85	0.349	1.80	0.618	1.02		
0.308	2.04	0.437	1.44						
0.336	1.87								
0.373	1.69								
0.400	1.57								
0.447	1.41								
0.461	1.36								

REFERENCES

1 Wood, C. W.; Woolfson, D. N. CCBuilder 2.0: Powerful and Accessible Coiled-Coil Modeling. *Protein Science* **2018**, *27* (1), 103–111. https://doi.org/10.1002/pro.3279.

2 Nelson, D. L. & Cox, M. M. The Three-Dimensional Structure of Proteins. In Lehninger Principles of Biochemistry (8th edn) Ch. 4, 106–146 (W. H. Freeman & Company, New York, 2021).
