# Supplementary material for: Mesoporous peptide frameworks engineered from crystallizable collagen-mimetic peptide amphiphiles
Source: Nat Commun. 2026 May 15;17:6919. doi: 10.1038/s41467-026-73068-2 (PMC13388958; doi:10.1038/s41467-026-73068-2)
Supplement: Supplementary file 2 — Transparent Peer Review file [file 41467_2026_73068_MOESM2_ESM.pdf]

nature portfolio

Peer Review File

Mesoporous peptide frameworks engineered from crystallizable collagen-mimetic peptide amphiphiles

Corresponding Author: Professor Andrea Merg

This file contains all reviewer reports in order by version, followed by all author rebuttals in order by version.

Parts of this Peer Review File have been redacted as indicated to maintain the confidentiality of unpublished data.

Version 0:

Reviewer comments:

Reviewer #1

(Remarks to the Author)
The authors designed new crystalline porous materials based on collagen and report mesoporous frameworks from aCMPs with different hydrophobic chain lengths. They have used TEM, Cryo-TEM, SAXS/WAXS, FFT analysis, CD and DLS spectra to characterize the peptide materials. They have also used fluorescent probe to characterize the lipid environment. These charge-segregated aCMPs with alkyl chains create a frustrated system in which the hydrophobic and electrostatic interactions compete with each other to yield diverse packing during self-assembly. The authors didn't report any applications in this paper but they mentioned these peptide materials can have potential applications in sensing, separations and encapsulation.

The novel points of this paper are:
1. First 3-D porous crystal using collagen triple helix
2. They can engineer the size of the pores rationally
3. Decent resolution structural characterization, which is important for the field of peptide assembly. Unfortunately, resolution is not molecular- or atom-level. There is substantial support for the formation of the crystalline material and how the assembly does change with the addition and extension of the alkyl chains. The structural characterization still seems slightly unsupported due to the various instances of inferring from the characterization methods, but there doesn't seem to be a better means of further elucidating the structure.

The main criticisms are:
1. The introduction states that prior examples required restrictions on AAs, but collagen is even more restricted in that in requires the P-HP-G motif that can only be changed to P-X-G in small amounts. This work also seems to require the electrostatic patterning, further restricting the Xs to be Arg and Glu. There should be some examples where a "free" position is changed to another side-chain to demonstrate that these frameworks can have diverse functionality. Then only it will be general design principle and used for multiple applications. Thus far, they mainly showed the effect of the alkyl chain on the assembly, rather than the effect of the peptide.
2. Though collagen frameworks are novel, it is not clear why making collagen frameworks would be a large advance given that there are many examples of alpha-helical frameworks that are more engineerable than collagen.
3. Given that these are 3D crystals (stated in Abstract), why isn't there more crystallographic information?
a. What are the space groups?
b. Can electron diffraction be obtained at other angles to more reliably index the spots?
c. Can the authors comment on why these cannot grow larger into single crystals?
d. While the abstract calls these 3D crystals, most figures and the main text refers to them as 2D crystals. This should be better clarified.
4. The paper would be greatly strengthened if they showed that the mesopores could uptake some large molecule guest.

Overall, without demonstrating the advantages of a collagen framework over existing peptide frameworks or any application, it is difficult to recommend publication in Nature Communications as is. The main body of the paper focuses on characterization of the structures but falls short of demonstrating the promise of an engineerable class of functional materials. However, the reviewer recognizes that, given the dearth of well-characterized peptide assemblies, that this paper does make an important contribution to the field in that regard.

Specific comments:
1. https://doi.org/10.1021/ja0758990 similar sequence except lacking alkyl tail, published in JACS 2007, design and characterization similar, also this paper has done some MD simulations
2. State the value of the Tm. (Page 4)
3. The “electrostatic domain dominates” and “hydrophobic and electrostatic domains engaged” panels, the alkyl chain should be attached with N-terminus (the + end). (Fig. 1c)
4. PAGE 6/Paragraph 1 – “in line with previous reports” - needs citation
5. Written as C12333, but in SI and many figures (for example Fig. S1d), the term is written as C12CMP33
6. In Figure 2h, q3 for C12333 is within the noise levels
7. What does “(See above)” on page 16 mean? Does it refer to Figure 3?
8. C6 is states as close to “tipping point” (Page 10) but given that C8 forms two different morphology, would it be accurate to call it the actual tipping point?
9. No yields mentioned of peptides.
10. “While these strategies have yielded impressive porous architectures, including mesoporous frameworks” – ref 21 (Ganatra et al) also has two examples of mesoporous peptide frameworks.
11. Discuss why only even length alkyl chains were chosen. In other words, why not use C7 or C9?
12. Authors should make a computational model of the unit cell and superimpose onto the TEM image to show that proposed structure is consistent with the data.
13. In the introduction after “…or have used oligoprolines and/or noncanonical amino acids to rigidify the assembly building block”, there should be added references for 3 recent papers on this topic:
a. J. Am. Chem. Soc. 2025, 147, 20, 17404–17415
b. J. Am. Chem. Soc. 2025, 147, 20, 17433–17447
c. https://pubs.acs.org/doi/full/10.1021/jacs.5c03534

Reviewer #2

(Remarks to the Author)
This manuscript described a crystalline mesoporous peptide framework (MPF) assembled from amphiphilic collagen-mimetic peptides (aCMPs). The authors systematically demonstrated how hydrophobic lipid tails and electrostatic interactions drive the formation of hexagonally ordered frameworks with tunable pore sizes. Structural characterization is thorough, employing TEM, cryo-TEM, SAXS/WAXS, and CD spectroscopy. The study presents a hub-and-spoke assembly model and contributes to the design of biologically derived porous materials with potential biomedical applications. However, several important issues should be addressed to improve the mechanistic rigor and functional depth of the study.
1. Validation of the Hub-and-Spoke Assembly Model
The model is primarily inferred from low-resolution cryo-TEM and SAXS data, without computational or atomic-level structural validation. The authors should consider molecular dynamics or coarse-grained simulations to support their proposed packing geometry and clarify whether collagen triple helices are essential for bridging hubs.
2. Lack of Direct Evidence for CMP Bridging
The hypothesis that two aCMP helices bridge each hub pair is speculative without direct visualization. Unless confirmed by cryo-ET or molecular modeling, the structural model should be presented as a working hypothesis rather than a definitive mechanism.
3. Missing Functional Demonstration of Guest Encapsulation
Although MPFs are proposed as mesoporous materials capable of encapsulating hydrophilic and hydrophobic molecules, no experimental data are provided. A simple proof-of-concept guest loading/release experiment (e.g., dyes or proteins) would significantly strengthen the study.
4. No Stability Evaluation Under Physiological Conditions
All characterizations were conducted in MES buffer at pH 6.0. The structural integrity of MPFs at pH 7.4, in serum, or under enzymatic conditions should be tested to support claims of biomedical relevance.
5. Potential Contribution from Free Fatty Acids
For long-chain aCMPs (e.g., C12), the role of lipid tail self-aggregation cannot be ruled out. A control experiment with free fatty acid plus non-lipidated CMP would help decouple lipid-driven assembly from peptide-specific interactions.
6. Discrepancies Between TEM-FFT and SAXS-Derived Lattice Constants

Minor differences between d-spacings obtained via TEM and SAXS are observed. The authors should tabulate these values and briefly discuss possible sources of error, such as projection effects or differences in hydration state.

Reviewer #3

(Remarks to the Author)
The work presented is an exciting direction in the importance of peptides/proteins in the design and creation of new nanostructures in self-assembled materials that directly highlights the importance of the peptide fold/secondary structure and quaternary structure in the display of chemistry. The combination of amphiphilicity, electrostatic interactions, and specificity in display of chemical functionality is what gives rise to the beautiful lattice structures observed in the new library of aCMPs. The background is a nice discussion of MOFs, COFs, and the opportunities that peptides/proteins provide in construction of (nano) porous materials. Other aspects of the manuscript that I particularly like is the direct comparison between CMPs and aCMPs (to directly observe effects of aliphatic tail inclusion in constituent molecules). I also appreciate the kinetic study showing that with cooling temperature, the porous lattices do not form until the CMPs fold for a directional display of both the hydrophobic tails and the specific spatial display of the charged amino acids in the CMP sequence. The authors have a new, exciting platform with which they can probe the importance of both the CMP design and the attached alkyl (or other) functionalities in making new structure with potential function. The manuscript should be published in Nature Communications after addressing the minor comments below.

My intuition tells me that the length of the aliphatic tail would lead to the opposite trend in aliphatic domain gel state/crystalline state. In membranes, shorter amphiphiles are harder to crystallize/not possible to crystallize. It seems there is literature precedent for the crystallization of short alkyl tails when conjugated to CMPs or other co-assembling molecules. I'm hoping the authors could briefly discuss this in the context of the lattice since, clearly, the alkyl tails aren't residing in a pure hydrophobic layer such as in a membrane.

What keeps the lattices from growing in the c/z thickness direction? Or, do the sheets actually have multiple layers of the model structure in Figure 2, 3? The molecules that lead to the pyramidal-like particles seem to have thicker walls (while Figure 6 and the C8333 lattices are very thin). I agree with the proposed model for the molecules packing within the 2D structure with hydrophobic hubs and CMP spokes. The careful analysis of the TEM, cryo-TEM, and SAXS data very much nails down how the molecules lead to the same symmetry of lattice but with slightly different spacings. However, the nodes/hubs of hydrophobic alkyl chains provides many sites for hydrophobic interactions and the attachement of aCMPs to the hubs or for a second layer of lattice to form directly on top of the first and so in. In fact, as mentioned above, the more particle-like assembly of the aCMPs with longer tails (10 and 12 carbons) in Figures 2 and 3 suggest that the walls of the particle, made of the same hexagonal-like lattice structure, are multilayer/thicker. If the longer alkyl tails are less crystalline, perhaps they lead to more exposed hydrophobic alkyls chains that, in turn, provide the anchoring sites for multilayer lattices to grow.

A similar point to the above is the more particle-like morphology observed for the longer alkyl tails as opposed to the clearly 2D nature of the shorter tails. The model comparing the two different C8333 lattices in Figure 6e may provide a clue as to why these particle (if they are, in fact, particles) form in that the longer alkyl tails on the edges of growing lattice sheets could be quite flexible as the next layer of aCMPs attach, thus acting like a hinge and allowing a new sheet to grow at a defined angle relative to the parent sheet (or an existing sheet to attach at an angle to a parent sheet). Would the authors please comment on the apparent particle nature of the longer alkyl tail lengths/does any AFM exist to show the apparent 3D nature of these structures?

I don't think any additional experiments are needed for the current work although I am keen on finding out the differences in assembly when different pH conditions are used. Turning off one of the complementary charges, for example, would allow a direct observation of the hydrophobic assembly as suggested in Figure 1. With respect to Figure 1, it looks more like a first figure in a proposal as opposed to a manuscript since it suggests future work and what is enabled by the aCMP system. Figure 1 should be revamped to focus on the aCMPs herein and the importance of complementary charged and alkyl chain length. Already these first examples provide rich assembly/nanostructure formation. The shape shifting and hydrophobic interactions dominating panels can be saved for future aCMP work. Also, the "?" in the novel architecture panel certainly can be removed! A revamped Figure 1 would help the reader focus on the interacting molecular attributes that give rise to the beautiful behavior presented.

Version 1:

Reviewer comments:

Reviewer #1

(Remarks to the Author)
The authors have thoroughly addressed all my concerns. They've performed additional experiments that now make this article suitable for publication in Nature Communications.

(Remarks on code availability)

Reviewer #2

(Remarks to the Author)
The authors have addressed all the concerns, I donot have more comments.

(Remarks on code availability)

Reviewer #3

(Remarks to the Author)
I have read the authors' comments to my review as well as the excellent, thoughtful points made by the other reviewers. I am happy with the extensive revisions and deem the paper strengthened and appropriate for publication in Nature Communications.

(Remarks on code availability)

Open Access This Peer Review File is licensed under a Creative Commons Attribution 4.0 International License, which permits use, sharing, adaptation, distribution and reproduction in any medium or format, as long as you give appropriate credit to the original author(s) and the source, provide a link to the Creative Commons license, and indicate if changes were made.
In cases where reviewers are anonymous, credit should be given to 'Anonymous Referee' and the source.
The images or other third party material in this Peer Review File are included in the article's Creative Commons license, unless indicated otherwise in a credit line to the material. If material is not included in the article's Creative Commons license and your intended use is not permitted by statutory regulation or exceeds the permitted use, you will need to obtain permission directly from the copyright holder.
To view a copy of this license, visit https://creativecommons.org/licenses/by/4.0/

REVIEWER COMMENTS

Reviewer #1 (Remarks to the Author):

The authors designed new crystalline porous materials based on collagen and report mesoporous frameworks from aCMPs with different hydrophobic chain lengths. They have used TEM, Cryo-TEM, SAXS/WAXS, FFT analysis, CD and DLS spectra to characterize the peptide materials. They have also used fluorescent probe to characterize the lipid environment. These charge-segregated aCMPs with alkyl chains create a frustrated system in which the hydrophobic and electrostatic interactions compete with each other to yield diverse packing during self-assembly. The authors didn't report any applications in this paper but they mentioned these peptide materials can have potential applications in sensing, separations and encapsulation.

The novel points of this paper are:
1. First 3-D porous crystal using collagen triple helix
2. They can engineer the size of the pores rationally
3. Decent resolution structural characterization, which is important for the field of peptide assembly. Unfortunately, resolution is not molecular- or atom-level. There is substantial support for the formation of the crystalline material and how the assembly does change with the addition and extension of the alkyl chains. The structural characterization still seems slightly unsupported due to the various instances of inferring from the characterization methods, but there doesn't seem to be a better means of further elucidating the structure.

We thank the reviewer for these comments. We agree that atomic- and molecular-level resolution is needed in the field of peptide-based materials. While we do not have atomic resolution, we employ cryo-EM methods to provide improved resolution of our assemblies, and we have added MD simulations to the revised manuscript to provide additional support for the proposed assembly model.

The main criticisms are:
1. The introduction states that prior examples required restrictions on AAs, but collagen is even more restricted in that in requires the P-HP-G motif that can only be changed to P-X-G in small amounts. This work also seems to require the electrostatic patterning, further restricting the Xs to be Arg and Glu.

We thank this reviewer for this comment. The most significant restriction of collagen-based peptide sequences is that Gly must occupy every third position. There is some flexibility in that both the X and Y positions can be replaced with amino acids other than Pro and Hyp. This, however, is a balancing act. The more changes made to the X and Y

positions, the greater the destabilization of the resulting CMP collagen triple helices. The decrease in stability, however, can be counterbalanced by increasing the length of the CMP (in particular, increasing the number of Pro-Hyp-Gly triads). In our aCMP sequences we have made a significant number of changes to the X and Y positions, while still retaining their ability to form a collagen triple helix. In addition, several studies that demonstrate the ability to modify CMPs with non-canonical residues including peptoids, metal chelating ligands, azaglycine, azidoproline, etc. while still retaining the stability of the collagen triple helix fold (in some cases they further stabilize the triple helix). These studies provide alternative routes to further diversify the CMP amino acid sequence without the need to lengthen the CMP.

With that said, we do concede that our work does have certain restrictions regarding the requirement of having positive and negatively charged amino acids at opposite ends of the CMP sequence. The extent to which these sequences can be modified (e.g., replacing Arg with Lys or decreasing/increasing the number of charged triads at both ends) has not been systematically studied in our system and is something that our lab is actively investigating. However, prior studies on non-lipidated charge-segregated CMPs have shown that changes in the number of charged triads and replacement of charged residues with other charged residues (e.g., aminoproline in place of Arg) do not affect collagen triple helix formation and subsequent assembly (see ref 47, 48, and 49 in the revised manuscript).

There should be some examples where a “free” position is changed to another side-chain to demonstrate that these frameworks can have diverse functionality. Then only it will be general design principle and used for multiple applications. Thus far, they mainly showed the effect of the alkyl chain on the assembly, rather than the effect of the peptide.

[*REDACTED*]

[*REDACTED*]

[*REDACTED*]

In another attempt to demonstrate the diverse side-chain chemistry that can be accommodated within aCMP frameworks, we also synthesized **$C_8$333-Azido**, in which (4R)azidoproline, P_{Az}, replaces the central proline in the X position of the central triad.

$C_8$333-Azido: $(PRG)_3$-POG-P_{Az}OG-POG-$(EOG)_3$

However, unlike **$C_8$333-Ala**, **$C_8$333-Azido** did not assemble into peptide frameworks, but rather into spherical assemblies, which we are currently studying in our lab. Based on this result, we hypothesize that the sterically bulkier side-chain of azidoproline (azide group), compared to alanine, may prevent the lateral packing of aCMP triple helices, which we propose in our proposed working assembly model. Wennemer and colleagues have already reported that (4R) azidoproline form stable triple helices (*J. Am. Chem. Soc.* 2010, *132*, 40, 13957-13959). This result tells us that the identity of the side-chain is an important consideration in assembling porous aCMP frameworks. We are currently investigating this further by synthesizing a family of aCMP derivatives in which the side-chain is systematically lengthened (Ala < Val < Ile and Leu) to determine if there is a limit to how large the side-chain can be before it interrupts the assembly of porous frameworks. In addition to studying the effect of residues with hydrophobic side-chains, we also have plans to investigate the incorporation of charged residues (*e.g.*, Lys, Asp) and Cys in the central triad. We believe that these systematic studies will serve as a foundation for a follow-up manuscript where we systematically explore the free amino acid position. We

believe that a detailed analysis and systematic study of the free position is beyond the scope of this initial, pioneering work. These studies are currently underway in our lab.

In response to this reviewer's comment, we have made the following edits to this manuscript:

The following text (new text is highlighted in yellow):

"Because of the facile ability to modulate the aCMP sequence designs via adjusting the alky chain length and CMP domain, we envision that aCMP frameworks have the potential to serve as a robust, highly tunable, assembly platform for the fabrication of mesoporous materials for a broad range of applications in sensing, separations, and encapsulation."

was changed to (new text is highlighted in yellow):

"Because of the ability to modulate the aCMP sequence design via adjusting the alkyl chain length and CMP domain, we envision that aCMP frameworks may have the potential to serve as a robust, highly tunable, assembly platform for the fabrication of mesoporous materials for a broad range of applications in sensing, separations, and encapsulation. While this work represents an important first step in the development of aCMP frameworks, additional efforts that demonstrate the ability to systematically and rationally tune the physical and chemical features of aCMP frameworks are needed to evaluate the full utility of this new material assembly platform.

2. Though collagen frameworks are novel, it is not clear why making collagen frameworks would be a large advance given that there are many examples of alpha-helical frameworks that are more engineerable than collagen.

We acknowledge this reviewer's concern about the potential advancement of collagen-based frameworks. We believe that both types of frameworks have distinct advantages and limitations. As described in response to comment #1 above, the collagen triple helix can be engineered to a significant extent, including modifying the peptide length, altering amino acids in the X and Y positions, and incorporating various non-canonical residues (*e.g.*, peptoids, azaglycine, and azidoprolines, etc.). There are decades of research that has demonstrated the engineerability of CMPs. These modifications highlight the potential ability to tune the chemical diversity and stability of aCMP frameworks. Furthermore, aCMPs are significantly longer than the alpha helical peptides that are used to assemble alpha-helical frameworks. In this work, the CMP sequences are 27 residues long and can be even longer. Recently, we have synthesized aCMP frameworks comprising aCMPs that have 33 amino acids. Based on the proposed working model, the length of the CMP triple helix domain determines the size of the pores. Therefore, we have the potential ability to systematically tune the pore size simply by adjusting the length of the triple helix

(rise/res. = 0.286 nm for CMP triple helix). In fact, we have strong evidence of this already as demonstrated by our preliminary work involving the assembly of $C_{12}222$ (see our detailed response to comment #1 from Reviewer #2 below). We show that the lattice parameters, which control pore size, can be tuned simply by modulating the CMP triple helix length. All of this is possible because of the *rigidity that is afforded by the hierarchical collagen triple helix fold*, which (we propose) spans the hexagonally arranged hubs. Although CMPs are quite long, the rigidity provided by the collagen triple helix, and their crystallizable antiparallel packing, makes this assembly (crystalline porous frameworks) possible and serves as a route towards creating porous frameworks with tunable mesoporosity – something that is still lacking compared to other porous framework platforms like MOFs and COFs.

We also note that our assembly platform also affords the potential to tune the properties of aCMP frameworks via modulating the hydrophobic domain. In addition to modulating the hydrophobic driving force via changing the length of the lipid tail (as shown in this manuscript), we are also exploring alternative hydrophobic and non-covalent interactions, including aromatic interactions. The ability to explore and toggle *both the CMP and hydrophobic domains* portends to the potential of creating a broad, diverse, and highly modular assembly material class.

While we see that there are advantages to our CMP design, we acknowledge that there are advantages of alpha helical framework designs as well. We view this work as a further example, that goes alongside the amazing work in the alpha helical framework space, of pushing the field of peptide frameworks through *expanding and growing the peptide framework building block library*. Research in both areas will mutually advance and drive the field of peptide frameworks.

To further clarify these advantages, the following text:

"While these strategies have yielded impressive porous architectures, including mesoporous frameworks,[19] and even the emergence of predictive design rules,[21] the restrictions on the peptide building block design stymies the full potential assembly scope, and thus utility, of peptide-based frameworks. The utilization of longer peptides, which exhibit increased chemical, structural, and functional diversity, would move peptide-based frameworks from a niche material class to a robust and expansive assembly platform, akin to MOFs and COFs. While recent efforts have ventured in this direction using multimeric coiled coil building blocks,[31-34] ongoing efforts are needed to expand this assembly design space and broaden the application scope of peptide-based materials.".

was changed to (new text highlighted in yellow):

"While these strategies have yielded impressive porous architectures, including mesoporous frameworks,[19,21,34] and even the emergence of predictive design rules,[21] the

relatively narrow peptide design space for fabricating porous frameworks limits the full potential assembly scope of this materials class. Expanding the peptide building block library represents a key element in broadening the architectural diversity of peptide-based frameworks, and ultimately, diversifying their potential applications. A new approach, which we disclose here, is to utilize lipidated collagen-mimetic peptides (CMPs) that fold into crystallizable collagen triple helices. In this approach, the hierarchical collagen triple helix fold affords the necessary structural rigidity for creating crystalline, porous frameworks. Furthermore, drawing from decades of research on CMPs that demonstrate their chemical and structural diversity,[34-40] CMP-based building blocks have the potential to further propel peptide frameworks from a niche materials class to a predictive and expansive assembly platform,[21] akin to MOFs and COFs. While recent efforts have ventured in this direction of using hierarchical, diverse building blocks (*e.g.*, tetrameric coiled coil building blocks),[34,42-45] ongoing efforts are needed to expand this assembly design space and broaden the application scope of peptide-based materials.

3. Given that these are 3D crystals (stated in Abstract), why isn't there more crystallographic information?

a. What are the space groups?

Unfortunately, the crystals are too small for us to employ crystallographic methods. We have plans to develop methods to increase crystal size with the goal of conducting single-crystal XRD to obtain atomic-level resolution information of our crystals.

b. Can electron diffraction be obtained at other angles to more reliably index the spots?

We attempted to collect microED data on our crystals in collaboration with the NSF BioPacific facility at the University of California, Los Angeles (UCLA), however, the researchers were unable to obtain high-resolution structural data. In response to this, we moved to employing cryo-EM methods to better characterize our assemblies.

c. Can the authors comment on why these cannot grow larger into single crystals?

We have had some success in growing larger crystals (see TEM and DLS data for **C_{12}333** below). Slowing the annealing rate from 90 to 20°C over 72 hours affords larger crystals (crystal size increasing from ~140 nm to ~280 nm) with fewer defects, as evidenced by DLS and TEM (see TEM and DLS data below). We attribute the increase in crystal size to fewer nucleation events that occur when employing slower annealing conditions. Fewer nucleation events lead to larger crystals, which has been previously seen for CMP nanosheets (see ref # 48 in revised manuscript). While we haven't yet explored these routes, we are interested in utilizing hanging drop vapor diffusion and binary solvent

system as ways to increase crystal size. Lastly, future approaches are interesting in altering salt concentrations to weaken lateral interactions, and slowing down the formation of kinetically trapped formations that lead to the multiple crystalline domain particles that we observe, especially for the longer alkyl chain variant (**C_{12}333**).

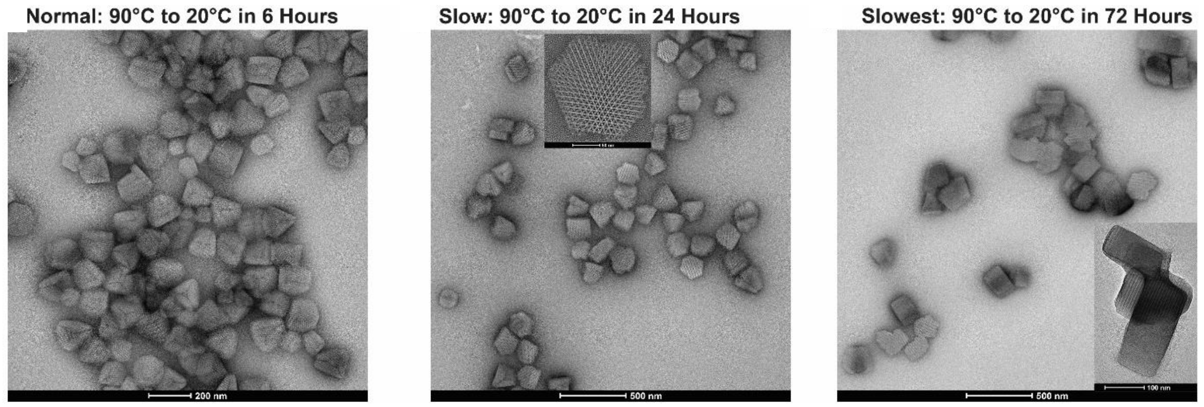

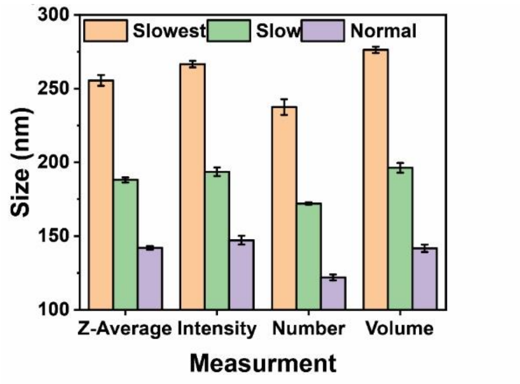

d. While the abstract calls these 3D crystals, most figures and the main text refers to them as 2D crystals. This should be better clarified.

We thank this reviewer for this comment. The “2D crystal” nomenclature was used to refer specifically to the sub-population of thin, planar crystals that we observe for **$C_8$333** (**Figure 3c and 6a**), which are single crystalline and exhibit hexagonally arranged pores that are observed throughout the crystal face (no rotational distortion). We recognize, however, that this is nomenclature is confusing.

To help clarify, we have made the following changes:

- We have removed “three-dimensional (3D)” from the abstract and from the fourth paragraph of the introduction. These places are highlighted in the revised manuscript.

- We have removed the term "2D" in several places that describe the minor population of large, thin crystals that we observed for **$C_8$333**, and replaced this with the term "planar" to highlight that these crystals appear to have a flat, planar surface in which the hexagonal pores are oriented perpendicular to the surface throughout the crystal. These changes are highlighted in the revised manuscript.

4. The paper would be greatly strengthened if they showed that the mesopores could uptake some large molecule guest.

We agree with this reviewer. In addition to Prodan and Nile Red, which we demonstrated their uptake in the original manuscript, in the revised manuscript we disclose evidence that doxorubicin, a large, hydrophobic drug molecule, is taken up by the aCMP crystals, as evidenced by fluorescence spectroscopy analysis. Please see our response to Comment #3 from Reviewer #2 below, where we discuss these results in detail. We have included these results in the revised manuscript. We are very interested in expanding the pore size (currently through lengthening the CMP domain) with the goal of encapsulating large biomolecules including enzymes and other proteins.

Overall, without demonstrating the advantages of a collagen framework over existing peptide frameworks or any application, it is difficult to recommend publication in Nature Communications as is. The main body of the paper focuses on characterization of the structures but falls short of demonstrating the promise of an engineerable class of functional materials. However, the reviewer recognizes that, given the dearth of well-characterized peptide assemblies, that this paper does make an important contribution to the field in that regard.

We thank this reviewer for their feedback. Based on our responses to the comments above, we believe that aCMP frameworks have immense potential to serve as robust and highly modular assembly platforms and will contribute significantly to the future growth and expansion of peptide frameworks. We also believe that the addition of the DOX loading studies and MD simulations of the proposed assembly model significantly enhances the impact of this work.

Specific comments:
1. https://doi.org/10.1021/ja0758990 similar sequence except lacking alkyl tail, published in JACS 2007, design and characterization similar, also this paper has done some MD simulations

We agree that this paper should be included in the manuscript. This paper has been cited (ref. 50) and we have included the following:

The following text:

"Prior work has shown that CMPs, in which positive, neutral, and negatively charged triads are segregated within the peptide sequence can serve as rigid, helical building subunits for the construction of 2D crystalline nanostructures"

was changed to (new text highlighted in yellow):

"Prior work has shown that CMPs, in which positive, neutral, and negatively charged triads are segregated within the peptide sequence can serve as rigid, helical building subunits for the construction of D-periodic collagen fibers and 2D crystalline nanostructures."

2. State the value of the Tm. (Page 4)

The T_m of previously studied charge-segregated CMPs is dependent on their sequence length and amino acid composition. The general protocol for self-assembly is to ensure that the solutions are heated above the melting temperature for the CMP employed. Previous reports heat solutions to 90°C as a consistent approach to ensuring that the temperature is well above any melting temperature of the CMPs employed.

3. The "electrostatic domain dominates" and "hydrophobic and electrostatic domains engaged" panels, the alkyl chain should be attached with N-terminus (the + end). (Fig. 1c)

We thank this reviewer for their careful review. The cartoon figures of the updated Figure 1 have been corrected.

4. PAGE 6/Paragraph 1 – "in line with previous reports" - needs citation

We thank the reviewer for pointing this out. We have added references 47-49.

5. Written as C12333, but in SI and many figures (for example Fig. S1d), the term is written as C12CMP33

We appreciate the careful review of this reviewer. In the revised SI, we have changed all instances of C12CMP333 to $\mathbf{C_{12}333}$, which matches the nomenclature that is used in the main manuscript.

6. In Figure 2h, q3 for C12333 is within the noise levels

We thank the reviewer for their attention to detail. While a small peak is observed (it is more visible in Figure S9c,d), we have adjusted the text to indicate that this peak is close to noise level.

The following text:

"In contrast to **CMP333**, we observe several Bragg peaks in the SAXS profile for **C_{12}333** assemblies that correspond to *d*-spacings of 9.42, 5.37, 4.81, 3.52, and 2.69 nm, respectively (**Figure 2h** and **Table S3**)."

was changed to (new text highlighted in yellow):

"In contrast to **CMP333**, we observe several Bragg peaks in the SAXS profile for **C_{12}333** assemblies that correspond to *d*-spacings of 9.42, 5.37, 4.81*, 3.52, and 2.69 nm, respectively (**Figure 2h, S7c,d**, and **Table S3**; * denotes that, although a faint peak can be observed, the peak for q_3 is close to noise level)."

7. What does "(See above)" on page 16 mean? Does it refer to Figure 3?

The "see above" was inserted based on the two different crystal populations observed for **$C_8$333** as shown in **Figure 3b** and **3c.** To avoid confusion, we have removed this phrase altogether to prevent confusion.

8. C6 is states as close to "tipping point" (Page 10) but given that C8 forms two different morphology, would it be accurate to call it the actual tipping point?

We would like to clarify that the two morphologies of **$C_8$333** have the <u>*same underlying mesoporous hexagonal lattice*</u> assembly with the only exception being that the planar morphology of **$C_8$333** crystals (Figure 3c) is a crystal that does not exhibit rotational distortion and is much larger and flatter in appearance than the majority of the **$C_8$333** crystals. In contrast, the **$C_6$333** assemblies are consistent with previously reported *unlipidated* CMP nanosheets. These assemblies consist of a <u>*tetragonal/pseudo lattice*</u> of antiparallel-packed collagen triple helices that are oriented perpendicular to the plane (see ref. 47-49). The underlying structure is completely different than that of the C8, C10, and **C_{12}333** assemblies.

We thank the reviewer for this comment, because we recognize that this was not communicated clearly in the original manuscript. We have added the following text:

"SAXS/WAXS analysis of **$C_6$333** nanosheets confirms that their underlying structure is consistent with **CMP333** and previously reported CMP nanosheets that are also assembled from non-lipidated, charge-segregated CMPs (**Figure S25e**,**f** and **Table S3**).[47-49] Bragg peaks corresponding to *d*-spacings of 1.02 and 1.44 nm, which closely

matches Bragg peaks obtained for **CMP333**, confirm the underlying tetragonal lattice (1.02 x √2 = 1.44) that comprises their assembly. We note that the SAXS/WAXS profile reveals no peak associated with the length of the aCMP (peak "q_1" in **Figure 2h**), which we attribute to their single-layer construction."

Also, the following text:

"The emergence of nanosheets agrees with our hypothesis that shortening the hydrophobic alkyl chains allows the electrostatic interactions to dictate the final assembly morphology. These results imply that **$C_6$333** is close to the "tipping point" where electrostatic interactions begin to dominate the self-assembly process. "

was changed to (new text highlighted in yellow):

"The emergence of nanosheets, akin to those observed from the assembly of non-lipidated, charge-segregated CMPs,[47-49]including **CMP333**, agrees with our hypothesis that shortening the hydrophobic alkyl chains allows the electrostatic interactions to dictate the final assembly morphology and drive the packing of aCMP triple helices into 2D nanostructures."

9. No yields mentioned of peptides.

We thank the reviewer for this comment. The synthesis of **C_{12}333** was synthesized on a 0.1 mmol scale and a yield of 31.5 mg was obtained (11.1%). **C_{10}333**, **$C_8$333**, and **$C_6$333** were synthesized by equally dividing the resin (0.1 mmol scale). The estimated yields for **C_{10}333**, **$C_8$333**, and **$C_6$333** were 15.9%, 18.6%, and 13.2%, respectively.

The following text was added:

"A yield of 11.1% (31.5 mg) was obtained for **C_{12}333** at 0.1 mmol scale. For the synthesis of **C_{10}333**, **$C_8$333**, and **$C_6$333**, the resin was roughly divided equally (0.025 mmol scale) and estimated yields of 16% (10.9 mg), 19% (12.9 mg), and 13% (9.05 mg) were obtained for **C_{10}333**, **$C_8$333**, and **$C_6$333**, respectively. "

10. "While these strategies have yielded impressive porous architectures, including mesoporous frameworks" – ref 21 (Ganatra et al) also has two examples of mesoporous peptide frameworks.

We thank the reviewer for correctly pointing out that Ganatra *et al.* provided additional examples of mesoporous frameworks.

The following reference (added reference highlighted in yellow) was added to the sentence:

“While these strategies have yielded impressive porous architectures, including mesoporous frameworks,[19,21]…”

11. Discuss why only even length alkyl chains were chosen. In other words, why not use C7 or C9?

Even length alkyl chains were employed in this initial study as a means to more broadly explore the assembly landscape of aCMP frameworks. We do have in mind to explore additional tail lengths, including C5, C7, C9, and C11 to see if these lengths can provide additional fine-tuning of the structural parameters of our peptide frameworks.

12. Authors should make a computational model of the unit cell and superimpose onto the TEM image to show that proposed structure is consistent with the data.

We thank this reviewer for their suggestion. In response to Reviewer #2, we carried out full atomistic MD simulations of the hub-and-spoke hexagonal unit (see new **Figure 7**). From the MD simulations we were able to get physical parameters of the hub-and-spoke assembly model and we superimposed the MD simulated trajectory of the hub-and-spoke model onto the filtered cryo-EM image that is shown in **Figure 6c**. This MD simulated trajectory is drawn to scale with the cryo-EM image. As shown, there is excellent agreement between the MD simulation trajectory of the hub-and-spoke hexagonal unit and the high-resolution cryo-TEM image of **$C_8$333**.

13. In the introduction after “…or have used oligoprolines and/or noncanonical amino acids to rigidify the assembly building block”, there should be added references for 3 recent papers on this topic:
a. J. Am. Chem. Soc. 2025, 147, 20, 17404–17415
b. J. Am. Chem. Soc. 2025, 147, 20, 17433–17447
c. https://pubs.acs.org/doi/full/10.1021/jacs.5c03534

We thank this reviewer for bringing our attention to these publications. We agree that these references should be added. They have been included in the revised manuscript as ref. 31, 32, and 33.

Reviewer #2 (Remarks to the Author):

This manuscript described a crystalline mesoporous peptide framework (MPF) assembled from amphiphilic collagen-mimetic peptides (aCMPs). The authors systematically demonstrated how hydrophobic lipid tails and electrostatic interactions

drive the formation of hexagonally ordered frameworks with tunable pore sizes. Structural characterization is thorough, employing TEM, cryo-TEM, SAXS/WAXS, and CD spectroscopy. The study presents a hub-and-spoke assembly model and contributes to the design of biologically derived porous materials with potential biomedical applications. However, several important issues should be addressed to improve the mechanistic rigor and functional depth of the study.

We appreciate this reviewer's comments. We have done our best to address all of the comments below.

1. Validation of the Hub-and-Spoke Assembly Model
The model is primarily inferred from low-resolution cryo-TEM and SAXS data, without computational or atomic-level structural validation. The authors should consider molecular dynamics or coarse-grained simulations to support their proposed packing geometry and clarify whether collagen triple helices are essential for bridging hubs.

We thank this reviewer for their suggestions of employing other techniques to probe the proposed packing model. We have conducted further experiments to validate the proposed hub-and-spoke assembly model.

[REDACTED]

[*REDACTED*]

The modulation of the pore size and structural parameters of aCMP frameworks via tuning the length of the CMP domain represents the foundation of a follow-up manuscript that we are currently working on (we have also successfully made crystals with longer CMP domains), in which we demonstrate that we can systematically tune the pore size based on our proposed working packing model by extending the length of the CMP domain.

To address this reviewer's comment about clarifying "whether collagen triple helices are essential for bridging hubs," we set up a control experiment where we synthesized a scrambled collagen sequence (**$C_{12}333_{Scr}$**): C_{12}-GGP-GRP-PRR-OOP-GG4-GPP-OEO-GEP-EGG. This scrambled sequence still maintains the charge separation between blocks (positive, neutral, and negative), but within blocks the sequence pattern for collagen triple helices are scrambled. Gly does not occupy every third position, which is a strict requirement for triple helix formation. **$C_{12}333_{Scr}$** (1 mg/mL) was heated and cooled using identical conditions to what was reported and no porous frameworks are observed (see new **Figure S8**). This result provides additional support that collagen triple helix formation is necessary for bridging the hubs and forming crystalline frameworks.

The following text was added:

"In addition to CD, we synthesized and assembled a scrambled derivative, **$C_{12}333_{Scr}$** (**Figure S8a-c**), which preserves the charge separation, but lacks the Gly repeat at every third position – a strict requirement for forming collagen triple helices. Under identical assembly conditions, **$C_{12}333_{Scr}$** does not assemble into crystalline porous frameworks. This result provides additional support that collagen triple helices undergird the formation of aCMP frameworks (**Figure S8d**)."

Lastly, to address this reviewer's comment about using MD simulation to support the proposed model, we initiated a new collaboration with Prof. Bercem Dutagaci at UC Merced, who is an expert in performing MD simulations of biomolecules. MD simulations were carried out on $C_8$333 and C_{12}333 monomers. We initially attempted to see if MD simulations can reveal the assembly of ordered structures from monomers (both free and in their triple helix configuration), but we only observed kinetically trapped aggregates. Therefore, we moved to building the proposed hub-and-spoke assembly model and carried out MD simulations to see if this assembly model remains stable across 100 ns of simulation time. Both **$C_8$333** and **C_{12}333** hub-and-spoke models, which comprise 12 layers of the proposed packing model, were stable over 100 ns (**Figure 7b**). These studies demonstrate that the proposed packing model is stable and the stability of the structure is reflected by interhelical salt bridges within and between the hexagonal hub-and-spoke assembly units (**Figure 7d**). The collagen triple helices were also maintained after 100 ns of simulations as depicted by contact maps and analysis of the dihedral angles (see **Figure 7** and **S33**, **S34** and **S36**). These simulations provide further evidence into the plausibility of our proposed model in which antiparallel-packed collagen triple helices serve as spokes that radiate from hubs. The simulations also reveal the absence of cylindrical micelles as the tails cannot meet "across the hubs" due to steric constraints of the triple helix arrangement. The solvent-exposed hub of the MD simulation may explain the apparent lack of contrast that is observed within the "hubs" in the cryo-EM images (Figure 6c and Figure 6d). In the revised manuscript, we have added new section under the heading: "MD Simulations of the Proposed Assembly Packing Model" that discusses all the MD data in detail. The MD studies helped refine our proposed model and demonstrate the plausibility of this model. With the additional data from C12333 that is detailed above, we believe that this is a current working model that accounts for the data that we have obtained thus far. We recognize that this model may change as we continue to develop and understand this assembly system.

We do want to reiterate that without atomic resolution, we cannot with full confidence state that this is how the molecules are arranged within the framework. In response to this reviewer, we have emphasized more clearly in the revised manuscript that this is a "*working hypothesis*" and it is a "*proposed*" model (see response to the next comment).

2. Lack of Direct Evidence for CMP Bridging

The hypothesis that two aCMP helices bridge each hub pair is speculative without direct visualization. Unless confirmed by cryo-ET or molecular modeling, the structural model should be presented as a working hypothesis rather than a definitive mechanism.

We appreciate this reviewer's comment. While there is some evidence that suggest the presence of two CMP triple helices, as seen in **Figure 4d** (blue arrows) and **Figure 6c**,**d**, which clearly reveal striations, we understand the dissatisfaction of not having atomic

resolution (e.g., crystal structure or atomic resolution cryo-EM structure). However, as described in detail in response to comment #1 from this reviewer (see above), we do have evidence to support that the CMP helices serve as bridges, as evidenced by the **C_{12}222** data presented above and the MD simulation data, which show that the proposed packing model is stable. Nevertheless, as stated above, we recognize that these are not definitive proof of the assembly model. In the revised manuscript we have clarified that the proposed model is not definitive but rather a "working hypothesis." To reflect this, we have made the following changes:

In the revised manuscript, the following sentence:

"In this model, CMP domains serve as "spokes" that radiate in six directions from cylindrical micelle-like "hubs," where lipid domains pack together via hydrophobic effect."

was changed to (new text highlighted in yellow):

"In this proposed model, which serves as our current working hypothesis, CMP domains serve as "spokes" that radiate in six directions from "hubs," where lipid domains pack together via hydrophobic effect."

We have also added "proposed" several times throughout the revised manuscript (these are highlighted as well).

3. Missing Functional Demonstration of Guest Encapsulation
Although MPFs are proposed as mesoporous materials capable of encapsulating hydrophilic and hydrophobic molecules, no experimental data are provided. A simple proof-of-concept guest loading/release experiment (e.g., dyes or proteins) would significantly strengthen the study.

We thank this reviewer for this comment. **Figure 5** reveals the results of two fluorescent molecules that were taken up by aCMP frameworks. Based on the fluorescence spectra in **Figure 5a** and **5d**, we demonstrated that Prodan and Nile Red, which are solvatochromic fluorescent membrane dyes, both embed within hydrophobic pockets within aCMP frameworks (as observed by the blue-shift in fluorescence signal for Prodan, and fluorescence emission for Nile Red).

In the revised manuscript, we provide another example involving the uptake of a large molecule within aCMP crystals. To demonstrate their potential for loading therapeutic agents, we carried out the encapsulation of doxorubicin (DOX), an anti-cancer drug molecule. A new panel, added to Figure 5 (**Figure 5e**) and new **Figure S26** reveals an increase in fluorescence emission that is observed when we mix DOX with our aCMP frameworks (1:10 molar ratio of DOX:aCMP for **Figure 5e**). The increase in fluorescence

emission is consistent with previous reports that observe an increase of fluorescence emission of DOX when DOX partitions into hydrophobic environments (see new references 59, 60, 61). The increase in fluorescence upon partitioning of DOX within hydrophobic pockets is attributed to reduction of dynamic quenching, or collisional quenching, due to the shielding from collisions with water within the hydrophobic environment. As we increase the amount of DOX loading, we observe a relative decrease in DOX emission increase (compared to the DOX only control), likely due to self-quenching as DOX molecules become more crowded within aCMP frameworks. We understand that there is still a lot of work to be done to characterize where the molecules are congregating within the framework, and systematic release studies are still needed, but we believe that these studies can best be served by follow-up manuscript(s) where we systematically study how tuning the pore chemistry/pore size influences their uptake of different molecules (see responses above where we are systematically studying the effect of amino acid diversity and CMP domain length on aCMP frameworks). We believe that these initial three fluorescent molecules (Prodan, Nile Red, and Doxorubicin) demonstrate that the frameworks can encapsulate molecules.

We have added the following text on the DOX loading study to the revised manuscript:

"The successful uptake of hydrophobic molecules within hydrophobic domains of aCMP frameworks prompted us to examine the potential of aCMP frameworks for encapsulating therapeutic cargo. **$C_8$333** frameworks (10 μM aCMP concentration) were incubated in the presence of doxorubicin (DOX), an anti-cancer drug at various DOX:aCMP molar ratios (**Figure 5e** and **S26**). At low DOX:aCMP molar ratios (1:10), we observed a 174% increase in fluorescence signal at 593 nm compared to the emission of a solution containing the same DOX concentration without assembly (**Figure 5e** and **S26g**). The increase in fluorescence is attributed to the partitioning of DOX to the lipophilic environments within aCMP frameworks, which shields DOX from collisional quenching with water, as observed in previous reports.[59-61] We note that as DOX loading increases, we observe a relative decrease in fluorescence compared to the DOX-only control, which is likely due to self-quenching as more DOX is loaded within confined hydrophobic domains (**Figure S26**). TEM images confirm that the aCMP frameworks remain stable after incubation with DOX (**Figure S27**). Altogether, these fluorescence studies demonstrate that aCMPs comprise lipophilic domains that are capable of encapsulating hydrophobic small molecules, including biologically relevant cargo (DOX). Moreover, these results highlight the increased crystallinity of the hydrophobic environment for porous frameworks that are assembled from aCMPs with shorter alkyl chain lengths (*e.g.*, **$C_8$333**)."

4. No Stability Evaluation Under Physiological Conditions
All characterizations were conducted in MES buffer at pH 6.0. The structural integrity of

MPFs at pH 7.4, in serum, or under enzymatic conditions should be tested to support claims of biomedical relevance.

We thank the reviewer for this comment. In response to this comment, we carried out stability studies of **$C_8$333** frameworks in pure water and in phosphate buffered saline (PBS; pH 7.4). We observed that while **$C_8$333** crystals remain stable in pure water (new **Figure S18**), the increase in salt concentration of PBS buffer led to the disassembly of **$C_8$333** in PBS. This behavior has been observed with the unlipidated nanosheet assemblies. Since electrostatic interactions are integral to assembly formation, high ionic strength weakens these interactions leading to disassembly.

We added the following text to the revised manuscript:

"To test the stability of **$C_8$333** crystals in other solvents, pre-assembled **$C_8$333** frameworks, originally in MES buffer (pH 6.0), were exchanged in pure water and phosphate buffered saline (PBS; pH 7.4). While **$C_8$333** assemblies were stable in pure water, we observed degradation of **$C_8$333** assemblies in PBS buffer, likely due to shielding of the inter-triple helix electrostatic interactions that maintain the structural integrity of the assembled architecture (**Figure S17**). Future efforts involve improving the stability of aCMP frameworks at physiological ionic strength (an important consideration for their biomedical application) via post-assembly modification."

5. Potential Contribution from Free Fatty Acids
For long-chain aCMPs (e.g., C12), the role of lipid tail self-aggregation cannot be ruled out. A control experiment with free fatty acid plus non-lipidated CMP would help decouple lipid-driven assembly from peptide-specific interactions.

We thank the reviewer for this suggestion. In response to this comment, dodecanoic acid and CMP333 were mixed in a 1:1 molar ratio, heated to 90°C and cooled using identical conditions (1 mg/mL peptide concentration and 20 mM MES buffer) that were employed with the lipidated variant. TEM images (see new **Figure S21**), collected after two weeks of assembly time, confirm the absence of aCMP frameworks, indicating that the covalent tethering associated with the amphiphilic nature of aCMPs is necessary for forming the porous architecture. Interestingly, we observe spherical particles that appear to have distinct layers. This architecture may be due to the 2D assembly of CMPs with free fatty acid at the interface between the layers.

We have added the following text to the revised manuscript:

"Lastly, in an attempt to decouple the lipid-driven and CMP-driven interactions, octanoic acid was mixed with non-lipidated **CMP333** (1:1 molar ratio; ~ 1 mg/mL **CMP333**) and the mixture was heated and cooled using the same assembly protocol described above. TEM images confirm the absence of porous frameworks, indicating that the amphiphilic nature

of aCMPs, in which lipid and CMP domains are covalently linked, is necessary for directing the assembly of aCMPs into the porous frameworks (**Figure S21**). Interestingly, the decoupling of aCMPs yields spherical particles with clearly defined concentric layers (**Figure S21**).”

6. Discrepancies Between TEM-FFT and SAXS-Derived Lattice Constants
Minor differences between d-spacings obtained via TEM and SAXS are observed. The authors should tabulate these values and briefly discuss possible sources of error, such as projection effects or differences in hydration state.

We thank this reviewer for this comment. While SAXS provides global average of interplane distances for all crystals in the solution, the values obtained from the TEM data (Figure 2e, for example) is taken from a single image and is subject to projection effects and other artifacts including uranyl acetate stain treatment, electron beam damage, and drying effects.

We have added the following text to disclose potential sources for discrepancy between *d*-spacing values obtained from SAXS and TEM:

“These values, which represent global average lattice parameters of crystals in their native, hydrated state, differ slightly from the values obtained from FFT analysis of the crystal depicted in **Figure 2e**. The minor discrepancy in d-spacings likely stems from multiple sources including projection effects, uranyl acetate staining, and drying effects, which are all associated with TEM analysis.”

Reviewer #3 (Remarks to the Author):

The work presented is an exciting direction in the importance of peptides/proteins in the design and creation of new nanostructures in self-assembled materials that directly highlights the importance of the peptide fold/secondary structure and quaternary structure in the display of chemistry. The combination of amphiphilicity, electrostatic interactions, and specificity in display of chemical functionality is what gives rise to the beautiful lattice structures observed in the new library of aCMPs. The background is a nice discussion of MOFs, COFs, and the opportunities that peptides/proteins provide in construction of (nano) porous materials. Other aspects of the manuscript that I particularly like is the direct comparison between CMPs and aCMPs (to directly observe effects of aliphatic tail inclusion in constituent molecules). I also appreciate the kinetic study showing that with cooling temperature, the porous lattices do not form until the

CMPs fold for a directional display of both the hydrophobic tails and the specific spatial display of the charged amino acids in the CMP sequence. The authors have a new, exciting platform with which they can probe the importance of both the CMP design and the attached alkyl (or other) functionalities in making new structure with potential function. The manuscript should be published in Nature Communications after addressing the minor comments below.

We appreciate this reviewer's enthusiasm for the work. We see this initial work as the foundation for a new assembly platform. We are excited to expand the aCMP design with new CMP and hydrophobic domains (e.g., aromatic groups, conjugated drugs, etc.).

My intuition tells me that the length of the aliphatic tail would lead to the opposite trend in aliphatic domain gel state/crystalline state. In membranes, shorter amphiphiles are harder to crystallize/not possible to crystallize. It seems there is literature precedent for the crystallization of short alkyl tails when conjugated to CMPs or other co-assembling molecules. I'm hoping the authors could briefly discuss this in the context of the lattice since, clearly, the alkyl tails aren't residing in a pure hydrophobic layer such as in a membrane.

Yes, we also expected that the longer aliphatic tails would lead to assemblies with hydrophobic domains that are in a more crystalline state. In the revised manuscript we have carried out MD simulations to help uncover any new insights into the assembly. MD simulations of the proposed hub-and-spoke assembly model reveal the absence of a "cylindrical micelle-like" packing as the length scale of the lipid tails are simply not long enough to bridge the hub distance. MD simulations also reveal that the lipid tails are more mobile for **C_{12}333** compared to **$C_8$333**, in line with the fluorescent studies involving the solvatochromic probes. Inspection of the tails from MD trajectories suggests that the tails lie flat along the inner pore of the hubs and bend back towards the CMP domain. As the reviewer suggests here, there does not seem to be any "pure hydrophobic layer." We are unsure, however, the exact reasoning for this and why this would lead to a more crystalline state for **$C_8$333** lipid tails. We have other hydrophobic domains that we are attaching to CMPs that can possibly shed more light on this.

What keeps the lattices from growing in the c/z thickness direction? Or, do the sheets actually have multiple layers of the model structure in Figure 2, 3? The molecules that lead to the pyramidal-like particles seem to have thicker walls (while Figure 6 and the C8333 lattices are very thin). I agree with the proposed model for the molecules packing within the 2D structure with hydrophobic hubs and CMP spokes. The careful analysis of the TEM, cryo-TEM, and SAXS data very much nails down how the molecules lead to the same symmetry of lattice but with slightly different spacings. However, the nodes/hubs of hydrophobic alkyl chains provides many sites for hydrophobic

interactions and the attachement of aCMPs to the hubs or for a second layer of lattice to form directly on top of the first and so in. In fact, as mentioned above, the more particle-like assembly of the aCMPs with longer tails (10 and 12 carbons) in Figures 2 and 3 suggest that the walls of the particle, made of the same hexagonal-like lattice structure, are multilayer/thicker. If the longer alkyl tails are less crystalline, perhaps they lead to more exposed hydrophobic alkyls chains that, in turn, provide the anchoring sites for multilayer lattices to grow.

For all aCMPs we believe that they are composed of multiple layers as suggested by this reviewer. **C_{12}333**, **C_{10}333**, **$C_8$333**, all assemble into 3D crystals, with multiple layers of the hub-and-spoke model on top of one another (as shown in **Figure 7a,b** in the revised manuscript). We believe that these crystals assemble into hexagonal bipyramidal crystals, which is most noticeable for **$C_8$333** assemblies (see **Figure S29**). However, some of the **$C_8$333** crystals, especially at longer timepoints appear to be planar as shown in **Figure 3c**. We initially thought that there would be a large hydrophobic cylindrical micelle-like hub and that could continue to promotion of growth in the z-direction, however, MD shows that the hubs are actually solvent accessible and do not consist of a purely hydrophobic domain. This may limit the growth of crystals in the z-direction and promote their lateral assembly (like the C8333 crystal in **Figure 3c**).

As this reviewer suggests C12333 appears to be more particle-like and this could be attributed to more exposed hydrophobic alkyl chains compared to the more crystalline lipid domains of C8333. Additional experiments, with variations in the lipid length/crystallinity may provide insight on this further.

A similar point to the above is the more particle-like morphology observed for the longer alkyl tails as opposed to the clearly 2D nature of the shorter tails. The model comparing the two different C8333 lattices in Figure 6e may provide a clue as to why these particle (if they are, in fact, particles) form in that the longer alkyl tails on the edges of growing lattice sheets could be quite flexible as the next layer of aCMPs attach, thus acting like a hinge and allowing a new sheet to grow at a defined angle relative to the parent sheet (or an existing sheet to attach at an angle to a parent sheet). Would the authors please comment on the apparent particle nature of the longer alkyl tail lengths/does any AFM exist to show the apparent 3D nature of these structures?

We thank the reviewer for this comment. The idea that the longer alkyl tails of aCMPs serving as hinges for new growth at a defined angle is an interesting idea and may contribute to the crystal shapes that we observe. If we slow the cooling down for $C_8$333, we observe significantly more growth in the z-direction (see Figure S29). We believe that this is due to the number of nucleation events that occur in solution. The more "particle-like" morphology is a result of fewer nucleation sites and faster growth kinetics in the z-

direction to make the hexagonal bipyramidal shape that we see when slow-cooling. When we have more nucleation events (cool the solution more quickly), there is less monomers available to grow on the surface of the crystal in the z-direction and which is why the $C_8$333 crystals appear to be flatter (silver dollar-like). At this moment we are unsure what mechanisms govern the competition between lateral growth and growth in the z-direction.

We did attempt AFM, however, we were not able to obtain clear images. We believe that there needs to be methods to anchor these crystals on the surface of mica. We did not spend too much time on AFM as we moved to other characterization techniques that better resolved the hexagonal structure (cryo-TEM). We would like to revisit AFM again and/or SEM to provide further characterization of the 3D nature of the crystals.

I don't think any additional experiments are needed for the current work although I am keen on finding out the differences in assembly when different pH conditions are used. Turning off one of the complementary charges, for example, would allow a direct observation of the hydrophobic assembly as suggested in Figure 1. With respect to Figure 1, it looks more like a first figure in a proposal as opposed to a manuscript since it suggests future work and what is enabled by the aCMP system. Figure 1 should be revamped to focus on the aCMPs herein and the importance of complementary charged and alkyl chain length. Already these first examples provide rich assembly/nanostructure formation. The shape shifting and hydrophobic interactions dominating panels can be saved for future aCMP work. Also, the "?" in the novel architecture panel certainly can be removed! A revamped Figure 1 would help the reader focus on the interacting molecular attributes that give rise to the beautiful behavior presented.

We thank this reviewer for their comments. We appreciate the reviewer's suggestion for improving **Figure 1**. In the revised manuscript, we have modified **Figure 1** to focus on the aCMP design and its self-assembly into extended mesoporous frameworks.
